# Generation of equine‐specific local antibiograms: reproductive tract of mares and fillies in Western Australia

**DOI:** 10.1111/avj.70079

**Published:** 2026-03-28

**Authors:** JKH Hoong, C Boyd, C Secombe, D Byrne

**Affiliations:** ^1^ School of Veterinary Medicine, Murdoch University Perth Western Australia Australia

**Keywords:** antimicrobial resistance, antimicrobial stewardship, empirical antibiotics, endometritis, mares/fillies, reproductive tract

## Abstract

**Introduction:**

Antimicrobial resistance (AMR) may lead to increasing inefficacy and treatment failure of bacterial infections in the future. Antimicrobial stewardship (AMS) programmes are critical to ensuring the continued efficacy of available antimicrobials. There is a lack of published evidence‐based data on the susceptibility of isolates from common reproductive infections encountered in mares. This study aims to provide equine veterinarians in Western Australia (WA) with the most appropriate first‐line empirical antimicrobials for infections of the mare reproductive tract.

**Materials and methods:**

A retrospective study was conducted on reproductive tract swab/fluid culture and sensitivity results of mares and fillies in WA from July 2015 to June 2020. An algorithm was generated using R to create an antibiogram for the mare reproductive system.

**Results:**

The antibiogram included 767 reproductive tract isolates. *Escherichia coli* (*E. coli*) (277 of 767; 36%) was the most commonly isolated, followed by *Streptococcus* (240 of 767; 31%). Gram‐negative isolates were most susceptible to enrofloxacin (84%), followed by ceftiofur (75%), gentamicin (72%) and tetracycline (65%). Gram‐positive isolates were most susceptible to ampicillin (86%), followed by penicillin (83%), ceftiofur (83%) and trimethoprim‐sulfonamide (TMS, 76%). The overall susceptibility of ceftiofur decreased across both Gram‐negative and Gram‐positive isolates during the time periods 2015–2017 and 2018–2020 from 79% to 69% and 91% to 75%, respectively.

**Discussion:**

Gram‐negative isolates were most susceptible to the high importance antimicrobials ceftiofur and enrofloxacin. However, these antimicrobials should be used judiciously only after culture and sensitivity testing have been performed, in accordance with the Australian Veterinary Prescribing Guidelines, as part of AMS. This study found that Gram‐negative organisms are most susceptible to first‐line empirical treatment with tetracycline if E. coli is suspected, or gentamicin when in doubt. Gram‐positive isolates were most susceptible to first‐line empirical treatment with ampicillin, penicillin or TMS.

Regular updates to the antibiogram are required to provide contemporary empirical guidance for the use of antimicrobials.

**Abbreviations**
AMR: Antimicrobial Resistance
AMS: Antimicrobial Stewardship
CLSI: Clinical and Laboratory Standards Institute
*E. coli*
: *Escherichia coli*
MDR: Multidrug Resistant
*S. zooepidemicus*: *Streptococcus equi* subspecies *zooepidemicus*
TMS: Trimethoprim‐sulphonamide
WA: Western Australia

Many reproductive diseases in the mare, such as endometritis, retained placenta, puerperal metritis and bacterial placentitis, may necessitate antimicrobials. Endometrial fluid samples from mares show increasing antimicrobial resistance (AMR) across a range of antibiotic classes.^1^ High rates of enrofloxacin resistance have been reported in *Escherichia coli* (*E. coli*) from mare uterine cultures.^1^ Multidrug‐resistance (MDR) has also been described for *E. coli*, commonly identified as a cause of uterine infection in horses.^2^

AMR is increasingly gaining global attention due to the development of resistant bacteria in food‐producing species, placing antimicrobial use in veterinary medicine under scrutiny.^1^, ^3^, ^4^, ^5^, ^6^, ^7^ Failure to treat bacterial infections due to AMR may result in increased morbidity and mortality.^1^, ^8^, ^9^

Antimicrobial stewardship (AMS) is the balance between the prudent use of antimicrobials and requirements to treat the presenting clinical condition.^5^ Prudent use of antimicrobials encompasses optimal drug choice, correct dose and duration and avoidance of inappropriate and excessive use to prevent further contributions to AMR, a future in which effective antimicrobials become unavailable.^6^, ^9^, ^10^

AMS programmes have not been extensively implemented in veterinary practices.^11^ AMS programmes implemented in human hospitals have been shown to improve outcomes and effectively slow the spread and emergence of AMR.^11^, ^12^ When a clinical examination is performed on a horse with a suspected non‐life‐threatening bacterial infection, ideally a sample should be obtained for culture and sensitivity testing before selecting a suitable antimicrobial agent.^1^, ^5^, ^6^, ^7^, ^9^, ^13^, ^14^, ^15^ Such a procedure would greatly benefit the horse through targeted treatment and would add to the data collected for future patients.^5^ Even so, it may not be feasible in many clinical situations. Common clinical scenarios should be identified, and first‐line treatment protocols should be based on culture and sensitivity results from previous patients, collated into an antibiogram.^5^, ^6^, ^7^ Such a protocol enables targeted treatment and serves as a foundation for empirical antimicrobial selection in future cases.^5^, ^7^, ^16^, ^17^ An antibiogram justifies the antimicrobial chosen and can be used as an AMR educative tool.^18^ It also provides an impartial viewpoint, not influenced by the veterinarian but by scientific testing and data collection.^18^ The goal is to ensure use of antimicrobials without any beneficial effect does not occur and to safeguard available antimicrobials used in the equine clinical practice.^6^

Despite the frequent use of antimicrobials in horses, clear data on their use in this species are lacking.^1^, ^6^, ^8^, ^13^ To date, there is no published literature on equine‐specific antibiograms in Western Australia (WA) for the mare reproductive system to inform empirical antimicrobial treatment decisions.^8^, ^19^, ^20^, ^21^ Antibiograms are required to evaluate local data and enable clinicians to be aware of local AMR status and trends.^8^, ^17^, ^18^, ^20^

The aim of this study was to generate an antibiogram for bacterial infections of the mare reproductive tract using data from the predominant WA laboratory at the time to guide equine veterinarians on appropriate first‐line empirical antibiotic selection. A secondary objective was to assess changes in susceptibility patterns to the high‐importance antimicrobial ceftiofur over time.

## Materials and methods

### Design

This retrospective study was based on reproductive tract fluid/swab culture and sensitivity results of fillies and mares from July 2015 to June 2020. Inclusion criteria were samples from any location in the female reproductive tract. Samples from the male reproductive tract or from horses of unidentified sex were excluded. Bacterial isolates that were unidentifiable, or were believed to be contaminants based on cytology were also excluded. The bacteriological isolates were submitted to a single local, nationally accredited commercial laboratory in WA (Vetnostics WA) by a total of 55 clinical equine practices (including referral and first opinion practices) in WA. Data from the laboratory were provided to the researchers as a single spreadsheet. The equine‐specific local antibiogram was generated using R (in R studio).

### Data collection

Samples submitted to Vetnostics WA from July 2015 to June 2020 were tested against various antimicrobials, with data on susceptibilities obtained via spreadsheet (Excel, Microsoft Office). The Kirby‐Bauer test was performed for each isolate according to Clinical and Laboratory Standards Institute (CLSI) guidelines. Mueller Hinton agar plates (with 5% blood for fastidious isolates) were used. Antimicrobials tested against were ampicillin 10 μg, neomycin 30 μg, penicillin 10 μg, tetracycline 30 μg (as a proxy for oxytetracycline), trimethoprim‐sulphonamide (TMS) 25 μg, gentamicin 10 μg, ceftiofur 30 μg and enrofloxacin 5 μg. There were no changes to Vetnostics WA's laboratory methodology in microbiology during this time.

### Data analysis

Data were imported into the statistical software language R version 4.1.0 to conduct data analysis.^22^ Reproductive isolates were located by searching for key terms such as uterine, vaginal, endometrial, endometritis, clitoral, cervical, placenta, premate, mare and vulva. Bacterial isolates were grouped into genus/species. R was used to calculate the susceptibility of each bacterial genus/species to individual antimicrobials as proportions and percentages.

The antibiogram was constructed in accordance with the guidelines of the CLSI and the Australian Commission on Safety and Quality in Health Care.^12^, ^17^ However, duplicates were not eliminated by including only the first isolate per patient as per the guidelines. Based on the guidelines, the susceptibilities for at least the five most commonly isolated species should be reported, along with species that have 30 or more isolates tested. The decision was made to combine *Enterobacter, Pseudomonas, Klebsiella, Serratia, Streptococcus, Staphylococcus* and *Enterococcus* species into genera for a more meaningful antibiogram. Only *E. coli* was left at the species level. *Klebsiella* and *Serratia* species were also included in the antibiogram, although only 29 isolates were tested. The eight most commonly cultured isolates were included in the antibiogram. Gram‐negative and Gram‐positive isolates were colour‐coded, and isolates were listed in descending order of frequency.^12^, ^20^ The number and proportion of each bacterial isolate reported were stated. The list of antimicrobials tested against, along with the susceptibilities in percentage and number of isolates tested for that particular antimicrobial, were documented in the antibiogram. Intermediate category isolates were classed as resistant. The antimicrobials tested against were also colour‐coded based on the category of importance. The algorithm used to generate the results for the antibiogram, along with brief explanations, is provided in Appendix S1.

Proportions of ceftiofur susceptibility for Gram‐positive and Gram‐negative isolates between time periods 2015–2017 and 2018–2020 were compared by chi‐squared tests in R, with significance set at P < 0.05.

## Results

There were 884 equine reproductive tract isolates, of which 106 were from species that did not have enough isolates to report in the antibiogram, 10 were unidentified and 1 was identified as an environmental contaminant. A total of 767 equine reproductive tract isolates were included in the antibiogram (Table 1). Of these, 416 of 767 (54%) were Gram‐negative isolates, and 351 of 767 (46%) were Gram‐positive isolates (Table 1). Out of all the isolates, *E. coli* (277 of 767; 36%) was the most commonly isolated, followed by *Streptococcus* species (240 of 767; 31%), *Staphylococcus* species (56 of 767; 7%), *Enterococcus* species (55 of 767; 7%), *Enterobacter* species (42 of 767; 5%) and others (97 of 767; 13%) (Table 1). *E. coli* was the predominant Gram‐negative isolate, and *Streptococcus* was the predominant Gram‐positive isolate.

**Table 1:** Cumulative antibiogram for common mare reproductive tract isolates in Western Australia (2015–2020).

Isolate type	Number of isolates	% total	Ampicillin^a^	Neomycin	Penicillin	Tetracycline	Trimethoprim‐sulphonamides	Gentamicin^b^	Ceftiofur^c^	Enrofloxacin^a^, ^b^, ^c^
All isolates	767	100%								
Gram‐negative isolates										
Escherichia coli	277	36%	48%	29%	R	77%	59%	73%	86%	95%
			273	275	277	277	277	277	277	273
Enterobacter species	42	5%	R	38%	R	57%	62%	62%	60%	88%
			40	40	42	42	42	42	42	40
Pseudomonas species	39	5%	3%^d^	38%	0%^d^	23%^d^	10%^d^	64%	8%^d^	26%^e^
			39	39	39	39	39	39	39	39
Klebsiella species	29	4%	R	62%	R	69%	55%	66%	62%	69%
			29	29	29	29	29	29	29	29
Serratia species	29	4%	R	72%	3%	10%	S	97%	90%	68%^f^
			28	29	29	29	29	29	29	28
Gram‐negative	416	54%	33%	36%	0%	65%	57%	72%	75%	84%
			409	412	416	416	416	416	416	409
Gram‐positive isolates										
Streptococcus species	240	31%	99%	0%^g^	98%	19%	91%	0%^h^	S	21%^i^
			238	238	240	240	240	240	240	238
Staphylococcus species	56	7%	26%	84%	27%	75%	88%	82%	95%	96%
			54	55	55	55	56	56	55	54
Enterococcus species	55	7%	91%	R	71%	60%	R	R	R	9%^j^
			55	55	55	55	55	55	55	55
Gram‐positive	351	46%	86%	14%	83%	34%	76%	13%	83%	31%
			347	348	350	350	351	351	350	347

Antibiogram key: **91%**, >90% of susceptible isolates; **S**, Susceptible/intrinsically susceptible;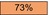, 70%–89% susceptible isolates;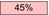, <70% susceptible isolates;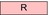, Intrinsically resistant;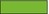, Low‐importance antimicrobials;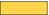, Medium‐importance antimicrobials;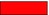, High‐importance antimicrobials.

Notes regarding intrinsic resistance, as per CLSI guidelines, relative to Table 1. Enterobacter cloacae complex (includes Enterobacter asburiae, Enterobacter cloacae and Enterobacter hormaechei) is intrinsically resistant to ampicillin. Pseudomonas aeruginosa is intrinsically resistant to ampicillin, tetracyclines and trimethoprim‐sulfamethoxazole. Klebsiella aerogenes is intrinsically resistant to ampicillin. Klebsiella pneumoniae, Klebsiella oxytoca and Klebsiella variicola are intrinsically resistant to ampicillin and ticarcillin (Penicillin). Serratia marcescens is intrinsically resistant to ampicillin. Enterococcus faecalis, Enterococcus faecium, Enterococcus gallinarum and Enterococcus casseliflavus are intrinsically resistant to cephalosporins (ceftiofur), aminoglycosides (neomycin, gentamicin) and trimethoprim‐sulfamethoxazole.

^a^ An antibiotic product is not registered for use in this species, check legal obligations before using.

^b^ Prohibited if the food chain is a possible destination.

^c^ Use only in an individual animal in exceptional circumstances, after culture and susceptibility testing, if there are no alternatives.

^d^ 28 out of 39 were intrinsically resistant.

^e^ 22 out of 39 were of intermediate resistance but grouped with resistant.

^f^ 9 out of 28 were of intermediate resistance but grouped with resistant.

^g^ 227 out of 238 were intrinsically resistant.

^h^ 229 out of 240 were intrinsically resistant

^i^ 167 out of 238 were of intermediate resistance but grouped with resistant.

^j^ 37 out of 55 were of intermediate resistance but grouped with resistant.

The overall susceptibility profiles of various isolates are shown in Table 1. Gram‐negative isolates had the highest susceptibility to enrofloxacin (84%), followed by ceftiofur (75%) and gentamicin (72%) (Table 1). Gram‐positive isolates were most susceptible to ampicillin (86%), followed by penicillin (83%) and ceftiofur (83%) (Table 1). A higher rate of AMR was identified in Gram‐negative isolates compared to Gram‐positive isolates. More than 70% of Gram‐negative isolates were susceptible to three antimicrobials (gentamicin, ceftiofur and enrofloxacin), compared to four (ampicillin, penicillin, TMS and ceftiofur) for Gram‐positive isolates.

Susceptibility to ceftiofur by time period for Gram‐negative and Gram‐positive isolates was evaluated in 766 samples. An isolate not tested against ceftiofur was excluded. The overall susceptibility of ceftiofur decreased across both Gram‐negative and Gram‐positive isolates during the time periods 2015–2017 and 2018–2020, from 79% to 69%, and 91% to 75%, respectively (Table 2).

**Table 2:** Susceptibility of Gram‐negative and Gram‐positive mare reproductive tract isolates to ceftiofur during time periods 2015–2017 and 2018–2020

Isolate	Susceptibility by time period		P
	2015–2017	2018–2020	
Gram‐negative isolates	192/244 = 79%	118/172 = 69%	0.027
Gram‐positive isolates	160/176 = **91%**	131/174 = 75%	<0.001

^a^ Key as per Table 1.

## Discussion

This paper describes the generation of an antibiogram based on the reproductive tract isolates of mares and fillies in WA from July 2015 to June 2020. The dominance of *E. coli* (36%) and *Streptococcus* species (31%) as causative agents of bacterial endometritis is consistent with peer‐reviewed literature.^1^, ^14^, ^23^, ^24^, ^25^, ^26^ Due to the additional cost required to speciate *Streptococcus*, most *Streptococcus* isolates were not speciated. However, the most significant proportion can be assumed to be made up of *Streptococcus equi* subspecies *zooepidemicus* (*S. zooepidemicus*), as substantiated by others.^1^, ^14^, ^23^, ^24^, ^25^, ^26^ The high susceptibility of *S. zooepidemicus* to ampicillin, penicillin and ceftiofur across multiple studies is also supported in the antibiogram, further supporting this assumption.^14^, ^19^, ^26^, ^27^, ^28^ In some studies, *E. coli* was the most common bacterial pathogen isolated, similar to the results of this paper, while some reported *S. zooepidemicus* more frequently.^16^, ^19^, ^24^

*E. coli* had low susceptibility to ampicillin (48%), neomycin (29%) and TMS (59%), as shown in Table 1; ampicillin and TMS are commonly used systemic antimicrobials used to treat bacterial endometritis.^15^, ^27^ This trend is corroborated by the findings of Davis et al., where *E. coli* was most resistant to ampicillin, TMS and oxytetracycline.^19^

In the present study, *S. zooepidemicus* had low susceptibility to neomycin (0%), tetracycline (19%), gentamicin (0%) and enrofloxacin (21%) (Table 1). Gentamicin and enrofloxacin are common antimicrobials used to treat bacterial endometritis.^14^, ^15^, ^27^ This finding is supported by other studies. Johns and Adams (2015) found that *S. zooepidemicus* isolates were most resistant to tetracyclines, gentamicin and enrofloxacin, and Mitchell et al. observed that *S. zooepidemicus* isolates from equine uteruses had low susceptibility to gentamicin and enrofloxacin.^1^, ^16^

Ceftiofur is widely used as a systemic and intrauterine antimicrobial for the treatment of bacterial endometritis.^13^, ^27^ *E. coli* isolates have shown increasing resistance to ceftiofur due to extended‐spectrum β‐lactamase production.^1^, ^2^, ^14^, ^15^, ^16^, ^27^ Furthermore, the administration of ceftiofur to horses is a risk factor for the identification of resistant isolates in both humans and horses.^1^

Concerningly, there is a significantly lower proportion susceptible to ceftiofur in the later time period (2018–2020) for both Gram‐negative and Gram‐positive groups (Table 2). Therefore, the use of ceftiofur, a third‐generation cephalosporin, as a first‐line empirical treatment for horses should be avoided. A critically important World Health Organisation designated compound should only be used for MDR infections, life‐threatening conditions or when culture and sensitivity results show no susceptibility to lower importance drugs.^5^, ^13^, ^14^, ^27^, ^28^

Gram staining and cytological evaluation can be performed in house to identify Gram‐positive or Gram‐negative cocci/rods while waiting for culture and sensitivity results.^29^ Low importance antimicrobials should be used in most cases.

Apart from a life‐threatening condition where any further delay in appropriate antimicrobial treatment may increase mortality and morbidity, empirical first‐line antimicrobials of lower importance should be used initially.^1^, ^8^, ^15^ In accordance with Australian Veterinary Prescribing Guidelines, high priority antimicrobials such as ceftiofur and enrofloxacin should be used judiciously, only when indicated by culture and sensitivity testing or in unique circumstances recommended by expert guidelines.^5^, ^6^, ^9^, ^28^ This study found that Gram‐negative isolates are most susceptible to first‐line empirical treatment with tetracycline if *E. coli* is suspected, or gentamicin when in doubt (Table 1). Tetracycline (77%), followed by gentamicin (73%) has the highest efficacy for first‐line empirical treatment of *E. coli* (Table 1). These findings differed slightly from two studies where gentamicin was the drug of choice.^13^, ^28^, ^29^

Gram‐positive isolates are most susceptible to first‐line empirical treatment with ampicillin, penicillin or TMS (Table 1). Additionally, Gram‐positive isolates are equally susceptible to penicillin (83%) and ceftiofur (83%), emphasising that ceftiofur should not be used as a routine first‐line antimicrobial. Ampicillin (99%), penicillin (98%) or TMS (91%) have the highest efficacy for first‐line empirical treatment of *S. zooepidemicus* (Table 1). Penicillin was mentioned as the drug of choice in multiple articles for treating infections caused by *S. zooepidemicus* and is often combined with gentamicin for broad‐spectrum coverage.^8^, ^13^, ^14^, ^28^, ^29^

Intrauterine therapy is recommended as the first‐line treatment for endometritis as normal isolate sensitivity testing is based on concentrations expected in blood, not necessarily the uterus with administration of systemic treatment.^14^ Intrauterine therapy is more targeted, and a higher local concentration of antimicrobials can be achieved at the site compared to systemic treatment.^14^ To maximise the efficacy of the antimicrobials and thus reduce the risk of AMR, uterine lavage and ecbolic drugs are suggested in conjunction with intrauterine therapy.^13^, ^14^, ^15^, ^28^, ^29^ Ecbolic drugs to stimulate uterine contractility can also aid in the clearance of uterine fluid.^13^, ^14^, ^28^, ^29^ Systemic treatment, however, is warranted in cases of persistent chronic endometritis or more severe cases like retained placenta and metritis.^14^, ^15^, ^29^ After oestrus and ovulation, the systemic route is the preferred route of administration.^13^, ^14^

### Limitations

The samples provided by the diagnostic laboratory may not be representative of the horse population in WA, as identifying information such as clinical history and referral versus primary accession acquisition was not available. The reason for reproductive tract sampling was unknown and may have been obtained prebreeding, postbreeding or postfoaling. Furthermore, data were analysed from one diagnostic laboratory. However, prior to 2020, due to logistical constraints, most clinical samples were processed by this laboratory. The application of pooled data from multiple clinics/hospitals across WA and a 5‐year analysis period is not conventional for antibiograms according to CLSI guidelines, but has been identified as a way of getting around the challenge of limited isolates for a meaningful interpretation. It was not possible to exclude duplicate isolates from the same horse at different times due to the limited data available.^12^, ^20^ As a result, each horse may not have been equally represented in the antibiogram, which may have led to a falsely increased resistance bias.^17^, ^20^ Culture and sensitivity results are based on *in vitro* effects; therefore, intrauterine and systemic use may not be comparable. Interpretive guidelines for the Kirby‐Bauer test are based on human isolates. CLSI guidelines used in this study are current as of 2007, but may be superseded by more current guidelines, which are not freely available. As per the CLSI guidelines, only species with 30 or more isolates should be included in the antibiogram.^12^, ^17^ However, Klebsiella and Serratia isolates were both still included in the antibiogram despite having only 29 isolates. The inclusion of intermediate susceptibility results as resistant isolates does not allow for species‐specific results or local administration techniques that facilitate drug delivery at increased concentrations to the region affected. Lastly, only a limited number of antimicrobial classes were included in the antibiogram, which limits its ability to identify MDR or extensively drug‐resistant isolates.

## Conclusion

This study describes common reproductive isolates and bacterial sensitivities in mares and fillies from WA. This study has also shown that there is widespread resistance amongst reproductive samples, with increasing resistance to ceftiofur over time. First‐line empirical treatment of Gram‐positive/negative, *E. coli* and *S. zooepidemicus* was provided based on the antibiogram generated, which may mitigate AMR development. Clinicians should be encouraged to submit reproductive tract samples for culture and sensitivity regularly. With the limited number of antimicrobials available, a balance needs to be achieved between broad‐spectrum coverage and ensuring selection of the correct antimicrobial to preserve efficacy long term. As AMR changes over time, antibiograms should be updated to ensure continued accuracy.

## Conflicts of interest and funding

The authors declare no conflicts of interest or sources of funding for the work presented here.

## Supporting information


**Appendix S1.** Due to its length, the entire algorithm will be submitted in a separate document for archiving. A summarised version of the rationale and design features of the code has already been mentioned in the materials and methods section.

## Data Availability

The data that supports the findings of this study are available in the supplementary material of this article.
