# Supplementary material for: Generation of equine‐specific local antibiograms: reproductive tract of mares and fillies in Western Australia
Source: Aust Vet J. 2026 Mar 28;104(7):457–62. doi: 10.1111/avj.70079 (PMC13327455; doi:10.1111/avj.70079)
Supplement: Supplementary file 1 — Appendix S1. Due to its length, the entire algorithm will be submitted in a separate document for archiving. A summarised version of the rationale and design features of the code has already been mentioned in the materials and methods section. [file AVJ-104-457-s001.docx]

Antibiogram.R

Jonathan Hoong

2021-06-19

#This imports our csv file into a variable called antibiogram_data. This variable is a dataframe,
#which is like a table with headings
antibiogram_data <- read.csv("Antibiogram.csv",header=T,stringsAsFactors = T)

#Check all are equine
summary(antibiogram_data$Species)

## EQU
## 2602

#Look at what sample types there are
summary(antibiogram_data$Specimens)

## Uterine swab-J
## 473
## TW H x 1
## 136
## Wound swab-J
## 105
## Trach Wash H x 1
## 68
## Abscess swab-J
## 50
## Nasal swab-J
## 39
## Eye swab-J
## 31
## Vaginal swab-J
## 28
## Premate Swab J x 1
## 23
## Trach. Wash\nTrach Wash
## 23
## Uterine Lavage H x 1
## 23
## Cervical swab-J
## 21
## Uterine Swab J x 1
## 21
## Swab-J x 1
## 20
## Trach. Wash H x 1\nTrach Wash
## 18
## 1 x J premate
## 16
## Tissue H x 1
## 15
## Wound Swab J x 1
## 15
## premate swab J x 1
## 13
## Uterine lavage H x 1
## 13
## Skin swab-J
## 9
## Swab Jx1
## 9
## Fluid H x 1
## 8
## TW H x 1 , add on
## 8
## Urine H x 1
## 8
## Uterine swab-J, Smear Q x 1
## 8
## BAL H x 1
## 7
## Nasal Swab J x 1
## 7
## Trach Wash HB
## 7
## TW in H x 1
## 7
## Uterine flush H x 1
## 7
## Uterine swab-L
## 7
## Blood D x 1 , Blood B x 1 , TW H x 1
## 6
## Body Fluid H x 1
## 6
## premate swabJ
## 6
## Surgical Site Swab J x 1
## 6
## Swab J
## 6
## Swab J x 1
## 6
## Trach. Wash H x 1
## 6
## Uterine Fluid H x 1
## 6
## Blood B x 1 , TW H x 1
## 5
## Clitoral swab x 1
## 5
## fluid h x 1
## 5
## Swab - J
## 5
## trach. Wash H x 1\nTrach Wash
## 5
## TTA H x 1
## 5
## TW H x 2
## 5
## 1 x J uterine lavage
## 4
## 1 x premate J
## 4
## Abscess Swab J x 1
## 4
## BAL LP x 1 , TW H x 1
## 4
## Blood B x 1 , Trach. Wash\nTrach Wash
## 4
## Blood D x 1 , Blood B x 1 , Nasal swab-J
## 4
## castration site swab 1xJ
## 4
## Eye Swab J x 1
## 4
## FNA fluid H x 1
## 4
## Nasal swab J x 1
## 4
## NHW J x 1
## 4
## Surgical site swab J x 1
## 4
## swab J x 1
## 4
## tissue swab J x 1
## 4
## Trach Wash H x 1 , add 5/2
## 4
## Uterine Lavage
## 4
## Uterine swab-K
## 4
## wound swab J x 1
## 4
## 1 x H TW, 1x B TW
## 3
## 1 x J, 1 x H TW
## 3
## 1x Blood cult bottle
## 3
## Bal H x 1
## 3
## BAL LP x 1 , Add 26/09
## 3
## BAL, TW H x 1
## 3
## Blood 6D x 1 , Blood B x 1
## 3
## Blood D x 1 , Blood B x 1 , Fluid H x 1
## 3
## Blood D x 1 , Blood B x 1 , Trach Wash H x 1
## 3
## Blood D x 1 , Blood B x 1 , Trach. Wash\nTrach Wash
## 3
## Blood D,B, Swab Jx1
## 3
## clitoral swab J x 1
## 3
## Clitoral swab J x 1
## 3
## Clitoral swab Jx1
## 3
## Clittoral swab -J
## 3
## Discharge Swab J
## 3
## Dry Swab K x 1
## 3
## Faeces I x 1
## 3
## Fluid B H , Add 18/12
## 3
## Fluid B x 1 , Fluid H x 1 , Fluid LP x 1 , add 29/8
## 3
## Fluid D x 1
## 3
## Fluid H x 2
## 3
## H x 1
## 3
## Histo H x 1 , Tissue H x 1
## 3
## Joint fluid DB x 1
## 3
## Mass Swab J x 2 , Histo H x 1
## 3
## Nasal flush Hx1
## 3
## Nasal discharge H x 1
## 3
## Nasal Fluid H x 1
## 3
## Nasal Swab J x 1, Nasal Discharge 10mL D x 1
## 3
## Nasal Swab Jx1
## 3
## Peritoneal swab-J, Fluid B x 1
## 3
## PM swab J x 1
## 3
## Premate swab- J
## 3
## (Other)
## 1102

#Search for relevant phrases in the sample type and store results in repro_only
repro_only <- antibiogram_data[grepl("uter|Uter|urter|Urter|vag|Vag|endomet|Endomet|clit|Clit|cerv|Cerv|placenta|Placenta|pre|Pre|PM|Pm|pM|pm|permate|Permate|mare|Mare|vulva|Vulva",antibiogram_data$Specimens),]
repro_only$Specimens <- as.factor(as.character(repro_only$Specimens))
repro_only <- repro_only[grepl("penis|shaft",repro_only$Specimens)==F,]
summary(repro_only$Specimens)

## Uterine swab-J
## 473
## Vaginal swab-J
## 28
## Premate Swab J x 1
## 23
## Uterine Lavage H x 1
## 23
## Cervical swab-J
## 21
## Uterine Swab J x 1
## 21
## 1 x J premate
## 16
## premate swab J x 1
## 13
## Uterine lavage H x 1
## 13
## Uterine swab-J, Smear Q x 1
## 8
## Uterine flush H x 1
## 7
## Uterine swab-L
## 7
## premate swabJ
## 6
## Uterine Fluid H x 1
## 6
## Clitoral swab x 1
## 5
## 1 x J uterine lavage
## 4
## 1 x premate J
## 4
## Uterine Lavage
## 4
## Uterine swab-K
## 4
## clitoral swab J x 1
## 3
## Clitoral swab J x 1
## 3
## Clitoral swab Jx1
## 3
## Clittoral swab -J
## 3
## PM swab J x 1
## 3
## Premate swab- J
## 3
## Premate swab J x 1
## 3
## Prematen Swab K x 1, Premate Swab J x 1
## 3
## Premating Swab J x 1
## 3
## Uterine J x 1
## 3
## Uterine Lavage Hx1
## 3
## uterine swab-K
## 3
## Uterine swab J x 1
## 3
## 1 x J uterine
## 2
## 1 x premate swab
## 2
## Cervical Swab J x 1
## 2
## Cervix swab J x 1
## 2
## clitoral swab -J
## 2
## Clitorial swab-K
## 2
## Eq Uterine Swab J x 1
## 2
## Pre-mating Swab Jx 1
## 2
## Premate Clittoral J
## 2
## Premate J x 1
## 2
## Premate swab J
## 2
## Premate swab Jx1
## 2
## Premate Swab pot x 1
## 2
## uterine fluid H x 1
## 2
## uterine Flush H x 1
## 2
## Uterine Flush H x 1
## 2
## Uterine flush H x 1, Uterine swab-J
## 2
## uterine lavage
## 2
## Uterine lavage-J, Agar media-H
## 2
## uterine lavage H x 1
## 2
## Uterine lavage in Hx1
## 2
## Uterine laverage Hx1
## 2
## Uterine swab-J , Hair H x 1 , Uterine Lavage H x 1
## 2
## Uterine swab-J x 1
## 2
## Uterine swab-J, Smear Q x 2
## 2
## Uterine swab-J, Uterine lavage H x 1
## 2
## uterine swab-L
## 2
## Uterine swabs x3-J
## 2
## Uterus lesion swab-J
## 2
## Vag discharge H x 1
## 2
## 1 x H uterine lavage
## 1
## 1 x J endometrium
## 1
## 1 x J uterine biopsy
## 1
## 1 x J uterine swab
## 1
## 1 x Premate
## 1
## 1 x Uterine Container
## 1
## 1x J cliterus
## 1
## 50 ml tube uterine lavage
## 1
## Blood D x 1 , Blood B x 1 , Blood F x 1 , Blood E x 1 , Uterine swab-J
## 1
## Blood D x 1 , Blood B x 1 , Uterine swab-J , Smear Q x 2
## 1
## Blood D x 1, Blood B x 1, Uterine swab-J
## 1
## Cervix J x 1
## 1
## Clit Swab J x 1
## 1
## cliteral swab J x 1
## 1
## Clitoral Dry Swab K x 1
## 1
## Clitoral swab-J
## 1
## Clitoral Swab - J
## 1
## Clitoral Swab J x 1
## 1
## Clitoral swab J x 2, Blood BD x 1
## 1
## culture.plates uterine swab 2 x J
## 1
## Endometrial Swab J x 1 , Histo H x 1
## 1
## EQ PM Swab J pot x 1
## 1
## Eq Premate Swab J
## 1
## Histo H x 1 , Uterine Tissue H x 1
## 1
## Mare Endometritis Swab J x 1
## 1
## Mare Flush H x 1
## 1
## Mare swab J x 1
## 1
## Permate Swab J x 1
## 1
## Placenta Swab Jx 1
## 1
## Pre mate swab Jx1
## 1
## Pre Mating Swab J x 1
## 1
## premate swab-J x 1
## 1
## Premate swab - small pot x1
## 1
## Premate Swab 1xJ`
## 1
## Premate Swab J x 1, Uterine Flush B x 2
## 1
## premate swab Jx1
## 1
## Premate Swab Jx1, Uterine lavage fluid Dx1
## 1
## (Other)
## 62

#Have a look at what was cultured
repro_only$Culture <- as.factor(repro_only$Culture)
summary(repro_only$Culture)

## Non haem E. coli Beta haem. Streptococcus
## 253 171
## Enterococcus sp. Beta haem. Streptococcus#
## 48 37
## Pseudomonas aeruginosa Serratia marcescens
## 28 24
## Enterobacter sp. Klebsiella pneumoniae
## 23 18
## Staphylococcus aureus Haem E.coli
## 12 11
## Enterobacter sp.* Staphylococcus pseudintermedius
## 9 8
## Non haem E. coli* Staphylococcus capitis
## 7 7
## UNIDENTIFIED Acinetobacter lwoffii
## 7 6
## Corynebacterium sp. Pantoea sp.
## 6 6
## Streptococcus uberis Enterobacter aerogenes
## 6 5
## Klebsiella oxytoca Klebsiella pneumoniae*
## 5 5
## Sphingomonas paucimobilis Staphylococcus warneri
## 5 5
## Citrobacter koseri Enterococcus sp.*
## 4 4
## Kocuria rosea Proteus mirabilis
## 4 4
## Staphylococcus schleiferi Acinetobacter lwoffii*
## 4 3
## Enterobacter sp.# Non haem E. coli#
## 3 3
## Staphylococcus haemolyticus* Staphylococcus xylosus
## 3 3
## Streptococcus thoraltensis Acinetobacter sp.
## 3 2
## Actinomyces species Arthrobacter spp.
## 2 2
## Citrobacter koseri* Enterobacter aerogenes*
## 2 2
## Kocuria kristinae* Lactococcus garvieae
## 2 2
## Non haem Streptococcus* Pseudomonas sp.
## 2 2
## Pseudomonas sp.* Pseudomonas stutzeri*
## 2 2
## Serratia liquefaciens Staphylococcus sciuri*
## 2 2
## Staphylococcus xylosus* Streptococcus alactolyticus*
## 2 2
## Streptococcus pseudoporcinus Streptococcus sanguinis
## 2 2
## Achromobacter denitrificans* Achromobacter sp.*
## 1 1
## Achromobacter xylosoxidans Acinetobacter haemolyticus
## 1 1
## Acinetobacter iwoffii Acinetobacter lwoffii#
## 1 1
## Acinetobacter radioresistens Acinetobacter sp.*
## 1 1
## Actinobacillus sp. Actinobacillus suis/equuli
## 1 1
## Aerococcus viridans* Aeromonas media
## 1 1
## Aeromonas sobria Aeromonas sp.*
## 1 1
## Arcanobacterium haemolyticum Arthrobacter spp
## 1 1
## Beta haem. Streptococcus # Beta haem. Streptococcus*
## 1 1
## Bordetella bronchiseptica Bordetella bronchiseptica*
## 1 1
## Bordetella sp. Brevibacterium spp. ##
## 1 1
## Candida parasilosis# Cellulomonas spp/Microbacterium spp
## 1 1
## Cellulomonas spp/Microbacterium spp. Cellulomonas spp/Microbacterium spp.*
## 1 1
## Citrobacter amalonaticus Citrobacter freundii
## 1 1
## Citrobacter freundii# Citrobacter sp.
## 1 1
## Coagulase negative Staphylococcus sp. Comamonas testosteroni A*
## 1 1
## Corynebacterium glucuronolyticum Corynebacterium propinquum
## 1 1
## Corynebacterium sp.* Corynebacterium urealyticum
## 1 1
## Enterococcus casseliflavus Enterococcus faecalis
## 1 1
## Enterococcus sp. (beta haemolytic) Environmental contaminant
## 1 1
## Escherichia hermannii Escherichia hermannii*
## 1 1
## Escherichia vulneris Ewingella americana
## 1 1
## Klebsiella oxytoca* Kluyvera intermedia*
## 1 1
## Kocuria kristinae (Other)
## 1 53

#Make groups for all strep, staph, E. coli, pseudomonas, serratia, enterococcus, enterobacter, acinetobacter, klebsiella
strep <- repro_only[grepl("Strep|strep",repro_only$Culture),]
strep$Culture <- as.factor(as.character(strep$Culture))
strep$Specimens <- as.factor(as.character(strep$Specimens))
summary(strep$Culture)

## Beta haem. Streptococcus
## 171
## Beta haem. Streptococcus #
## 1
## Beta haem. Streptococcus#
## 37
## Beta haem. Streptococcus*
## 1
## Non haem Streptococcus
## 1
## Non haem Streptococcus#
## 1
## Non haem Streptococcus*
## 2
## Streptococcus alactolyticus
## 1
## Streptococcus alactolyticus*
## 2
## Streptococcus dysagalactiae ssp equisimilis
## 1
## Streptococcus equi ssp zooepidemicus
## 1
## Streptococcus gallolyticus ssp gallolyticus
## 1
## Streptococcus ovis
## 1
## Streptococcus pluranimalium#
## 1
## Streptococcus pseudoporcinus
## 2
## Streptococcus sanguinis
## 2
## Streptococcus sp.
## 1
## Streptococcus spp
## 1
## Streptococcus suis
## 1
## Streptococcus thoraltensis
## 3
## Streptococcus thoraltensis*
## 1
## Streptococcus uberis
## 6
## Streptococcus uberis*
## 1

summary(strep$Specimens)

## 1 x J premate
## 6
## 1 x J uterine swab
## 1
## 1 x premate J
## 1
## Blood D x 1, Blood B x 1, Uterine swab-J
## 1
## Cervical swab-J
## 7
## Cervical Swab J x 1
## 1
## Cervix swab J x 1
## 1
## clitoral swab J x 1
## 1
## Clitoral swab J x 2, Blood BD x 1
## 1
## Clitoral swab Jx1
## 2
## Clittoral swab -J
## 1
## Mare swab J x 1
## 1
## Pre Mating Swab J x 1
## 1
## Premate swab- J
## 1
## premate swab-J x 1
## 1
## premate swab J x 1
## 4
## Premate swab J x 1
## 2
## Premate Swab J x 1
## 10
## premate swab Jx1
## 1
## Premate Swab Jx1, Uterine lavage fluid Dx1
## 1
## premate swabJ
## 1
## Premating Swab J x 1
## 1
## Swab mare x1 J
## 1
## Urine H x 1 , Uterine swab-J
## 1
## Uterine fluid H
## 1
## uterine fluid H x 1
## 1
## Uterine Fluid H x 1
## 2
## Uterine fluid in falcon tube
## 1
## uterine fluid tube x 2 , add 29/11
## 1
## Uterine Flush-H x 1
## 1
## Uterine flush H x 1
## 2
## Uterine Flush H x 1
## 1
## uterine flush in H x 1
## 1
## Uterine J x 1
## 1
## Uterine larvge-J
## 1
## Uterine lavage
## 1
## Uterine Lavage
## 1
## Uterine Lavage 2 x 50ml Tubes
## 1
## Uterine Lavage 50ml x 2
## 1
## uterine lavage fluid H x 1
## 1
## uterine lavage H x 1
## 1
## Uterine lavage H x 1
## 4
## Uterine Lavage H x 1
## 8
## Uterine lavage H x 1, add 13/12
## 1
## Uterine Lavage H x 2
## 1
## Uterine Lavage Hx 1
## 1
## Uterine lavage Hx1
## 1
## Uterine Lavage Hx1
## 2
## Uterine Lavage in 50mL Tube x 1
## 1
## Uterine laverage Hx1
## 1
## Uterine swab- J
## 1
## Uterine swab-J
## 121
## Uterine swab-J , Hair H x 1 , Uterine Lavage H x 1
## 1
## Uterine swab-J , Smear Q x 2 , add 18/1
## 1
## uterine swab-J x 1
## 1
## Uterine swab-J, Smear Q x 1
## 2
## Uterine swab-J, Smear Q x 2
## 1
## Uterine swab-J, Uterine lavage H x 1
## 1
## Uterine swab-JQ
## 1
## Uterine swab-L
## 2
## uterine swab - K x 1
## 1
## Uterine swab J x 1, Smear Q x 2 , add 6/12
## 1
## Uterine swab J x 1
## 1
## Uterine Swab J x 1
## 7
## Uterus swab-J
## 1
## Uterus swab-J x 1
## 1
## Vag discharge H x 1
## 1
## Vaginal swab-J
## 6
## Vulval Discharge Swab J x 1
## 1

summary(strep)

## ï..Job_Number ClientCode JobDate Species PostCode
## Min. :403926 Min. : 3.0 18/10/2016: 3 EQU:240 Min. :6055
## 1st Qu.:491475 1st Qu.: 149.0 19/11/2016: 3 1st Qu.:6121
## Median :584165 Median : 308.0 20/12/2016: 3 Median :6167
## Mean :585232 Mean : 530.5 1/11/2018 : 2 Mean :6217
## 3rd Qu.:682239 3rd Qu.: 975.0 10/1/2018 : 2 3rd Qu.:6231
## Max. :798855 Max. :1240.0 12/3/2019 : 2 Max. :6935
## (Other) :225
## Specimens Prefix Culture
## Uterine swab-J :121 F : 0 Beta haem. Streptococcus :171
## Premate Swab J x 1 : 10 M1:240 Beta haem. Streptococcus# : 37
## Uterine Lavage H x 1: 8 U : 0 Streptococcus uberis : 6
## Cervical swab-J : 7 Streptococcus thoraltensis : 3
## Uterine Swab J x 1 : 7 Non haem Streptococcus* : 2
## 1 x J premate : 6 Streptococcus alactolyticus*: 2
## (Other) : 81 (Other) : 19
## Amikacin Amoxycillin Ampicillin Bactracin Carbenicillin Ceftazidime Ceftiofur
## :240 :238 : 2 :240 :240 :240 : 0
## R: 0 I: 0 R: 3 S: 0 R: 0 R: 0 R: 1
## S: 0 R: 0 S:235 S: 0 S: 0 S:239
## X: 0 S: 2 X: 0 X: 0 X: 0 X: 0
## X: 0
##
##
## Cefuroxime Cephalexin Chloramphenicol Ciprofloxacin Clavulox Clavulox_Clavulox
## :240 :240 :240 :240 :240 :240
## S: 0 R: 0 R: 0 I: 0 R: 0 S: 0
## S: 0 S: 0 R: 0 S: 0
## X: 0 X: 0 S: 0 X: 0
##
##
##
## Clindamycin Cloxacillin Doxcycline Doxycycline Doxyycline Enrofloxacin
## :240 :240 :240 :240 :240 : 2
## R: 0 R: 0 R: 0 I: 0 S: 0 I:167
## S: 0 S: 0 S: 0 R: 0 R: 22
## X: 0 X: 0 S: 0 S: 49
## X: 0
##
##
## Enrofloxaxin Erythromycin Fusidic_Acid Gentamicin Lincomycin Marbofloxacin
## :240 :238 :240 : 0 :240 :240
## R: 0 R: 0 R: 0 I: 1 R: 0 I: 0
## S: 2 S: 0 R: 9 S: 0 R: 0
## X: 0 X: 0 S: 1 S: 0
## X:229
##
##
## Minocycline Mupirocin Neomycin Norfloxacin Novobiocin Ofloxacin Penicillin
## :240 :240 : 2 :240 :240 :240 :238
## S: 0 R: 0 I: 0 R: 0 R: 0 R: 0 R: 0
## S: 0 R: 10 S: 0 S: 0 S: 0 S: 2
## S: 1 X: 0
## X:227
##
##
## Penicillin_G Pipercillin Polymyxin_B Rifampicin Soframycin Streptomycin
## : 2 :240 :240 :240 :240 Mode:logical
## R: 5 S: 0 R: 0 R: 0 R: 0 NA's:240
## S:233 S: 0 S: 0 S: 0
## X: 0 X: 0 X: 0 X: 0
##
##
##
## Sulphatrimethoprim Tetracycline Ticarcillin Timentin TestDate
## : 0 : 0 :240 :148 Mode:logical
## R: 21 I: 0 R: 0 I: 0 NA's:240
## S:218 R:194 S: 0 R: 1
## X: 1 S: 46 X: 0 S: 91
## X: 0 X: 0
##
##
## ULeucocytes UEpithelial URBC UCasts
## :238 :238 :238 :238
## <1 : 1 <1 : 1 <1 : 1 None seen: 2
## 29 : 1 1 : 1 >200: 0
## >200 : 0 18 : 0 1 : 1
## 135 : 0 2 : 0 116 : 0
## 2 : 0 36 : 0 3 : 0
## (Other): 0 (Other): 0
## UCrystals UBacteria USpermatoza
## :238 :239 Mode:logical
## Calcium oxalate 3+ : 1 1+ : 0 NA's:240
## None seen : 1 3+ : 0
## 1+ Ca carbonate : 0 Few : 1
## 3+ Ca carbonate, Occ Ca oxalate: 0 Occasional: 0
## 3+ calcium carbonate : 0
## (Other) : 0
## UFungal UDebris UGram USG UPh
## :240 :240 Mode:logical Min. :1.000 :238
## 2+ Hyphae: 0 1+: 0 NA's:240 1st Qu.:1.001 >=9.0: 0
## 3+: 0 Median :1.002 6 : 1
## Mean :1.002 8 : 1
## 3rd Qu.:1.004 8.5 : 0
## Max. :1.005 9 : 0
## NA's :238
## UProtein UGlucose UKetones UBlood UBilirubin
## :239 :239 :239 :239 :239
## 1+ : 0 3+ : 0 1+ : 0 1+ : 0 1+ : 0
## 2+ : 0 Negative: 1 Negative: 1 3+ : 0 3+ : 0
## 3+ : 0 Trace : 0 Trace : 0 Negative: 1 Negative: 1
## Negative: 1
##
##
## UCultureNote M1Leucocytes M1RBC M1Epithelial
## :239 :240 :240 :240
## TO FOLLOW : 1 1+ : 0 1+ : 0 1+ : 0
## >10,000 CFU/mL : 0 2+ : 0 2+ : 0 2+ : 0
## >100,000 CFU/mL: 0 3+ : 0 3+ : 0 3+ : 0
## 2,000 CFU/mL : 0 FEW : 0 FEW : 0 FEW : 0
## 3,200 CFU/mL : 0 NONE SEEN: 0 NONE SEEN: 0 NONE SEEN: 0
## (Other) : 0 (Other) : 0 (Other) : 0 (Other) : 0
## M1Fungal FMicroForExcess FLeucocytes
## :240 Mode:logical :240
## 2+ fungal hyphae : 0 NA's:240 None seen: 0
## 2+ Yeast cells and Fungal Hyphae: 0
## FEW * : 0
## FEW FUNGAL HYPHAE : 0
## FUNGAL HYPHAE 1+ : 0
## (Other) : 0
## FRBC FCampylobacter FYeast FProtozoaCystsOva
## :240 :240 :240 :240
## None seen: 0 None seen: 0 None seen : 0 None seen: 0
## Very occasional: 0
##
##
##
##
## FTrypsin10 FTrypsin100 FOccultBlood
## Mode:logical Mode:logical Mode:logical
## NA's:240 NA's:240 NA's:240
##
##
##
##
##

staph <- repro_only[grepl("Staph|staph",repro_only$Culture),]
staph$Culture <- as.factor(as.character(staph$Culture))
staph$Specimens <- as.factor(as.character(staph$Specimens))
summary(staph$Culture)

## Coagulase negative Staphylococcus sp. Staphylococcus aureus
## 1 12
## Staphylococcus capitis Staphylococcus chromogenes
## 7 1
## Staphylococcus cohnii Staphylococcus epidermidis
## 1 1
## Staphylococcus haemolyticus Staphylococcus haemolyticus*
## 1 3
## Staphylococcus lentus Staphylococcus pseudintermedius
## 1 8
## Staphylococcus pseudintermedius* Staphylococcus schleiferi
## 1 4
## Staphylococcus sciuri Staphylococcus sciuri*
## 1 2
## Staphylococcus sp.* Staphylococcus warneri
## 1 5
## Staphylococcus warneri* Staphylococcus xylosus
## 1 3
## Staphylococcus xylosus*
## 2

summary(staph$Specimens)

## 1 x J endometrium
## 1
## 1 x J uterine lavage
## 1
## 1 x premate J
## 1
## Blood D x 1 , Blood B x 1 , Blood F x 1 , Blood E x 1 , Uterine swab-J
## 1
## Clit Swab J x 1
## 1
## Clitoral Dry Swab K x 1
## 1
## Clittoral swab -J
## 1
## Permate Swab J x 1
## 1
## premate swab J x 1
## 1
## Premate Swab J x 1
## 1
## Premate swab Jx1
## 1
## Uterine J x 1
## 1
## Uterine Lavage
## 1
## Uterine lavage H x 1
## 1
## Uterine lavage in Hx1
## 1
## Uterine swab-J
## 29
## Uterine swab-J x 1
## 1
## Uterine swab-J, Smear Q x 1
## 2
## Uterine swab-K
## 1
## Uterine swab L x 1
## 1
## Uterine swabs x3-J
## 1
## Vaginal swab-J
## 6

summary(staph)

## ï..Job_Number ClientCode JobDate Species PostCode
## Min. :410584 Min. : 19.0 20/10/2015: 3 EQU:56 Min. :6055
## 1st Qu.:497437 1st Qu.: 101.0 21/9/2018 : 2 1st Qu.:6121
## Median :590590 Median : 234.5 1/9/2018 : 1 Median :6171
## Mean :595590 Mean : 487.5 10/1/2020 : 1 Mean :6190
## 3rd Qu.:679821 3rd Qu.:1025.0 10/9/2018 : 1 3rd Qu.:6225
## Max. :776201 Max. :1240.0 12/9/2017 : 1 Max. :6751
## (Other) :47
## Specimens Prefix Culture
## Uterine swab-J :29 F : 0 Staphylococcus aureus :12
## Vaginal swab-J : 6 M1:56 Staphylococcus pseudintermedius: 8
## Uterine swab-J, Smear Q x 1: 2 U : 0 Staphylococcus capitis : 7
## 1 x J endometrium : 1 Staphylococcus warneri : 5
## 1 x J uterine lavage : 1 Staphylococcus schleiferi : 4
## 1 x premate J : 1 Staphylococcus haemolyticus* : 3
## (Other) :16 (Other) :17
## Amikacin Amoxycillin Ampicillin Bactracin Carbenicillin Ceftazidime Ceftiofur
## :56 :54 : 2 :56 :56 :56 : 1
## R: 0 I: 0 R:40 S: 0 R: 0 R: 0 R: 3
## S: 0 R: 2 S:14 S: 0 S: 0 S:52
## X: 0 S: 0 X: 0 X: 0 X: 0 X: 0
## X: 0
##
##
## Cefuroxime Cephalexin Chloramphenicol Ciprofloxacin Clavulox Clavulox_Clavulox
## :56 :55 :56 :56 :55 :56
## S: 0 R: 0 R: 0 I: 0 R: 0 S: 0
## S: 1 S: 0 R: 0 S: 1
## X: 0 X: 0 S: 0 X: 0
##
##
##
## Clindamycin Cloxacillin Doxcycline Doxycycline Doxyycline Enrofloxacin
## :56 :56 :56 :55 :56 : 2
## R: 0 R: 0 R: 0 I: 0 S: 0 I: 0
## S: 0 S: 0 S: 0 R: 0 R: 2
## X: 0 X: 0 S: 1 S:52
## X: 0
##
##
## Enrofloxaxin Erythromycin Fusidic_Acid Gentamicin Lincomycin Marbofloxacin
## :56 :54 :56 : 0 :56 :55
## R: 0 R: 1 R: 0 I: 0 R: 0 I: 0
## S: 1 S: 0 R:10 S: 0 R: 0
## X: 0 X: 0 S:46 S: 1
## X: 0
##
##
## Minocycline Mupirocin Neomycin Norfloxacin Novobiocin Ofloxacin Penicillin
## :56 :56 : 1 :56 :56 :56 :54
## S: 0 R: 0 I: 0 R: 0 R: 0 R: 0 R: 2
## S: 0 R: 9 S: 0 S: 0 S: 0 S: 0
## S:46 X: 0
## X: 0
##
##
## Penicillin_G Pipercillin Polymyxin_B Rifampicin Soframycin Streptomycin
## : 3 :56 :56 :56 :56 Mode:logical
## R:38 S: 0 R: 0 R: 0 R: 0 NA's:56
## S:15 S: 0 S: 0 S: 0
## X: 0 X: 0 X: 0 X: 0
##
##
##
## Sulphatrimethoprim Tetracycline Ticarcillin Timentin TestDate
## : 0 : 1 :56 :38 Mode:logical
## R: 7 I: 0 R: 0 I: 1 NA's:56
## S:49 R:14 S: 0 R: 2
## X: 0 S:41 X: 0 S:15
## X: 0 X: 0
##
##
## ULeucocytes UEpithelial URBC UCasts
## :56 :56 :56 :56
## <1 : 0 <1 : 0 <1 : 0 None seen: 0
## >200 : 0 1 : 0 >200: 0
## 135 : 0 18 : 0 1 : 0
## 2 : 0 2 : 0 116 : 0
## 29 : 0 36 : 0 3 : 0
## (Other): 0 (Other): 0
## UCrystals UBacteria USpermatoza
## :56 :56 Mode:logical
## 1+ Ca carbonate : 0 1+ : 0 NA's:56
## 3+ Ca carbonate, Occ Ca oxalate : 0 3+ : 0
## 3+ calcium carbonate : 0 Few : 0
## 3+ Calcium carbonate, few calcium oxalate: 0 Occasional: 0
## Ca carbonate 3+ : 0
## (Other) : 0
## UFungal UDebris UGram USG UPh UProtein
## :56 :56 Mode:logical Min. : NA :56 :56
## 2+ Hyphae: 0 1+: 0 NA's:56 1st Qu.: NA >=9.0: 0 1+ : 0
## 3+: 0 Median : NA 6 : 0 2+ : 0
## Mean :NaN 8 : 0 3+ : 0
## 3rd Qu.: NA 8.5 : 0 Negative: 0
## Max. : NA 9 : 0
## NA's :56
## UGlucose UKetones UBlood UBilirubin UCultureNote
## :56 :56 :56 :56 :56
## 3+ : 0 1+ : 0 1+ : 0 1+ : 0 >10,000 CFU/mL : 0
## Negative: 0 Negative: 0 3+ : 0 3+ : 0 >100,000 CFU/mL: 0
## Trace : 0 Trace : 0 Negative: 0 Negative: 0 2,000 CFU/mL : 0
## 3,200 CFU/mL : 0
## 4,000 CFU/mL : 0
## (Other) : 0
## M1Leucocytes M1RBC M1Epithelial
## :56 :56 :56
## 1+ : 0 1+ : 0 1+ : 0
## 2+ : 0 2+ : 0 2+ : 0
## 3+ : 0 3+ : 0 3+ : 0
## FEW : 0 FEW : 0 FEW : 0
## NONE SEEN: 0 NONE SEEN: 0 NONE SEEN: 0
## (Other) : 0 (Other) : 0 (Other) : 0
## M1Fungal FMicroForExcess FLeucocytes
## :56 Mode:logical :56
## 2+ fungal hyphae : 0 NA's:56 None seen: 0
## 2+ Yeast cells and Fungal Hyphae: 0
## FEW * : 0
## FEW FUNGAL HYPHAE : 0
## FUNGAL HYPHAE 1+ : 0
## (Other) : 0
## FRBC FCampylobacter FYeast FProtozoaCystsOva
## :56 :56 :56 :56
## None seen: 0 None seen: 0 None seen : 0 None seen: 0
## Very occasional: 0
##
##
##
##
## FTrypsin10 FTrypsin100 FOccultBlood
## Mode:logical Mode:logical Mode:logical
## NA's:56 NA's:56 NA's:56
##
##
##
##
##

serratia <- repro_only[grepl("Serratia|serratia",repro_only$Culture),]
serratia$Culture <- as.factor(as.character(serratia$Culture))
serratia$Specimens <- as.factor(as.character(serratia$Specimens))
summary(serratia$Culture)

## Serratia fonticola Serratia liquefaciens Serratia marcescens
## 1 2 24
## Serratia marcescens# Serratia sp.
## 1 1

summary(serratia$Specimens)

## 1 x J premate Cervical swab-J
## 2 1
## Premate swab J Premate Swab J x 1, Uterine Flush B x 2
## 1 1
## Premate Swab L x 1 premate swabJ
## 1 1
## Uterine Flush H pot x 1 uterine Flush H x 1
## 1 2
## Uterine Lavage H x 1 uterine lavage x 1
## 1 1
## Uterine swab-J Uterine Swab 1xJ
## 15 1
## Vaginal swab-J
## 1

summary(serratia)

## ï..Job_Number ClientCode JobDate Species PostCode
## Min. :401563 Min. : 23.0 4/11/2017 : 3 EQU:29 Min. :6055
## 1st Qu.:431566 1st Qu.: 226.0 3/11/2016 : 2 1st Qu.:6055
## Median :503938 Median : 975.0 1/12/2016 : 1 Median :6069
## Mean :508978 Mean : 763.8 1/3/2017 : 1 Mean :6157
## 3rd Qu.:560721 3rd Qu.: 975.0 10/5/2019 : 1 3rd Qu.:6171
## Max. :749313 Max. :1240.0 11/10/2016: 1 Max. :6525
## (Other) :20
## Specimens Prefix
## Uterine swab-J :15 F : 0
## 1 x J premate : 2 M1:29
## uterine Flush H x 1 : 2 U : 0
## Cervical swab-J : 1
## Premate swab J : 1
## Premate Swab J x 1, Uterine Flush B x 2: 1
## (Other) : 7
## Culture Amikacin Amoxycillin Ampicillin Bactracin
## Serratia fonticola : 1 :29 :28 : 1 :29
## Serratia liquefaciens: 2 R: 0 I: 0 R: 0 S: 0
## Serratia marcescens :24 S: 0 R: 0 S: 0
## Serratia marcescens# : 1 X: 0 S: 0 X:28
## Serratia sp. : 1 X: 1
##
##
## Carbenicillin Ceftazidime Ceftiofur Cefuroxime Cephalexin Chloramphenicol
## :29 :29 : 0 :29 :29 :29
## R: 0 R: 0 R: 3 S: 0 R: 0 R: 0
## S: 0 S: 0 S:26 S: 0 S: 0
## X: 0 X: 0 X: 0 X: 0 X: 0
##
##
##
## Ciprofloxacin Clavulox Clavulox_Clavulox Clindamycin Cloxacillin Doxcycline
## :29 :29 :29 :29 :29 :29
## I: 0 R: 0 S: 0 R: 0 R: 0 R: 0
## R: 0 S: 0 S: 0 S: 0 S: 0
## S: 0 X: 0 X: 0 X: 0
##
##
##
## Doxycycline Doxyycline Enrofloxacin Enrofloxaxin Erythromycin Fusidic_Acid
## :29 :29 : 1 :29 :28 :29
## I: 0 S: 0 I: 9 R: 0 R: 0 R: 0
## R: 0 R: 0 S: 0 S: 0
## S: 0 S:19 X: 1 X: 0
## X: 0
##
##
## Gentamicin Lincomycin Marbofloxacin Minocycline Mupirocin Neomycin Norfloxacin
## : 0 :29 :29 :29 :29 : 0 :29
## I: 0 R: 0 I: 0 S: 0 R: 0 I: 0 R: 0
## R: 1 S: 0 R: 0 S: 0 R: 8 S: 0
## S:28 S: 0 S:21
## X: 0 X: 0
##
##
## Novobiocin Ofloxacin Penicillin Penicillin_G Pipercillin Polymyxin_B
## :29 :29 :28 : 1 :29 :29
## R: 0 R: 0 R: 0 R:27 S: 0 R: 0
## S: 0 S: 0 S: 0 S: 1 S: 0
## X: 1 X: 0 X: 0
##
##
##
## Rifampicin Soframycin Streptomycin Sulphatrimethoprim Tetracycline
## :29 :29 Mode:logical : 0 : 0
## R: 0 R: 0 NA's:29 R: 0 I: 0
## S: 0 S: 0 S:29 R:26
## X: 0 X: 0 X: 0 S: 3
## X: 0
##
##
## Ticarcillin Timentin TestDate ULeucocytes UEpithelial URBC
## :29 :11 Mode:logical :29 :29 :29
## R: 0 I: 0 NA's:29 <1 : 0 <1 : 0 <1 : 0
## S: 0 R: 1 >200 : 0 1 : 0 >200: 0
## X: 0 S:17 135 : 0 18 : 0 1 : 0
## X: 0 2 : 0 2 : 0 116 : 0
## 29 : 0 36 : 0 3 : 0
## (Other): 0 (Other): 0
## UCasts UCrystals UBacteria
## :29 :29 :29
## None seen: 0 1+ Ca carbonate : 0 1+ : 0
## 3+ Ca carbonate, Occ Ca oxalate : 0 3+ : 0
## 3+ calcium carbonate : 0 Few : 0
## 3+ Calcium carbonate, few calcium oxalate: 0 Occasional: 0
## Ca carbonate 3+ : 0
## (Other) : 0
## USpermatoza UFungal UDebris UGram USG UPh
## Mode:logical :29 :29 Mode:logical Min. : NA :29
## NA's:29 2+ Hyphae: 0 1+: 0 NA's:29 1st Qu.: NA >=9.0: 0
## 3+: 0 Median : NA 6 : 0
## Mean :NaN 8 : 0
## 3rd Qu.: NA 8.5 : 0
## Max. : NA 9 : 0
## NA's :29
## UProtein UGlucose UKetones UBlood UBilirubin
## :29 :29 :29 :29 :29
## 1+ : 0 3+ : 0 1+ : 0 1+ : 0 1+ : 0
## 2+ : 0 Negative: 0 Negative: 0 3+ : 0 3+ : 0
## 3+ : 0 Trace : 0 Trace : 0 Negative: 0 Negative: 0
## Negative: 0
##
##
## UCultureNote M1Leucocytes M1RBC M1Epithelial
## :29 :29 :29 :29
## >10,000 CFU/mL : 0 1+ : 0 1+ : 0 1+ : 0
## >100,000 CFU/mL: 0 2+ : 0 2+ : 0 2+ : 0
## 2,000 CFU/mL : 0 3+ : 0 3+ : 0 3+ : 0
## 3,200 CFU/mL : 0 FEW : 0 FEW : 0 FEW : 0
## 4,000 CFU/mL : 0 NONE SEEN: 0 NONE SEEN: 0 NONE SEEN: 0
## (Other) : 0 (Other) : 0 (Other) : 0 (Other) : 0
## M1Fungal FMicroForExcess FLeucocytes
## :29 Mode:logical :29
## 2+ fungal hyphae : 0 NA's:29 None seen: 0
## 2+ Yeast cells and Fungal Hyphae: 0
## FEW * : 0
## FEW FUNGAL HYPHAE : 0
## FUNGAL HYPHAE 1+ : 0
## (Other) : 0
## FRBC FCampylobacter FYeast FProtozoaCystsOva
## :29 :29 :29 :29
## None seen: 0 None seen: 0 None seen : 0 None seen: 0
## Very occasional: 0
##
##
##
##
## FTrypsin10 FTrypsin100 FOccultBlood
## Mode:logical Mode:logical Mode:logical
## NA's:29 NA's:29 NA's:29
##
##
##
##
##

acinetobacter <- repro_only[grepl("Acinetobacter|acinetobacter",repro_only$Culture),]
acinetobacter$Culture <- as.factor(as.character(acinetobacter$Culture))
acinetobacter$Specimens <- as.factor(as.character(acinetobacter$Specimens))
summary(acinetobacter$Culture)

## Acinetobacter haemolyticus Acinetobacter iwoffii
## 1 1
## Acinetobacter lwoffii Acinetobacter lwoffii#
## 6 1
## Acinetobacter lwoffii* Acinetobacter radioresistens
## 3 1
## Acinetobacter sp. Acinetobacter sp.*
## 2 1

summary(acinetobacter$Specimens)

## 1 x J premate Premate Clittoral J
## 1 1
## premate swab J x 1 Premate Swab J x 1
## 1 1
## Uterine flush H x 1 uterine lavage
## 1 1
## Uterine swab-J Uterine swab-J, Smear Q x 1
## 7 1
## uterine swab-L Vaginal swab-J
## 1 1

summary(acinetobacter)

## ï..Job_Number ClientCode JobDate Species PostCode
## Min. :412495 Min. : 101.0 1/12/2016 : 1 EQU:16 Min. :6084
## 1st Qu.:494715 1st Qu.: 292.2 1/9/2018 : 1 1st Qu.:6163
## Median :551347 Median : 720.0 11/11/2016: 1 Median :6193
## Mean :576765 Mean : 692.0 12/1/2017 : 1 Mean :6277
## 3rd Qu.:661706 3rd Qu.:1051.5 13/10/2015: 1 3rd Qu.:6299
## Max. :761751 Max. :1240.0 15/11/2018: 1 Max. :6751
## (Other) :10
## Specimens Prefix Culture Amikacin
## Uterine swab-J :7 F : 0 Acinetobacter lwoffii :6 :16
## 1 x J premate :1 M1:16 Acinetobacter lwoffii* :3 R: 0
## Premate Clittoral J:1 U : 0 Acinetobacter sp. :2 S: 0
## premate swab J x 1 :1 Acinetobacter haemolyticus:1 X: 0
## Premate Swab J x 1 :1 Acinetobacter iwoffii :1
## Uterine flush H x 1:1 Acinetobacter lwoffii# :1
## (Other) :4 (Other) :2
## Amoxycillin Ampicillin Bactracin Carbenicillin Ceftazidime Ceftiofur
## :16 :0 :16 :16 :16 : 0
## I: 0 R:6 S: 0 R: 0 R: 0 R:11
## R: 0 S:9 S: 0 S: 0 S: 5
## S: 0 X:1 X: 0 X: 0 X: 0
## X: 0
##
##
## Cefuroxime Cephalexin Chloramphenicol Ciprofloxacin Clavulox Clavulox_Clavulox
## :16 :16 :16 :16 :16 :16
## S: 0 R: 0 R: 0 I: 0 R: 0 S: 0
## S: 0 S: 0 R: 0 S: 0
## X: 0 X: 0 S: 0 X: 0
##
##
##
## Clindamycin Cloxacillin Doxcycline Doxycycline Doxyycline Enrofloxacin
## :16 :16 :16 :16 :16 : 0
## R: 0 R: 0 R: 0 I: 0 S: 0 I: 2
## S: 0 S: 0 S: 0 R: 0 R: 1
## X: 0 X: 0 S: 0 S:13
## X: 0
##
##
## Enrofloxaxin Erythromycin Fusidic_Acid Gentamicin Lincomycin Marbofloxacin
## :16 :16 :16 : 0 :16 :16
## R: 0 R: 0 R: 0 I: 0 R: 0 I: 0
## S: 0 S: 0 R: 3 S: 0 R: 0
## X: 0 X: 0 S:13 S: 0
## X: 0
##
##
## Minocycline Mupirocin Neomycin Norfloxacin Novobiocin Ofloxacin Penicillin
## :16 :16 : 0 :16 :16 :16 :16
## S: 0 R: 0 I: 0 R: 0 R: 0 R: 0 R: 0
## S: 0 R: 2 S: 0 S: 0 S: 0 S: 0
## S:14 X: 0
## X: 0
##
##
## Penicillin_G Pipercillin Polymyxin_B Rifampicin Soframycin Streptomycin
## : 0 :16 :16 :16 :16 Mode:logical
## R:15 S: 0 R: 0 R: 0 R: 0 NA's:16
## S: 1 S: 0 S: 0 S: 0
## X: 0 X: 0 X: 0 X: 0
##
##
##
## Sulphatrimethoprim Tetracycline Ticarcillin Timentin TestDate
## : 0 : 0 :16 :9 Mode:logical
## R:10 I: 0 R: 0 I:0 NA's:16
## S: 6 R: 4 S: 0 R:3
## X: 0 S:12 X: 0 S:4
## X: 0 X:0
##
##
## ULeucocytes UEpithelial URBC UCasts
## :16 :16 :16 :16
## <1 : 0 <1 : 0 <1 : 0 None seen: 0
## >200 : 0 1 : 0 >200: 0
## 135 : 0 18 : 0 1 : 0
## 2 : 0 2 : 0 116 : 0
## 29 : 0 36 : 0 3 : 0
## (Other): 0 (Other): 0
## UCrystals UBacteria USpermatoza
## :16 :16 Mode:logical
## 1+ Ca carbonate : 0 1+ : 0 NA's:16
## 3+ Ca carbonate, Occ Ca oxalate : 0 3+ : 0
## 3+ calcium carbonate : 0 Few : 0
## 3+ Calcium carbonate, few calcium oxalate: 0 Occasional: 0
## Ca carbonate 3+ : 0
## (Other) : 0
## UFungal UDebris UGram USG UPh UProtein
## :16 :16 Mode:logical Min. : NA :16 :16
## 2+ Hyphae: 0 1+: 0 NA's:16 1st Qu.: NA >=9.0: 0 1+ : 0
## 3+: 0 Median : NA 6 : 0 2+ : 0
## Mean :NaN 8 : 0 3+ : 0
## 3rd Qu.: NA 8.5 : 0 Negative: 0
## Max. : NA 9 : 0
## NA's :16
## UGlucose UKetones UBlood UBilirubin UCultureNote
## :16 :16 :16 :16 :16
## 3+ : 0 1+ : 0 1+ : 0 1+ : 0 >10,000 CFU/mL : 0
## Negative: 0 Negative: 0 3+ : 0 3+ : 0 >100,000 CFU/mL: 0
## Trace : 0 Trace : 0 Negative: 0 Negative: 0 2,000 CFU/mL : 0
## 3,200 CFU/mL : 0
## 4,000 CFU/mL : 0
## (Other) : 0
## M1Leucocytes M1RBC M1Epithelial
## :16 :16 :16
## 1+ : 0 1+ : 0 1+ : 0
## 2+ : 0 2+ : 0 2+ : 0
## 3+ : 0 3+ : 0 3+ : 0
## FEW : 0 FEW : 0 FEW : 0
## NONE SEEN: 0 NONE SEEN: 0 NONE SEEN: 0
## (Other) : 0 (Other) : 0 (Other) : 0
## M1Fungal FMicroForExcess FLeucocytes
## :16 Mode:logical :16
## 2+ fungal hyphae : 0 NA's:16 None seen: 0
## 2+ Yeast cells and Fungal Hyphae: 0
## FEW * : 0
## FEW FUNGAL HYPHAE : 0
## FUNGAL HYPHAE 1+ : 0
## (Other) : 0
## FRBC FCampylobacter FYeast FProtozoaCystsOva
## :16 :16 :16 :16
## None seen: 0 None seen: 0 None seen : 0 None seen: 0
## Very occasional: 0
##
##
##
##
## FTrypsin10 FTrypsin100 FOccultBlood
## Mode:logical Mode:logical Mode:logical
## NA's:16 NA's:16 NA's:16
##
##
##
##
##

e.coli <- repro_only[grepl("E.coli|e.coli|E. coli|e. coli",repro_only$Culture),]
e.coli$Culture <- as.factor(as.character(e.coli$Culture))
e.coli$Specimens <- as.factor(as.character(e.coli$Specimens))
summary(e.coli$Culture)

## Haem E.coli Non haem E. coli Non haem E. coli (mucoid)
## 11 253 1
## Non haem E. coli A Non haem E. coli B* Non haem E. coli#
## 1 1 3
## Non haem E. coli*
## 7

summary(e.coli$Specimens)

## 1 x H uterine lavage
## 1
## 1 x J premate
## 4
## 1 x J uterine
## 1
## 1 x premate J
## 1
## 1x J cliterus
## 1
## 50 ml tube uterine lavage
## 1
## Blood D x 1 , Blood B x 1 , Uterine swab-J , Smear Q x 2
## 1
## Cervical swab-J
## 4
## Cervical Swab J x 1
## 1
## Cervix swab J x 1
## 1
## cliteral swab J x 1
## 1
## Clitoral swab-J
## 1
## Clitoral Swab - J
## 1
## clitoral swab -J
## 1
## clitoral swab J x 1
## 2
## Clitoral swab J x 1
## 1
## Clitoral swab Jx1
## 1
## Clitoral swab x 1
## 2
## Clitorial swab-K
## 1
## Endometrial Swab J x 1 , Histo H x 1
## 1
## EQ PM Swab J pot x 1
## 1
## Eq Premate Swab J
## 1
## Histo H x 1 , Uterine Tissue H x 1
## 1
## PM swab J x 1
## 2
## Pre-mating Swab Jx 1
## 1
## Pre mate swab Jx1
## 1
## Premate swab- J
## 2
## Premate swab - small pot x1
## 1
## Premate Swab 1xJ`
## 1
## premate swab J x 1
## 5
## Premate swab J x 1
## 1
## Premate Swab J x 1
## 7
## Premate swab Jx1
## 1
## Premate Swab pot x 1
## 2
## premate swabJ
## 2
## Prematen Swab K x 1, Premate Swab J x 1
## 1
## Premating Swab J x 1
## 2
## Urterine swab- J
## 1
## Uterine avage fluid H x 1
## 1
## uterine fluid H x 1
## 1
## uterine fluid tube x 1
## 1
## Uterine flush H x 1
## 1
## Uterine Flush H x 1
## 1
## Uterine flush H x 1, Uterine swab-J
## 1
## Uterine Flush Hx 1
## 1
## Uterine J x 1
## 1
## uterine lavage
## 1
## Uterine Lavage
## 1
## Uterine lavage H x 1
## 4
## Uterine Lavage H x 1
## 7
## Uterine Lavage Hx1
## 1
## Uterine lavage in 4 x 50mL tubes
## 1
## Uterine lavage in Hx1
## 1
## Uterine laverage Hx1
## 1
## Uterine swab-J
## 159
## Uterine swab-J , add 30/12
## 1
## Uterine swab-J , Hair H x 1 , Uterine Lavage H x 1
## 1
## Uterine swab-J x 1
## 1
## Uterine swab-J,
## 1
## Uterine swab-J, Slide Q x 1
## 1
## Uterine swab-J, Smear Q x 2
## 1
## Uterine swab-J, Uterine lavage H x 1
## 1
## uterine swab-K
## 1
## Uterine swab-K
## 2
## Uterine swab-L
## 1
## Uterine swab J x 1
## 1
## Uterine Swab J x 1
## 8
## Uterine swabs x3-J
## 1
## uterus fluid H x 1
## 1
## uterus swab-J x 1
## 1
## uterus swab 1xJ
## 1
## Vag discharge H x 1
## 1
## Vaginal swab-J
## 7

summary(e.coli)

## ï..Job_Number ClientCode JobDate Species PostCode
## Min. :397883 Min. : 19.0 20/12/2016: 6 EQU:277 Min. :6055
## 1st Qu.:488857 1st Qu.: 160.0 7/9/2018 : 5 1st Qu.:6121
## Median :575657 Median : 308.0 28/8/2019 : 4 Median :6163
## Mean :584977 Mean : 556.2 18/11/2016: 3 Mean :6192
## 3rd Qu.:688970 3rd Qu.:1025.0 28/9/2017 : 3 3rd Qu.:6231
## Max. :798855 Max. :1240.0 3/11/2015 : 3 Max. :6935
## (Other) :253
## Specimens Prefix Culture Amikacin
## Uterine swab-J :159 F : 0 Haem E.coli : 11 :276
## Uterine Swab J x 1 : 8 M1:277 Non haem E. coli :253 R: 0
## Premate Swab J x 1 : 7 U : 0 Non haem E. coli (mucoid): 1 S: 1
## Uterine Lavage H x 1: 7 Non haem E. coli A : 1 X: 0
## Vaginal swab-J : 7 Non haem E. coli B* : 1
## premate swab J x 1 : 5 Non haem E. coli# : 3
## (Other) : 84 Non haem E. coli* : 7
## Amoxycillin Ampicillin Bactracin Carbenicillin Ceftazidime Ceftiofur
## :273 : 4 :277 :276 :276 : 0
## I: 0 R:141 S: 0 R: 1 R: 1 R: 39
## R: 1 S:132 S: 0 S: 0 S:238
## S: 3 X: 0 X: 0 X: 0 X: 0
## X: 0
##
##
## Cefuroxime Cephalexin Chloramphenicol Ciprofloxacin Clavulox Clavulox_Clavulox
## :277 :277 :277 :276 :277 :277
## S: 0 R: 0 R: 0 I: 1 R: 0 S: 0
## S: 0 S: 0 R: 0 S: 0
## X: 0 X: 0 S: 0 X: 0
##
##
##
## Clindamycin Cloxacillin Doxcycline Doxycycline Doxyycline Enrofloxacin
## :277 :277 :277 :276 :277 : 4
## R: 0 R: 0 R: 0 I: 0 S: 0 I: 6
## S: 0 S: 0 S: 0 R: 1 R: 9
## X: 0 X: 0 S: 0 S:258
## X: 0
##
##
## Enrofloxaxin Erythromycin Fusidic_Acid Gentamicin Lincomycin Marbofloxacin
## :277 :273 :277 : 0 :277 :276
## R: 0 R: 0 R: 0 I: 0 R: 0 I: 0
## S: 0 S: 0 R: 75 S: 0 R: 0
## X: 4 X: 0 S:202 S: 1
## X: 0
##
##
## Minocycline Mupirocin Neomycin Norfloxacin Novobiocin Ofloxacin Penicillin
## :277 :277 : 2 :276 :277 :277 :272
## S: 0 R: 0 I: 1 R: 0 R: 0 R: 0 R: 0
## S: 0 R:193 S: 1 S: 0 S: 0 S: 0
## S: 81 X: 5
## X: 0
##
##
## Penicillin_G Pipercillin Polymyxin_B Rifampicin Soframycin Streptomycin
## : 5 :277 :277 :277 :277 Mode:logical
## R: 0 S: 0 R: 0 R: 0 R: 0 NA's:277
## S: 0 S: 0 S: 0 S: 0
## X:272 X: 0 X: 0 X: 0
##
##
##
## Sulphatrimethoprim Tetracycline Ticarcillin Timentin TestDate
## : 0 : 0 :277 :174 Mode:logical
## R:114 I: 0 R: 0 I: 0 NA's:277
## S:163 R: 64 S: 0 R: 21
## X: 0 S:213 X: 0 S: 82
## X: 0 X: 0
##
##
## ULeucocytes UEpithelial URBC UCasts
## :277 :277 :277 :277
## <1 : 0 <1 : 0 <1 : 0 None seen: 0
## >200 : 0 1 : 0 >200: 0
## 135 : 0 18 : 0 1 : 0
## 2 : 0 2 : 0 116 : 0
## 29 : 0 36 : 0 3 : 0
## (Other): 0 (Other): 0
## UCrystals UBacteria
## :277 :277
## 1+ Ca carbonate : 0 1+ : 0
## 3+ Ca carbonate, Occ Ca oxalate : 0 3+ : 0
## 3+ calcium carbonate : 0 Few : 0
## 3+ Calcium carbonate, few calcium oxalate: 0 Occasional: 0
## Ca carbonate 3+ : 0
## (Other) : 0
## USpermatoza UFungal UDebris UGram USG
## Mode:logical :277 :277 Mode:logical Min. : NA
## NA's:277 2+ Hyphae: 0 1+: 0 NA's:277 1st Qu.: NA
## 3+: 0 Median : NA
## Mean :NaN
## 3rd Qu.: NA
## Max. : NA
## NA's :277
## UPh UProtein UGlucose UKetones UBlood
## :277 :277 :277 :277 :277
## >=9.0: 0 1+ : 0 3+ : 0 1+ : 0 1+ : 0
## 6 : 0 2+ : 0 Negative: 0 Negative: 0 3+ : 0
## 8 : 0 3+ : 0 Trace : 0 Trace : 0 Negative: 0
## 8.5 : 0 Negative: 0
## 9 : 0
##
## UBilirubin UCultureNote M1Leucocytes M1RBC
## :277 :277 :276 :276
## 1+ : 0 >10,000 CFU/mL : 0 OCCASIONAL: 1 3+ : 1
## 3+ : 0 >100,000 CFU/mL: 0 1+ : 0 1+ : 0
## Negative: 0 2,000 CFU/mL : 0 2+ : 0 2+ : 0
## 3,200 CFU/mL : 0 3+ : 0 FEW : 0
## 4,000 CFU/mL : 0 FEW : 0 NONE SEEN: 0
## (Other) : 0 (Other) : 0 (Other) : 0
## M1Epithelial M1Fungal FMicroForExcess
## :276 :276 Mode:logical
## NONE SEEN: 1 NONE SEEN : 1 NA's:277
## 1+ : 0 2+ fungal hyphae : 0
## 2+ : 0 2+ Yeast cells and Fungal Hyphae: 0
## 3+ : 0 FEW * : 0
## FEW : 0 FEW FUNGAL HYPHAE : 0
## (Other) : 0 (Other) : 0
## FLeucocytes FRBC FCampylobacter FYeast
## :277 :277 :277 :277
## None seen: 0 None seen: 0 None seen: 0 None seen : 0
## Very occasional: 0
##
##
##
##
## FProtozoaCystsOva FTrypsin10 FTrypsin100 FOccultBlood
## :277 Mode:logical Mode:logical Mode:logical
## None seen: 0 NA's:277 NA's:277 NA's:277
##
##
##
##
##

enterobacter <- repro_only[grepl("Enterobacter|enterobacter",repro_only$Culture),]
enterobacter$Culture <- as.factor(as.character(enterobacter$Culture))
enterobacter$Specimens <- as.factor(as.character(enterobacter$Specimens))
summary(enterobacter$Culture)

## Enterobacter aerogenes Enterobacter aerogenes* Enterobacter sp.
## 5 2 23
## Enterobacter sp.# Enterobacter sp.*
## 3 9

summary(enterobacter$Specimens)

## 1 x J premate
## 1
## 1 x J uterine lavage
## 1
## 1 x Uterine Container
## 1
## Cervical swab-J
## 3
## Clitoral Swab J x 1
## 1
## Premate Swab J x 1
## 1
## Prematen Swab K x 1, Premate Swab J x 1
## 1
## Uterine flush 1xTube
## 1
## Uterine flush H x 1
## 1
## uterine lavage H
## 1
## Uterine lavage H x 1
## 2
## Uterine Lavage H x 1
## 1
## Uterine swab-J
## 22
## Uterine swab-J , Smear Q x 3 , Histo H x 1
## 1
## uterine swab-L
## 1
## Uterine Swab J x 1
## 1
## Vaginal swab-J
## 1
## Vaginal swab-J, Tissue x 1 H
## 1

summary(enterobacter)

## ï..Job_Number ClientCode JobDate Species PostCode
## Min. :417314 Min. : 19.0 30/11/2017: 3 EQU:42 Min. :6055
## 1st Qu.:537954 1st Qu.: 115.8 2/10/2019 : 2 1st Qu.:6134
## Median :598499 Median : 692.0 10/12/2015: 1 Median :6171
## Mean :614773 Mean : 621.6 12/3/2020 : 1 Mean :6184
## 3rd Qu.:686919 3rd Qu.:1016.2 14/11/2018: 1 3rd Qu.:6214
## Max. :801232 Max. :1240.0 14/3/2017 : 1 Max. :6525
## (Other) :33
## Specimens Prefix Culture Amikacin
## Uterine swab-J :22 F : 0 Enterobacter aerogenes : 5 :42
## Cervical swab-J : 3 M1:42 Enterobacter aerogenes*: 2 R: 0
## Uterine lavage H x 1 : 2 U : 0 Enterobacter sp. :23 S: 0
## 1 x J premate : 1 Enterobacter sp.# : 3 X: 0
## 1 x J uterine lavage : 1 Enterobacter sp.* : 9
## 1 x Uterine Container: 1
## (Other) :12
## Amoxycillin Ampicillin Bactracin Carbenicillin Ceftazidime Ceftiofur
## :40 : 2 :42 :42 :42 : 0
## I: 0 R: 0 S: 0 R: 0 R: 0 R:17
## R: 0 S: 0 S: 0 S: 0 S:25
## S: 0 X:40 X: 0 X: 0 X: 0
## X: 2
##
##
## Cefuroxime Cephalexin Chloramphenicol Ciprofloxacin Clavulox Clavulox_Clavulox
## :42 :42 :42 :42 :42 :42
## S: 0 R: 0 R: 0 I: 0 R: 0 S: 0
## S: 0 S: 0 R: 0 S: 0
## X: 0 X: 0 S: 0 X: 0
##
##
##
## Clindamycin Cloxacillin Doxcycline Doxycycline Doxyycline Enrofloxacin
## :42 :42 :42 :42 :42 : 2
## R: 0 R: 0 R: 0 I: 0 S: 0 I: 5
## S: 0 S: 0 S: 0 R: 0 R: 0
## X: 0 X: 0 S: 0 S:35
## X: 0
##
##
## Enrofloxaxin Erythromycin Fusidic_Acid Gentamicin Lincomycin Marbofloxacin
## :42 :40 :42 : 0 :42 :42
## R: 0 R: 0 R: 0 I: 0 R: 0 I: 0
## S: 0 S: 0 R:16 S: 0 R: 0
## X: 2 X: 0 S:26 S: 0
## X: 0
##
##
## Minocycline Mupirocin Neomycin Norfloxacin Novobiocin Ofloxacin Penicillin
## :42 :42 : 2 :42 :42 :42 :40
## S: 0 R: 0 I: 0 R: 0 R: 0 R: 0 R: 0
## S: 0 R:25 S: 0 S: 0 S: 0 S: 0
## S:15 X: 2
## X: 0
##
##
## Penicillin_G Pipercillin Polymyxin_B Rifampicin Soframycin Streptomycin
## : 2 :42 :42 :42 :42 Mode:logical
## R: 0 S: 0 R: 0 R: 0 R: 0 NA's:42
## S: 0 S: 0 S: 0 S: 0
## X:40 X: 0 X: 0 X: 0
##
##
##
## Sulphatrimethoprim Tetracycline Ticarcillin Timentin TestDate
## : 0 : 0 :42 :35 Mode:logical
## R:16 I: 0 R: 0 I: 0 NA's:42
## S:26 R:18 S: 0 R: 3
## X: 0 S:24 X: 0 S: 4
## X: 0 X: 0
##
##
## ULeucocytes UEpithelial URBC UCasts
## :42 :42 :42 :42
## <1 : 0 <1 : 0 <1 : 0 None seen: 0
## >200 : 0 1 : 0 >200: 0
## 135 : 0 18 : 0 1 : 0
## 2 : 0 2 : 0 116 : 0
## 29 : 0 36 : 0 3 : 0
## (Other): 0 (Other): 0
## UCrystals UBacteria USpermatoza
## :42 :42 Mode:logical
## 1+ Ca carbonate : 0 1+ : 0 NA's:42
## 3+ Ca carbonate, Occ Ca oxalate : 0 3+ : 0
## 3+ calcium carbonate : 0 Few : 0
## 3+ Calcium carbonate, few calcium oxalate: 0 Occasional: 0
## Ca carbonate 3+ : 0
## (Other) : 0
## UFungal UDebris UGram USG UPh UProtein
## :42 :42 Mode:logical Min. : NA :42 :42
## 2+ Hyphae: 0 1+: 0 NA's:42 1st Qu.: NA >=9.0: 0 1+ : 0
## 3+: 0 Median : NA 6 : 0 2+ : 0
## Mean :NaN 8 : 0 3+ : 0
## 3rd Qu.: NA 8.5 : 0 Negative: 0
## Max. : NA 9 : 0
## NA's :42
## UGlucose UKetones UBlood UBilirubin UCultureNote
## :42 :42 :42 :42 :42
## 3+ : 0 1+ : 0 1+ : 0 1+ : 0 >10,000 CFU/mL : 0
## Negative: 0 Negative: 0 3+ : 0 3+ : 0 >100,000 CFU/mL: 0
## Trace : 0 Trace : 0 Negative: 0 Negative: 0 2,000 CFU/mL : 0
## 3,200 CFU/mL : 0
## 4,000 CFU/mL : 0
## (Other) : 0
## M1Leucocytes M1RBC M1Epithelial
## :42 :42 :42
## 1+ : 0 1+ : 0 1+ : 0
## 2+ : 0 2+ : 0 2+ : 0
## 3+ : 0 3+ : 0 3+ : 0
## FEW : 0 FEW : 0 FEW : 0
## NONE SEEN: 0 NONE SEEN: 0 NONE SEEN: 0
## (Other) : 0 (Other) : 0 (Other) : 0
## M1Fungal FMicroForExcess FLeucocytes
## :42 Mode:logical :42
## 2+ fungal hyphae : 0 NA's:42 None seen: 0
## 2+ Yeast cells and Fungal Hyphae: 0
## FEW * : 0
## FEW FUNGAL HYPHAE : 0
## FUNGAL HYPHAE 1+ : 0
## (Other) : 0
## FRBC FCampylobacter FYeast FProtozoaCystsOva
## :42 :42 :42 :42
## None seen: 0 None seen: 0 None seen : 0 None seen: 0
## Very occasional: 0
##
##
##
##
## FTrypsin10 FTrypsin100 FOccultBlood
## Mode:logical Mode:logical Mode:logical
## NA's:42 NA's:42 NA's:42
##
##
##
##
##

enterococcus <- repro_only[grepl("Enterococcus|enterococcus",repro_only$Culture),]
enterococcus$Culture <- as.factor(as.character(enterococcus$Culture))
enterococcus$Specimens <- as.factor(as.character(enterococcus$Specimens))
summary(enterococcus$Culture)

## Enterococcus casseliflavus Enterococcus faecalis
## 1 1
## Enterococcus sp. Enterococcus sp. (beta haemolytic)
## 48 1
## Enterococcus sp.*
## 4

summary(enterococcus$Specimens)

## 1 x J premate 1 x J uterine
## 1 1
## 1 x J uterine lavage Cervical swab-J
## 1 3
## Clitoral swab J x 1 Clitorial swab-K
## 2 1
## Pre-mating Swab Jx 1 Premate Clittoral J
## 1 1
## Premate Swab J x 1 Prematen Swab K x 1, Premate Swab J x 1
## 1 1
## Uterine flush 1x-H Uterine flush H x 1, Uterine swab-J
## 1 1
## Uterine lavage-J, Agar media-H Uterine swab-J
## 1 34
## Uterine Swab J x 1 Uterus lesion swab-J
## 3 1
## Vaginal swab-J
## 1

summary(enterococcus)

## ï..Job_Number ClientCode JobDate Species PostCode
## Min. :396877 Min. : 19.0 7/9/2018 : 7 EQU:55 Min. :6055
## 1st Qu.:513257 1st Qu.: 101.0 6/9/2019 : 3 1st Qu.:6112
## Median :655981 Median : 308.0 10/10/2018: 2 Median :6171
## Mean :633686 Mean : 571.1 21/10/2019: 2 Mean :6191
## 3rd Qu.:744188 3rd Qu.:1131.0 5/11/2018 : 2 3rd Qu.:6231
## Max. :777158 Max. :1240.0 1/10/2019 : 1 Max. :6530
## (Other) :38
## Specimens Prefix Culture
## Uterine swab-J :34 F : 0 Enterococcus casseliflavus : 1
## Cervical swab-J : 3 M1:55 Enterococcus faecalis : 1
## Uterine Swab J x 1 : 3 U : 0 Enterococcus sp. :48
## Clitoral swab J x 1: 2 Enterococcus sp. (beta haemolytic): 1
## 1 x J premate : 1 Enterococcus sp.* : 4
## 1 x J uterine : 1
## (Other) :11
## Amikacin Amoxycillin Ampicillin Bactracin Carbenicillin Ceftazidime Ceftiofur
## :55 :55 : 0 :55 :55 :55 : 0
## R: 0 I: 0 R: 5 S: 0 R: 0 R: 0 R: 0
## S: 0 R: 0 S:50 S: 0 S: 0 S: 0
## X: 0 S: 0 X: 0 X: 0 X: 0 X:55
## X: 0
##
##
## Cefuroxime Cephalexin Chloramphenicol Ciprofloxacin Clavulox Clavulox_Clavulox
## :55 :55 :54 :53 :55 :55
## S: 0 R: 0 R: 0 I: 1 R: 0 S: 0
## S: 0 S: 1 R: 1 S: 0
## X: 0 X: 0 S: 0 X: 0
##
##
##
## Clindamycin Cloxacillin Doxcycline Doxycycline Doxyycline Enrofloxacin
## :55 :55 :55 :55 :55 : 0
## R: 0 R: 0 R: 0 I: 0 S: 0 I:37
## S: 0 S: 0 S: 0 R: 0 R:13
## X: 0 X: 0 S: 0 S: 5
## X: 0
##
##
## Enrofloxaxin Erythromycin Fusidic_Acid Gentamicin Lincomycin Marbofloxacin
## :55 :55 :55 : 0 :55 :54
## R: 0 R: 0 R: 0 I: 0 R: 0 I: 1
## S: 0 S: 0 R: 0 S: 0 R: 0
## X: 0 X: 0 S: 0 S: 0
## X:55
##
##
## Minocycline Mupirocin Neomycin Norfloxacin Novobiocin Ofloxacin Penicillin
## :55 :54 : 0 :54 :55 :55 :55
## S: 0 R: 1 I: 0 R: 1 R: 0 R: 0 R: 0
## S: 0 R: 0 S: 0 S: 0 S: 0 S: 0
## S: 0 X: 0
## X:55
##
##
## Penicillin_G Pipercillin Polymyxin_B Rifampicin Soframycin Streptomycin
## : 0 :55 :55 :54 :55 Mode:logical
## R:16 S: 0 R: 0 R: 1 R: 0 NA's:55
## S:39 S: 0 S: 0 S: 0
## X: 0 X: 0 X: 0 X: 0
##
##
##
## Sulphatrimethoprim Tetracycline Ticarcillin Timentin TestDate
## : 0 : 0 :55 :41 Mode:logical
## R: 0 I: 0 R: 0 I: 0 NA's:55
## S: 0 R:22 S: 0 R: 0
## X:55 S:33 X: 0 S: 0
## X: 0 X:14
##
##
## ULeucocytes UEpithelial URBC UCasts
## :55 :55 :55 :55
## <1 : 0 <1 : 0 <1 : 0 None seen: 0
## >200 : 0 1 : 0 >200: 0
## 135 : 0 18 : 0 1 : 0
## 2 : 0 2 : 0 116 : 0
## 29 : 0 36 : 0 3 : 0
## (Other): 0 (Other): 0
## UCrystals UBacteria USpermatoza
## :55 :55 Mode:logical
## 1+ Ca carbonate : 0 1+ : 0 NA's:55
## 3+ Ca carbonate, Occ Ca oxalate : 0 3+ : 0
## 3+ calcium carbonate : 0 Few : 0
## 3+ Calcium carbonate, few calcium oxalate: 0 Occasional: 0
## Ca carbonate 3+ : 0
## (Other) : 0
## UFungal UDebris UGram USG UPh UProtein
## :55 :55 Mode:logical Min. : NA :55 :55
## 2+ Hyphae: 0 1+: 0 NA's:55 1st Qu.: NA >=9.0: 0 1+ : 0
## 3+: 0 Median : NA 6 : 0 2+ : 0
## Mean :NaN 8 : 0 3+ : 0
## 3rd Qu.: NA 8.5 : 0 Negative: 0
## Max. : NA 9 : 0
## NA's :55
## UGlucose UKetones UBlood UBilirubin UCultureNote
## :55 :55 :55 :55 :55
## 3+ : 0 1+ : 0 1+ : 0 1+ : 0 >10,000 CFU/mL : 0
## Negative: 0 Negative: 0 3+ : 0 3+ : 0 >100,000 CFU/mL: 0
## Trace : 0 Trace : 0 Negative: 0 Negative: 0 2,000 CFU/mL : 0
## 3,200 CFU/mL : 0
## 4,000 CFU/mL : 0
## (Other) : 0
## M1Leucocytes M1RBC M1Epithelial
## :55 :55 :55
## 1+ : 0 1+ : 0 1+ : 0
## 2+ : 0 2+ : 0 2+ : 0
## 3+ : 0 3+ : 0 3+ : 0
## FEW : 0 FEW : 0 FEW : 0
## NONE SEEN: 0 NONE SEEN: 0 NONE SEEN: 0
## (Other) : 0 (Other) : 0 (Other) : 0
## M1Fungal FMicroForExcess FLeucocytes
## :55 Mode:logical :55
## 2+ fungal hyphae : 0 NA's:55 None seen: 0
## 2+ Yeast cells and Fungal Hyphae: 0
## FEW * : 0
## FEW FUNGAL HYPHAE : 0
## FUNGAL HYPHAE 1+ : 0
## (Other) : 0
## FRBC FCampylobacter FYeast FProtozoaCystsOva
## :55 :55 :55 :55
## None seen: 0 None seen: 0 None seen : 0 None seen: 0
## Very occasional: 0
##
##
##
##
## FTrypsin10 FTrypsin100 FOccultBlood
## Mode:logical Mode:logical Mode:logical
## NA's:55 NA's:55 NA's:55
##
##
##
##
##

pseudomonas <- repro_only[grepl("Pseudomonas|pseudomonas",repro_only$Culture),]
pseudomonas$Culture <- as.factor(as.character(pseudomonas$Culture))
pseudomonas$Specimens <- as.factor(as.character(pseudomonas$Specimens))
summary(pseudomonas$Culture)

## Pseudomonas aeruginosa Pseudomonas fluorecsns Pseudomonas fluorescens
## 28 1 1
## Pseudomonas oryzihabitans Pseudomonas putida Pseudomonas sp.
## 1 1 2
## Pseudomonas sp.* Pseudomonas stutzeri Pseudomonas stutzeri*
## 2 1 2

summary(pseudomonas$Specimens)

## 1 x J premate 1 x J uterine biopsy
## 1 1
## 1 x J uterine lavage Clitoral swab x 1
## 1 3
## Mare Endometritis Swab J x 1 premate swabJ
## 1 1
## Premating swab Jx1, Smear Qx1 Swab "mare" J
## 1 1
## Uterine Fluid D x 1 Uterine Fluid H x 1
## 1 1
## uterine fluid tubes x 2 Uterine flush D x 1
## 1 1
## Uterine Lavage H x 1 Uterine swab-J
## 1 20
## Uterine swab-J, Smear Q x 1 uterine swab-K
## 1 1
## Uterine swab J x 1 Vaginal swab-J
## 1 1

summary(pseudomonas)

## ï..Job_Number ClientCode JobDate Species PostCode
## Min. :403701 Min. : 19.0 9/12/2019 : 3 EQU:39 Min. :6069
## 1st Qu.:492269 1st Qu.: 130.5 23/11/2017: 2 1st Qu.:6121
## Median :588128 Median : 160.0 1/3/2016 : 1 Median :6171
## Mean :572713 Mean : 410.9 12/1/2016 : 1 Mean :6189
## 3rd Qu.:643115 3rd Qu.: 692.0 12/11/2015: 1 3rd Qu.:6214
## Max. :766054 Max. :1240.0 12/2/2018 : 1 Max. :6751
## (Other) :30
## Specimens Prefix Culture
## Uterine swab-J :20 F : 0 Pseudomonas aeruginosa :28
## Clitoral swab x 1 : 3 M1:39 Pseudomonas sp. : 2
## 1 x J premate : 1 U : 0 Pseudomonas sp.* : 2
## 1 x J uterine biopsy : 1 Pseudomonas stutzeri* : 2
## 1 x J uterine lavage : 1 Pseudomonas fluorecsns : 1
## Mare Endometritis Swab J x 1: 1 Pseudomonas fluorescens: 1
## (Other) :12 (Other) : 3
## Amikacin Amoxycillin Ampicillin Bactracin Carbenicillin Ceftazidime Ceftiofur
## :38 :39 : 0 :39 :38 :38 : 0
## R: 1 I: 0 R:10 S: 0 R: 1 R: 0 R: 8
## S: 0 R: 0 S: 1 S: 0 S: 1 S: 3
## X: 0 S: 0 X:28 X: 0 X: 0 X:28
## X: 0
##
##
## Cefuroxime Cephalexin Chloramphenicol Ciprofloxacin Clavulox Clavulox_Clavulox
## :39 :39 :39 :39 :39 :39
## S: 0 R: 0 R: 0 I: 0 R: 0 S: 0
## S: 0 S: 0 R: 0 S: 0
## X: 0 X: 0 S: 0 X: 0
##
##
##
## Clindamycin Cloxacillin Doxcycline Doxycycline Doxyycline Enrofloxacin
## :39 :39 :39 :39 :39 : 0
## R: 0 R: 0 R: 0 I: 0 S: 0 I:22
## S: 0 S: 0 S: 0 R: 0 R: 7
## X: 0 X: 0 S: 0 S:10
## X: 0
##
##
## Enrofloxaxin Erythromycin Fusidic_Acid Gentamicin Lincomycin Marbofloxacin
## :39 :39 :39 : 0 :39 :38
## R: 0 R: 0 R: 0 I: 0 R: 0 I: 0
## S: 0 S: 0 R:14 S: 0 R: 0
## X: 0 X: 0 S:25 S: 1
## X: 0
##
##
## Minocycline Mupirocin Neomycin Norfloxacin Novobiocin Ofloxacin Penicillin
## :39 :39 : 0 :38 :39 :39 :39
## S: 0 R: 0 I: 0 R: 0 R: 0 R: 0 R: 0
## S: 0 R:24 S: 1 S: 0 S: 0 S: 0
## S:15 X: 0
## X: 0
##
##
## Penicillin_G Pipercillin Polymyxin_B Rifampicin Soframycin Streptomycin
## : 0 :39 :39 :39 :39 Mode:logical
## R:11 S: 0 R: 0 R: 0 R: 0 NA's:39
## S: 0 S: 0 S: 0 S: 0
## X:28 X: 0 X: 0 X: 0
##
##
##
## Sulphatrimethoprim Tetracycline Ticarcillin Timentin TestDate
## : 0 : 0 :38 :27 Mode:logical
## R: 7 I: 0 R: 0 I: 0 NA's:39
## S: 4 R: 2 S: 1 R: 5
## X:28 S: 9 X: 0 S: 7
## X:28 X: 0
##
##
## ULeucocytes UEpithelial URBC UCasts
## :39 :39 :39 :39
## <1 : 0 <1 : 0 <1 : 0 None seen: 0
## >200 : 0 1 : 0 >200: 0
## 135 : 0 18 : 0 1 : 0
## 2 : 0 2 : 0 116 : 0
## 29 : 0 36 : 0 3 : 0
## (Other): 0 (Other): 0
## UCrystals UBacteria USpermatoza
## :39 :39 Mode:logical
## 1+ Ca carbonate : 0 1+ : 0 NA's:39
## 3+ Ca carbonate, Occ Ca oxalate : 0 3+ : 0
## 3+ calcium carbonate : 0 Few : 0
## 3+ Calcium carbonate, few calcium oxalate: 0 Occasional: 0
## Ca carbonate 3+ : 0
## (Other) : 0
## UFungal UDebris UGram USG UPh UProtein
## :39 :39 Mode:logical Min. : NA :39 :39
## 2+ Hyphae: 0 1+: 0 NA's:39 1st Qu.: NA >=9.0: 0 1+ : 0
## 3+: 0 Median : NA 6 : 0 2+ : 0
## Mean :NaN 8 : 0 3+ : 0
## 3rd Qu.: NA 8.5 : 0 Negative: 0
## Max. : NA 9 : 0
## NA's :39
## UGlucose UKetones UBlood UBilirubin UCultureNote
## :39 :39 :39 :39 :39
## 3+ : 0 1+ : 0 1+ : 0 1+ : 0 >10,000 CFU/mL : 0
## Negative: 0 Negative: 0 3+ : 0 3+ : 0 >100,000 CFU/mL: 0
## Trace : 0 Trace : 0 Negative: 0 Negative: 0 2,000 CFU/mL : 0
## 3,200 CFU/mL : 0
## 4,000 CFU/mL : 0
## (Other) : 0
## M1Leucocytes M1RBC M1Epithelial
## :39 :39 :39
## 1+ : 0 1+ : 0 1+ : 0
## 2+ : 0 2+ : 0 2+ : 0
## 3+ : 0 3+ : 0 3+ : 0
## FEW : 0 FEW : 0 FEW : 0
## NONE SEEN: 0 NONE SEEN: 0 NONE SEEN: 0
## (Other) : 0 (Other) : 0 (Other) : 0
## M1Fungal FMicroForExcess FLeucocytes
## :39 Mode:logical :39
## 2+ fungal hyphae : 0 NA's:39 None seen: 0
## 2+ Yeast cells and Fungal Hyphae: 0
## FEW * : 0
## FEW FUNGAL HYPHAE : 0
## FUNGAL HYPHAE 1+ : 0
## (Other) : 0
## FRBC FCampylobacter FYeast FProtozoaCystsOva
## :39 :39 :39 :39
## None seen: 0 None seen: 0 None seen : 0 None seen: 0
## Very occasional: 0
##
##
##
##
## FTrypsin10 FTrypsin100 FOccultBlood
## Mode:logical Mode:logical Mode:logical
## NA's:39 NA's:39 NA's:39
##
##
##
##
##

corynebacterium <- repro_only[grepl("Corynebacterium|corynebacterium",repro_only$Culture),]
corynebacterium$Culture <- as.factor(as.character(corynebacterium$Culture))
corynebacterium$Specimens <- as.factor(as.character(corynebacterium$Specimens))
summary(corynebacterium$Culture)

## Corynebacterium glucuronolyticum Corynebacterium propinquum
## 1 1
## Corynebacterium sp. Corynebacterium sp.*
## 6 1
## Corynebacterium urealyticum
## 1

summary(corynebacterium$Specimens)

## 1 x Premate Cervical swab-J Premate J x 1 Uterine Fluid D x 2
## 1 1 1 1
## Uterine Fluid x H Uterine swab-J
## 1 5

summary(corynebacterium)

## ï..Job_Number ClientCode JobDate Species PostCode
## Min. :400933 Min. : 63.0 1/11/2018 :1 EQU:10 Min. :6055
## 1st Qu.:451485 1st Qu.: 161.0 1/9/2017 :1 1st Qu.:6122
## Median :509782 Median : 692.0 13/12/2016:1 Median :6192
## Mean :519125 Mean : 536.2 16/11/2018:1 Mean :6173
## 3rd Qu.:554421 3rd Qu.: 692.0 16/12/2016:1 3rd Qu.:6214
## Max. :679295 Max. :1131.0 19/8/2015 :1 Max. :6330
## (Other) :4
## Specimens Prefix Culture Amikacin
## 1 x Premate :1 F : 0 Corynebacterium glucuronolyticum:1 :10
## Cervical swab-J :1 M1:10 Corynebacterium propinquum :1 R: 0
## Premate J x 1 :1 U : 0 Corynebacterium sp. :6 S: 0
## Uterine Fluid D x 2:1 Corynebacterium sp.* :1 X: 0
## Uterine Fluid x H :1 Corynebacterium urealyticum :1
## Uterine swab-J :5
##
## Amoxycillin Ampicillin Bactracin Carbenicillin Ceftazidime Ceftiofur
## :9 :1 :10 :10 :10 :0
## I:0 R:1 S: 0 R: 0 R: 0 R:1
## R:0 S:8 S: 0 S: 0 S:9
## S:1 X:0 X: 0 X: 0 X:0
## X:0
##
##
## Cefuroxime Cephalexin Chloramphenicol Ciprofloxacin Clavulox Clavulox_Clavulox
## :10 :10 :10 :10 :10 :10
## S: 0 R: 0 R: 0 I: 0 R: 0 S: 0
## S: 0 S: 0 R: 0 S: 0
## X: 0 X: 0 S: 0 X: 0
##
##
##
## Clindamycin Cloxacillin Doxcycline Doxycycline Doxyycline Enrofloxacin
## :10 :10 :10 :10 :10 :1
## R: 0 R: 0 R: 0 I: 0 S: 0 I:0
## S: 0 S: 0 S: 0 R: 0 R:0
## X: 0 X: 0 S: 0 S:9
## X: 0
##
##
## Enrofloxaxin Erythromycin Fusidic_Acid Gentamicin Lincomycin Marbofloxacin
## :10 :9 :10 : 0 :10 :10
## R: 0 R:0 R: 0 I: 0 R: 0 I: 0
## S:1 S: 0 R: 0 S: 0 R: 0
## X:0 X: 0 S:10 S: 0
## X: 0
##
##
## Minocycline Mupirocin Neomycin Norfloxacin Novobiocin Ofloxacin Penicillin
## :10 :10 : 0 :10 :10 :10 :9
## S: 0 R: 0 I: 0 R: 0 R: 0 R: 0 R:0
## S: 0 R: 0 S: 0 S: 0 S: 0 S:1
## S:10 X:0
## X: 0
##
##
## Penicillin_G Pipercillin Polymyxin_B Rifampicin Soframycin Streptomycin
## :1 :10 :10 :10 :10 Mode:logical
## R:1 S: 0 R: 0 R: 0 R: 0 NA's:10
## S:8 S: 0 S: 0 S: 0
## X:0 X: 0 X: 0 X: 0
##
##
##
## Sulphatrimethoprim Tetracycline Ticarcillin Timentin TestDate
## : 0 : 0 :10 :5 Mode:logical
## R: 0 I: 0 R: 0 I:0 NA's:10
## S:10 R: 0 S: 0 R:0
## X: 0 S:10 X: 0 S:5
## X: 0 X:0
##
##
## ULeucocytes UEpithelial URBC UCasts
## :10 :10 :10 :10
## <1 : 0 <1 : 0 <1 : 0 None seen: 0
## >200 : 0 1 : 0 >200: 0
## 135 : 0 18 : 0 1 : 0
## 2 : 0 2 : 0 116 : 0
## 29 : 0 36 : 0 3 : 0
## (Other): 0 (Other): 0
## UCrystals UBacteria USpermatoza
## :10 :10 Mode:logical
## 1+ Ca carbonate : 0 1+ : 0 NA's:10
## 3+ Ca carbonate, Occ Ca oxalate : 0 3+ : 0
## 3+ calcium carbonate : 0 Few : 0
## 3+ Calcium carbonate, few calcium oxalate: 0 Occasional: 0
## Ca carbonate 3+ : 0
## (Other) : 0
## UFungal UDebris UGram USG UPh UProtein
## :10 :10 Mode:logical Min. : NA :10 :10
## 2+ Hyphae: 0 1+: 0 NA's:10 1st Qu.: NA >=9.0: 0 1+ : 0
## 3+: 0 Median : NA 6 : 0 2+ : 0
## Mean :NaN 8 : 0 3+ : 0
## 3rd Qu.: NA 8.5 : 0 Negative: 0
## Max. : NA 9 : 0
## NA's :10
## UGlucose UKetones UBlood UBilirubin UCultureNote
## :10 :10 :10 :10 :10
## 3+ : 0 1+ : 0 1+ : 0 1+ : 0 >10,000 CFU/mL : 0
## Negative: 0 Negative: 0 3+ : 0 3+ : 0 >100,000 CFU/mL: 0
## Trace : 0 Trace : 0 Negative: 0 Negative: 0 2,000 CFU/mL : 0
## 3,200 CFU/mL : 0
## 4,000 CFU/mL : 0
## (Other) : 0
## M1Leucocytes M1RBC M1Epithelial
## :10 :10 :10
## 1+ : 0 1+ : 0 1+ : 0
## 2+ : 0 2+ : 0 2+ : 0
## 3+ : 0 3+ : 0 3+ : 0
## FEW : 0 FEW : 0 FEW : 0
## NONE SEEN: 0 NONE SEEN: 0 NONE SEEN: 0
## (Other) : 0 (Other) : 0 (Other) : 0
## M1Fungal FMicroForExcess FLeucocytes
## :10 Mode:logical :10
## 2+ fungal hyphae : 0 NA's:10 None seen: 0
## 2+ Yeast cells and Fungal Hyphae: 0
## FEW * : 0
## FEW FUNGAL HYPHAE : 0
## FUNGAL HYPHAE 1+ : 0
## (Other) : 0
## FRBC FCampylobacter FYeast FProtozoaCystsOva
## :10 :10 :10 :10
## None seen: 0 None seen: 0 None seen : 0 None seen: 0
## Very occasional: 0
##
##
##
##
## FTrypsin10 FTrypsin100 FOccultBlood
## Mode:logical Mode:logical Mode:logical
## NA's:10 NA's:10 NA's:10
##
##
##
##
##

klebsiella <- repro_only[grepl("Klebsiella|klebsiella",repro_only$Culture),]
klebsiella$Culture <- as.factor(as.character(klebsiella$Culture))
klebsiella$Specimens <- as.factor(as.character(klebsiella$Specimens))
summary(klebsiella$Culture)

## Klebsiella oxytoca Klebsiella oxytoca* Klebsiella pneumoniae
## 5 1 18
## Klebsiella pneumoniae*
## 5

summary(klebsiella$Specimens)

## 1 x premate J Cervical swab-J
## 1 1
## Clittoral swab -J Mare Flush H x 1
## 1 1
## Premate Swab J x 1 Uterine Fluid H x 1
## 1 1
## Uterine Fluid H x 1 , add 29/11 Uterine flush H x 1
## 1 1
## Uterine Lavage Uterine Lavage H x 1
## 1 3
## Uterine swab-J Uterine swab-L
## 14 2
## uterus swab-J
## 1

summary(klebsiella)

## ï..Job_Number ClientCode JobDate Species PostCode
## Min. :412841 Min. : 68.0 12/7/2016 : 2 EQU:29 Min. :6055
## 1st Qu.:470489 1st Qu.: 165.0 1/9/2018 : 1 1st Qu.:6121
## Median :564952 Median : 807.0 10/10/2016: 1 Median :6171
## Mean :577600 Mean : 727.1 11/2/2017 : 1 Mean :6229
## 3rd Qu.:662548 3rd Qu.:1053.0 12/12/2019: 1 3rd Qu.:6214
## Max. :772270 Max. :1240.0 14/10/2015: 1 Max. :6931
## (Other) :22
## Specimens Prefix Culture Amikacin
## Uterine swab-J :14 F : 0 Klebsiella oxytoca : 5 :28
## Uterine Lavage H x 1: 3 M1:29 Klebsiella oxytoca* : 1 R: 0
## Uterine swab-L : 2 U : 0 Klebsiella pneumoniae :18 S: 1
## 1 x premate J : 1 Klebsiella pneumoniae*: 5 X: 0
## Cervical swab-J : 1
## Clittoral swab -J : 1
## (Other) : 7
## Amoxycillin Ampicillin Bactracin Carbenicillin Ceftazidime Ceftiofur
## :29 : 0 :29 :28 :28 : 0
## I: 0 R: 0 S: 0 R: 1 R: 0 R:11
## R: 0 S: 0 S: 0 S: 1 S:18
## S: 0 X:29 X: 0 X: 0 X: 0
## X: 0
##
##
## Cefuroxime Cephalexin Chloramphenicol Ciprofloxacin Clavulox Clavulox_Clavulox
## :29 :29 :29 :28 :29 :29
## S: 0 R: 0 R: 0 I: 0 R: 0 S: 0
## S: 0 S: 0 R: 0 S: 0
## X: 0 X: 0 S: 1 X: 0
##
##
##
## Clindamycin Cloxacillin Doxcycline Doxycycline Doxyycline Enrofloxacin
## :29 :29 :29 :29 :29 : 0
## R: 0 R: 0 R: 0 I: 0 S: 0 I: 5
## S: 0 S: 0 S: 0 R: 0 R: 4
## X: 0 X: 0 S: 0 S:20
## X: 0
##
##
## Enrofloxaxin Erythromycin Fusidic_Acid Gentamicin Lincomycin Marbofloxacin
## :29 :29 :29 : 0 :29 :28
## R: 0 R: 0 R: 0 I: 0 R: 0 I: 0
## S: 0 S: 0 R:10 S: 0 R: 0
## X: 0 X: 0 S:19 S: 1
## X: 0
##
##
## Minocycline Mupirocin Neomycin Norfloxacin Novobiocin Ofloxacin Penicillin
## :29 :29 : 0 :28 :29 :29 :29
## S: 0 R: 0 I: 0 R: 0 R: 0 R: 0 R: 0
## S: 0 R:11 S: 1 S: 0 S: 0 S: 0
## S:18 X: 0
## X: 0
##
##
## Penicillin_G Pipercillin Polymyxin_B Rifampicin Soframycin Streptomycin
## : 0 :29 :29 :29 :29 Mode:logical
## R: 0 S: 0 R: 0 R: 0 R: 0 NA's:29
## S: 0 S: 0 S: 0 S: 0
## X:29 X: 0 X: 0 X: 0
##
##
##
## Sulphatrimethoprim Tetracycline Ticarcillin Timentin TestDate
## : 0 : 0 :28 :18 Mode:logical
## R:13 I: 0 R: 1 I: 0 NA's:29
## S:16 R: 9 S: 0 R: 0
## X: 0 S:20 X: 0 S: 0
## X: 0 X:11
##
##
## ULeucocytes UEpithelial URBC UCasts
## :29 :29 :29 :29
## <1 : 0 <1 : 0 <1 : 0 None seen: 0
## >200 : 0 1 : 0 >200: 0
## 135 : 0 18 : 0 1 : 0
## 2 : 0 2 : 0 116 : 0
## 29 : 0 36 : 0 3 : 0
## (Other): 0 (Other): 0
## UCrystals UBacteria USpermatoza
## :29 :29 Mode:logical
## 1+ Ca carbonate : 0 1+ : 0 NA's:29
## 3+ Ca carbonate, Occ Ca oxalate : 0 3+ : 0
## 3+ calcium carbonate : 0 Few : 0
## 3+ Calcium carbonate, few calcium oxalate: 0 Occasional: 0
## Ca carbonate 3+ : 0
## (Other) : 0
## UFungal UDebris UGram USG UPh UProtein
## :29 :29 Mode:logical Min. : NA :29 :29
## 2+ Hyphae: 0 1+: 0 NA's:29 1st Qu.: NA >=9.0: 0 1+ : 0
## 3+: 0 Median : NA 6 : 0 2+ : 0
## Mean :NaN 8 : 0 3+ : 0
## 3rd Qu.: NA 8.5 : 0 Negative: 0
## Max. : NA 9 : 0
## NA's :29
## UGlucose UKetones UBlood UBilirubin UCultureNote
## :29 :29 :29 :29 :29
## 3+ : 0 1+ : 0 1+ : 0 1+ : 0 >10,000 CFU/mL : 0
## Negative: 0 Negative: 0 3+ : 0 3+ : 0 >100,000 CFU/mL: 0
## Trace : 0 Trace : 0 Negative: 0 Negative: 0 2,000 CFU/mL : 0
## 3,200 CFU/mL : 0
## 4,000 CFU/mL : 0
## (Other) : 0
## M1Leucocytes M1RBC M1Epithelial
## :29 :29 :29
## 1+ : 0 1+ : 0 1+ : 0
## 2+ : 0 2+ : 0 2+ : 0
## 3+ : 0 3+ : 0 3+ : 0
## FEW : 0 FEW : 0 FEW : 0
## NONE SEEN: 0 NONE SEEN: 0 NONE SEEN: 0
## (Other) : 0 (Other) : 0 (Other) : 0
## M1Fungal FMicroForExcess FLeucocytes
## :29 Mode:logical :29
## 2+ fungal hyphae : 0 NA's:29 None seen: 0
## 2+ Yeast cells and Fungal Hyphae: 0
## FEW * : 0
## FEW FUNGAL HYPHAE : 0
## FUNGAL HYPHAE 1+ : 0
## (Other) : 0
## FRBC FCampylobacter FYeast FProtozoaCystsOva
## :29 :29 :29 :29
## None seen: 0 None seen: 0 None seen : 0 None seen: 0
## Very occasional: 0
##
##
##
##
## FTrypsin10 FTrypsin100 FOccultBlood
## Mode:logical Mode:logical Mode:logical
## NA's:29 NA's:29 NA's:29
##
##
##
##
##

rhizobium <- repro_only[grepl("Rhizobium|rhizobium",repro_only$Culture),]
rhizobium$Culture <- as.factor(as.character(rhizobium$Culture))
rhizobium$Specimens <- as.factor(as.character(rhizobium$Specimens))
summary(rhizobium$Culture)

## Rhizobium radiobacter
## 1

summary(rhizobium$Specimens)

## Cervical swab-J
## 1

summary(rhizobium)

## ï..Job_Number ClientCode JobDate Species PostCode
## Min. :496230 Min. :84 18/10/2016:1 EQU:1 Min. :6503
## 1st Qu.:496230 1st Qu.:84 1/1/2016 :0 1st Qu.:6503
## Median :496230 Median :84 1/10/2015 :0 Median :6503
## Mean :496230 Mean :84 1/10/2019 :0 Mean :6503
## 3rd Qu.:496230 3rd Qu.:84 1/11/2016 :0 3rd Qu.:6503
## Max. :496230 Max. :84 1/11/2017 :0 Max. :6503
## (Other) :0
## Specimens Prefix Culture Amikacin Amoxycillin
## Cervical swab-J:1 F :0 Rhizobium radiobacter:1 :1 :1
## M1:1 R:0 I:0
## U :0 S:0 R:0
## X:0 S:0
## X:0
##
##
## Ampicillin Bactracin Carbenicillin Ceftazidime Ceftiofur Cefuroxime Cephalexin
## :0 :1 :1 :1 :0 :1 :1
## R:0 S:0 R:0 R:0 R:0 S:0 R:0
## S:1 S:0 S:0 S:1 S:0
## X:0 X:0 X:0 X:0 X:0
##
##
##
## Chloramphenicol Ciprofloxacin Clavulox Clavulox_Clavulox Clindamycin
## :1 :1 :1 :1 :1
## R:0 I:0 R:0 S:0 R:0
## S:0 R:0 S:0 S:0
## X:0 S:0 X:0 X:0
##
##
##
## Cloxacillin Doxcycline Doxycycline Doxyycline Enrofloxacin Enrofloxaxin
## :1 :1 :1 :1 :0 :1
## R:0 R:0 I:0 S:0 I:1 R:0
## S:0 S:0 R:0 R:0
## X:0 S:0 S:0
## X:0
##
##
## Erythromycin Fusidic_Acid Gentamicin Lincomycin Marbofloxacin Minocycline
## :1 :1 :0 :1 :1 :1
## R:0 R:0 I:0 R:0 I:0 S:0
## S:0 S:0 R:0 S:0 R:0
## X:0 X:0 S:1 S:0
## X:0
##
##
## Mupirocin Neomycin Norfloxacin Novobiocin Ofloxacin Penicillin Penicillin_G
## :1 :0 :1 :1 :1 :1 :0
## R:0 I:0 R:0 R:0 R:0 R:0 R:0
## S:0 R:0 S:0 S:0 S:0 S:0 S:1
## S:1 X:0 X:0
## X:0
##
##
## Pipercillin Polymyxin_B Rifampicin Soframycin Streptomycin
## :1 :1 :1 :1 Mode:logical
## S:0 R:0 R:0 R:0 NA's:1
## S:0 S:0 S:0
## X:0 X:0 X:0
##
##
##
## Sulphatrimethoprim Tetracycline Ticarcillin Timentin TestDate
## :0 :0 :1 :0 Mode:logical
## R:0 I:0 R:0 I:0 NA's:1
## S:1 R:0 S:0 R:0
## X:0 S:1 X:0 S:1
## X:0 X:0
##
##
## ULeucocytes UEpithelial URBC UCasts
## :1 :1 :1 :1
## <1 :0 <1 :0 <1 :0 None seen:0
## >200 :0 1 :0 >200:0
## 135 :0 18 :0 1 :0
## 2 :0 2 :0 116 :0
## 29 :0 36 :0 3 :0
## (Other):0 (Other):0
## UCrystals UBacteria USpermatoza
## :1 :1 Mode:logical
## 1+ Ca carbonate :0 1+ :0 NA's:1
## 3+ Ca carbonate, Occ Ca oxalate :0 3+ :0
## 3+ calcium carbonate :0 Few :0
## 3+ Calcium carbonate, few calcium oxalate:0 Occasional:0
## Ca carbonate 3+ :0
## (Other) :0
## UFungal UDebris UGram USG UPh UProtein
## :1 :1 Mode:logical Min. : NA :1 :1
## 2+ Hyphae:0 1+:0 NA's:1 1st Qu.: NA >=9.0:0 1+ :0
## 3+:0 Median : NA 6 :0 2+ :0
## Mean :NaN 8 :0 3+ :0
## 3rd Qu.: NA 8.5 :0 Negative:0
## Max. : NA 9 :0
## NA's :1
## UGlucose UKetones UBlood UBilirubin UCultureNote
## :1 :1 :1 :1 :1
## 3+ :0 1+ :0 1+ :0 1+ :0 >10,000 CFU/mL :0
## Negative:0 Negative:0 3+ :0 3+ :0 >100,000 CFU/mL:0
## Trace :0 Trace :0 Negative:0 Negative:0 2,000 CFU/mL :0
## 3,200 CFU/mL :0
## 4,000 CFU/mL :0
## (Other) :0
## M1Leucocytes M1RBC M1Epithelial
## :1 :1 :1
## 1+ :0 1+ :0 1+ :0
## 2+ :0 2+ :0 2+ :0
## 3+ :0 3+ :0 3+ :0
## FEW :0 FEW :0 FEW :0
## NONE SEEN:0 NONE SEEN:0 NONE SEEN:0
## (Other) :0 (Other) :0 (Other) :0
## M1Fungal FMicroForExcess FLeucocytes
## :1 Mode:logical :1
## 2+ fungal hyphae :0 NA's:1 None seen:0
## 2+ Yeast cells and Fungal Hyphae:0
## FEW * :0
## FEW FUNGAL HYPHAE :0
## FUNGAL HYPHAE 1+ :0
## (Other) :0
## FRBC FCampylobacter FYeast FProtozoaCystsOva
## :1 :1 :1 :1
## None seen:0 None seen:0 None seen :0 None seen:0
## Very occasional:0
##
##
##
##
## FTrypsin10 FTrypsin100 FOccultBlood
## Mode:logical Mode:logical Mode:logical
## NA's:1 NA's:1 NA's:1
##
##
##
##
##

sphingomonas <- repro_only[grepl("Sphingomonas|sphingomonas",repro_only$Culture),]
sphingomonas$Culture <- as.factor(as.character(sphingomonas$Culture))
sphingomonas$Specimens <- as.factor(as.character(sphingomonas$Specimens))
summary(sphingomonas$Culture)

## Sphingomonas paucimobilis
## 5

summary(sphingomonas$Specimens)

## Cervix J x 1 Uterine swab-J
## 1 4

summary(sphingomonas)

## ï..Job_Number ClientCode JobDate Species PostCode
## Min. :408415 Min. : 52 1/1/2016 :1 EQU:5 Min. :6258
## 1st Qu.:414657 1st Qu.:168 13/2/2016 :1 1st Qu.:6290
## Median :430057 Median :168 18/10/2016:1 Median :6290
## Mean :437734 Mean :300 22/10/2015:1 Mean :6379
## 3rd Qu.:439312 3rd Qu.:175 24/9/2015 :1 3rd Qu.:6525
## Max. :496228 Max. :937 1/10/2015 :0 Max. :6530
## (Other) :0
## Specimens Prefix Culture Amikacin Amoxycillin
## Cervix J x 1 :1 F :0 Sphingomonas paucimobilis:5 :5 :5
## Uterine swab-J:4 M1:5 R:0 I:0
## U :0 S:0 R:0
## X:0 S:0
## X:0
##
##
## Ampicillin Bactracin Carbenicillin Ceftazidime Ceftiofur Cefuroxime Cephalexin
## :0 :5 :5 :5 :0 :5 :5
## R:1 S:0 R:0 R:0 R:2 S:0 R:0
## S:4 S:0 S:0 S:3 S:0
## X:0 X:0 X:0 X:0 X:0
##
##
##
## Chloramphenicol Ciprofloxacin Clavulox Clavulox_Clavulox Clindamycin
## :5 :5 :5 :5 :5
## R:0 I:0 R:0 S:0 R:0
## S:0 R:0 S:0 S:0
## X:0 S:0 X:0 X:0
##
##
##
## Cloxacillin Doxcycline Doxycycline Doxyycline Enrofloxacin Enrofloxaxin
## :5 :5 :5 :5 :0 :5
## R:0 R:0 I:0 S:0 I:2 R:0
## S:0 S:0 R:0 R:0
## X:0 S:0 S:3
## X:0
##
##
## Erythromycin Fusidic_Acid Gentamicin Lincomycin Marbofloxacin Minocycline
## :5 :5 :0 :5 :5 :5
## R:0 R:0 I:0 R:0 I:0 S:0
## S:0 S:0 R:0 S:0 R:0
## X:0 X:0 S:5 S:0
## X:0
##
##
## Mupirocin Neomycin Norfloxacin Novobiocin Ofloxacin Penicillin Penicillin_G
## :5 :0 :5 :5 :5 :5 :0
## R:0 I:0 R:0 R:0 R:0 R:0 R:2
## S:0 R:1 S:0 S:0 S:0 S:0 S:3
## S:4 X:0 X:0
## X:0
##
##
## Pipercillin Polymyxin_B Rifampicin Soframycin Streptomycin
## :5 :5 :5 :5 Mode:logical
## S:0 R:0 R:0 R:0 NA's:5
## S:0 S:0 S:0
## X:0 X:0 X:0
##
##
##
## Sulphatrimethoprim Tetracycline Ticarcillin Timentin TestDate
## :0 :0 :5 :0 Mode:logical
## R:0 I:0 R:0 I:0 NA's:5
## S:5 R:1 S:0 R:1
## X:0 S:4 X:0 S:4
## X:0 X:0
##
##
## ULeucocytes UEpithelial URBC UCasts
## :5 :5 :5 :5
## <1 :0 <1 :0 <1 :0 None seen:0
## >200 :0 1 :0 >200:0
## 135 :0 18 :0 1 :0
## 2 :0 2 :0 116 :0
## 29 :0 36 :0 3 :0
## (Other):0 (Other):0
## UCrystals UBacteria USpermatoza
## :5 :5 Mode:logical
## 1+ Ca carbonate :0 1+ :0 NA's:5
## 3+ Ca carbonate, Occ Ca oxalate :0 3+ :0
## 3+ calcium carbonate :0 Few :0
## 3+ Calcium carbonate, few calcium oxalate:0 Occasional:0
## Ca carbonate 3+ :0
## (Other) :0
## UFungal UDebris UGram USG UPh UProtein
## :5 :5 Mode:logical Min. : NA :5 :5
## 2+ Hyphae:0 1+:0 NA's:5 1st Qu.: NA >=9.0:0 1+ :0
## 3+:0 Median : NA 6 :0 2+ :0
## Mean :NaN 8 :0 3+ :0
## 3rd Qu.: NA 8.5 :0 Negative:0
## Max. : NA 9 :0
## NA's :5
## UGlucose UKetones UBlood UBilirubin UCultureNote
## :5 :5 :5 :5 :5
## 3+ :0 1+ :0 1+ :0 1+ :0 >10,000 CFU/mL :0
## Negative:0 Negative:0 3+ :0 3+ :0 >100,000 CFU/mL:0
## Trace :0 Trace :0 Negative:0 Negative:0 2,000 CFU/mL :0
## 3,200 CFU/mL :0
## 4,000 CFU/mL :0
## (Other) :0
## M1Leucocytes M1RBC M1Epithelial
## :5 :5 :5
## 1+ :0 1+ :0 1+ :0
## 2+ :0 2+ :0 2+ :0
## 3+ :0 3+ :0 3+ :0
## FEW :0 FEW :0 FEW :0
## NONE SEEN:0 NONE SEEN:0 NONE SEEN:0
## (Other) :0 (Other) :0 (Other) :0
## M1Fungal FMicroForExcess FLeucocytes
## :5 Mode:logical :5
## 2+ fungal hyphae :0 NA's:5 None seen:0
## 2+ Yeast cells and Fungal Hyphae:0
## FEW * :0
## FEW FUNGAL HYPHAE :0
## FUNGAL HYPHAE 1+ :0
## (Other) :0
## FRBC FCampylobacter FYeast FProtozoaCystsOva
## :5 :5 :5 :5
## None seen:0 None seen:0 None seen :0 None seen:0
## Very occasional:0
##
##
##
##
## FTrypsin10 FTrypsin100 FOccultBlood
## Mode:logical Mode:logical Mode:logical
## NA's:5 NA's:5 NA's:5
##
##
##
##
##

citrobacter <- repro_only[grepl("Citrobacter|citrobacter",repro_only$Culture),]
citrobacter$Culture <- as.factor(as.character(citrobacter$Culture))
citrobacter$Specimens <- as.factor(as.character(citrobacter$Specimens))
summary(citrobacter$Culture)

## Citrobacter amalonaticus Citrobacter freundii Citrobacter freundii#
## 1 1 1
## Citrobacter koseri Citrobacter koseri* Citrobacter sp.
## 4 2 1

summary(citrobacter$Specimens)

## culture.plates uterine swab 2 x J Premate swab J
## 1 1
## Premate Swab Sm Pot premate swabJ
## 1 1
## Uterine Lavage H x 1 Uterine swab-J
## 1 4
## Vaginal swab-J
## 1

summary(citrobacter)

## ï..Job_Number ClientCode JobDate Species PostCode
## Min. :400662 Min. : 68.0 1/9/2018 :1 EQU:10 Min. :6069
## 1st Qu.:425794 1st Qu.: 117.8 13/10/2015:1 1st Qu.:6171
## Median :569224 Median : 500.0 16/10/2017:1 Median :6214
## Mean :562198 Mean : 475.4 16/8/2017 :1 Mean :6200
## 3rd Qu.:677411 3rd Qu.: 692.0 17/5/2019 :1 3rd Qu.:6214
## Max. :761753 Max. :1240.0 18/12/2015:1 Max. :6290
## (Other) :4
## Specimens Prefix Culture
## culture.plates uterine swab 2 x J:1 F : 0 Citrobacter amalonaticus:1
## Premate swab J :1 M1:10 Citrobacter freundii :1
## Premate Swab Sm Pot :1 U : 0 Citrobacter freundii# :1
## premate swabJ :1 Citrobacter koseri :4
## Uterine Lavage H x 1 :1 Citrobacter koseri* :2
## Uterine swab-J :4 Citrobacter sp. :1
## Vaginal swab-J :1
## Amikacin Amoxycillin Ampicillin Bactracin Carbenicillin Ceftazidime Ceftiofur
## :10 :10 :0 :10 :10 :10 :0
## R: 0 I: 0 R:9 S: 0 R: 0 R: 0 R:2
## S: 0 R: 0 S:1 S: 0 S: 0 S:8
## X: 0 S: 0 X:0 X: 0 X: 0 X:0
## X: 0
##
##
## Cefuroxime Cephalexin Chloramphenicol Ciprofloxacin Clavulox Clavulox_Clavulox
## :10 :10 :10 :10 :10 :10
## S: 0 R: 0 R: 0 I: 0 R: 0 S: 0
## S: 0 S: 0 R: 0 S: 0
## X: 0 X: 0 S: 0 X: 0
##
##
##
## Clindamycin Cloxacillin Doxcycline Doxycycline Doxyycline Enrofloxacin
## :10 :10 :10 :10 :10 :0
## R: 0 R: 0 R: 0 I: 0 S: 0 I:1
## S: 0 S: 0 S: 0 R: 0 R:0
## X: 0 X: 0 S: 0 S:9
## X: 0
##
##
## Enrofloxaxin Erythromycin Fusidic_Acid Gentamicin Lincomycin Marbofloxacin
## :10 :10 :10 :0 :10 :10
## R: 0 R: 0 R: 0 I:1 R: 0 I: 0
## S: 0 S: 0 R:3 S: 0 R: 0
## X: 0 X: 0 S:6 S: 0
## X:0
##
##
## Minocycline Mupirocin Neomycin Norfloxacin Novobiocin Ofloxacin Penicillin
## :10 :10 :0 :10 :10 :10 :10
## S: 0 R: 0 I:0 R: 0 R: 0 R: 0 R: 0
## S: 0 R:7 S: 0 S: 0 S: 0 S: 0
## S:3 X: 0
## X:0
##
##
## Penicillin_G Pipercillin Polymyxin_B Rifampicin Soframycin Streptomycin
## : 0 :10 :10 :10 :10 Mode:logical
## R:10 S: 0 R: 0 R: 0 R: 0 NA's:10
## S: 0 S: 0 S: 0 S: 0
## X: 0 X: 0 X: 0 X: 0
##
##
##
## Sulphatrimethoprim Tetracycline Ticarcillin Timentin TestDate
## :0 :0 :10 :6 Mode:logical
## R:3 I:0 R: 0 I:0 NA's:10
## S:7 R:3 S: 0 R:1
## X:0 S:7 X: 0 S:3
## X:0 X:0
##
##
## ULeucocytes UEpithelial URBC UCasts
## :10 :10 :10 :10
## <1 : 0 <1 : 0 <1 : 0 None seen: 0
## >200 : 0 1 : 0 >200: 0
## 135 : 0 18 : 0 1 : 0
## 2 : 0 2 : 0 116 : 0
## 29 : 0 36 : 0 3 : 0
## (Other): 0 (Other): 0
## UCrystals UBacteria USpermatoza
## :10 :10 Mode:logical
## 1+ Ca carbonate : 0 1+ : 0 NA's:10
## 3+ Ca carbonate, Occ Ca oxalate : 0 3+ : 0
## 3+ calcium carbonate : 0 Few : 0
## 3+ Calcium carbonate, few calcium oxalate: 0 Occasional: 0
## Ca carbonate 3+ : 0
## (Other) : 0
## UFungal UDebris UGram USG UPh UProtein
## :10 :10 Mode:logical Min. : NA :10 :10
## 2+ Hyphae: 0 1+: 0 NA's:10 1st Qu.: NA >=9.0: 0 1+ : 0
## 3+: 0 Median : NA 6 : 0 2+ : 0
## Mean :NaN 8 : 0 3+ : 0
## 3rd Qu.: NA 8.5 : 0 Negative: 0
## Max. : NA 9 : 0
## NA's :10
## UGlucose UKetones UBlood UBilirubin UCultureNote
## :10 :10 :10 :10 :10
## 3+ : 0 1+ : 0 1+ : 0 1+ : 0 >10,000 CFU/mL : 0
## Negative: 0 Negative: 0 3+ : 0 3+ : 0 >100,000 CFU/mL: 0
## Trace : 0 Trace : 0 Negative: 0 Negative: 0 2,000 CFU/mL : 0
## 3,200 CFU/mL : 0
## 4,000 CFU/mL : 0
## (Other) : 0
## M1Leucocytes M1RBC M1Epithelial
## :10 :10 :10
## 1+ : 0 1+ : 0 1+ : 0
## 2+ : 0 2+ : 0 2+ : 0
## 3+ : 0 3+ : 0 3+ : 0
## FEW : 0 FEW : 0 FEW : 0
## NONE SEEN: 0 NONE SEEN: 0 NONE SEEN: 0
## (Other) : 0 (Other) : 0 (Other) : 0
## M1Fungal FMicroForExcess FLeucocytes
## :10 Mode:logical :10
## 2+ fungal hyphae : 0 NA's:10 None seen: 0
## 2+ Yeast cells and Fungal Hyphae: 0
## FEW * : 0
## FEW FUNGAL HYPHAE : 0
## FUNGAL HYPHAE 1+ : 0
## (Other) : 0
## FRBC FCampylobacter FYeast FProtozoaCystsOva
## :10 :10 :10 :10
## None seen: 0 None seen: 0 None seen : 0 None seen: 0
## Very occasional: 0
##
##
##
##
## FTrypsin10 FTrypsin100 FOccultBlood
## Mode:logical Mode:logical Mode:logical
## NA's:10 NA's:10 NA's:10
##
##
##
##
##

kocuria <- repro_only[grepl("Kocuria|kocuria",repro_only$Culture),]
kocuria$Culture <- as.factor(as.character(kocuria$Culture))
kocuria$Specimens <- as.factor(as.character(kocuria$Specimens))
summary(kocuria$Culture)

## Kocuria kristinae Kocuria kristinae* Kocuria rosea Kocuria rosea*
## 1 2 4 1

summary(kocuria$Specimens)

## clitoral swab -J Placenta Swab Jx 1
## 1 1
## premate swab J x 1 Uterine Fluid H x 1
## 1 1
## Uterine swab-J Uterine swab-J, Smear Q x 1
## 3 1

summary(kocuria)

## ï..Job_Number ClientCode JobDate Species PostCode
## Min. :412045 Min. :101.0 10/10/2015:1 EQU:8 Min. :6055
## 1st Qu.:425532 1st Qu.:265.8 12/12/2015:1 1st Qu.:6069
## Median :609443 Median :308.0 15/11/2018:1 Median :6102
## Mean :571388 Mean :422.9 17/9/2018 :1 Mean :6186
## 3rd Qu.:660596 3rd Qu.:490.0 20/11/2015:1 3rd Qu.:6171
## Max. :758271 Max. :975.0 21/9/2018 :1 Max. :6751
## (Other) :2
## Specimens Prefix Culture Amikacin
## clitoral swab -J :1 F :0 Kocuria kristinae :1 :8
## Placenta Swab Jx 1 :1 M1:8 Kocuria kristinae*:2 R:0
## premate swab J x 1 :1 U :0 Kocuria rosea :4 S:0
## Uterine Fluid H x 1 :1 Kocuria rosea* :1 X:0
## Uterine swab-J :3
## Uterine swab-J, Smear Q x 1:1
##
## Amoxycillin Ampicillin Bactracin Carbenicillin Ceftazidime Ceftiofur
## :7 :1 :8 :8 :8 :0
## I:0 R:3 S:0 R:0 R:0 R:3
## R:1 S:4 S:0 S:0 S:5
## S:0 X:0 X:0 X:0 X:0
## X:0
##
##
## Cefuroxime Cephalexin Chloramphenicol Ciprofloxacin Clavulox Clavulox_Clavulox
## :8 :8 :8 :8 :8 :8
## S:0 R:0 R:0 I:0 R:0 S:0
## S:0 S:0 R:0 S:0
## X:0 X:0 S:0 X:0
##
##
##
## Clindamycin Cloxacillin Doxcycline Doxycycline Doxyycline Enrofloxacin
## :8 :8 :8 :8 :8 :1
## R:0 R:0 R:0 I:0 S:0 I:2
## S:0 S:0 S:0 R:0 R:0
## X:0 X:0 S:0 S:5
## X:0
##
##
## Enrofloxaxin Erythromycin Fusidic_Acid Gentamicin Lincomycin Marbofloxacin
## :8 :7 :8 :0 :8 :8
## R:0 R:0 R:0 I:0 R:0 I:0
## S:1 S:0 R:1 S:0 R:0
## X:0 X:0 S:7 S:0
## X:0
##
##
## Minocycline Mupirocin Neomycin Norfloxacin Novobiocin Ofloxacin Penicillin
## :8 :8 :0 :8 :8 :8 :7
## S:0 R:0 I:0 R:0 R:0 R:0 R:1
## S:0 R:1 S:0 S:0 S:0 S:0
## S:7 X:0
## X:0
##
##
## Penicillin_G Pipercillin Polymyxin_B Rifampicin Soframycin Streptomycin
## :1 :8 :8 :8 :8 Mode:logical
## R:2 S:0 R:0 R:0 R:0 NA's:8
## S:5 S:0 S:0 S:0
## X:0 X:0 X:0 X:0
##
##
##
## Sulphatrimethoprim Tetracycline Ticarcillin Timentin TestDate
## :0 :0 :8 :6 Mode:logical
## R:2 I:0 R:0 I:1 NA's:8
## S:6 R:2 S:0 R:0
## X:0 S:6 X:0 S:1
## X:0 X:0
##
##
## ULeucocytes UEpithelial URBC UCasts
## :8 :8 :8 :8
## <1 :0 <1 :0 <1 :0 None seen:0
## >200 :0 1 :0 >200:0
## 135 :0 18 :0 1 :0
## 2 :0 2 :0 116 :0
## 29 :0 36 :0 3 :0
## (Other):0 (Other):0
## UCrystals UBacteria USpermatoza
## :8 :8 Mode:logical
## 1+ Ca carbonate :0 1+ :0 NA's:8
## 3+ Ca carbonate, Occ Ca oxalate :0 3+ :0
## 3+ calcium carbonate :0 Few :0
## 3+ Calcium carbonate, few calcium oxalate:0 Occasional:0
## Ca carbonate 3+ :0
## (Other) :0
## UFungal UDebris UGram USG UPh UProtein
## :8 :8 Mode:logical Min. : NA :8 :8
## 2+ Hyphae:0 1+:0 NA's:8 1st Qu.: NA >=9.0:0 1+ :0
## 3+:0 Median : NA 6 :0 2+ :0
## Mean :NaN 8 :0 3+ :0
## 3rd Qu.: NA 8.5 :0 Negative:0
## Max. : NA 9 :0
## NA's :8
## UGlucose UKetones UBlood UBilirubin UCultureNote
## :8 :8 :8 :8 :8
## 3+ :0 1+ :0 1+ :0 1+ :0 >10,000 CFU/mL :0
## Negative:0 Negative:0 3+ :0 3+ :0 >100,000 CFU/mL:0
## Trace :0 Trace :0 Negative:0 Negative:0 2,000 CFU/mL :0
## 3,200 CFU/mL :0
## 4,000 CFU/mL :0
## (Other) :0
## M1Leucocytes M1RBC M1Epithelial
## :7 :7 :7
## NONE SEEN:1 VERY OCCASIONAL:1 NONE SEEN:1
## 1+ :0 1+ :0 1+ :0
## 2+ :0 2+ :0 2+ :0
## 3+ :0 3+ :0 3+ :0
## FEW :0 FEW :0 FEW :0
## (Other) :0 (Other) :0 (Other) :0
## M1Fungal FMicroForExcess FLeucocytes
## :7 Mode:logical :8
## NONE SEEN :1 NA's:8 None seen:0
## 2+ fungal hyphae :0
## 2+ Yeast cells and Fungal Hyphae:0
## FEW * :0
## FEW FUNGAL HYPHAE :0
## (Other) :0
## FRBC FCampylobacter FYeast FProtozoaCystsOva
## :8 :8 :8 :8
## None seen:0 None seen:0 None seen :0 None seen:0
## Very occasional:0
##
##
##
##
## FTrypsin10 FTrypsin100 FOccultBlood
## Mode:logical Mode:logical Mode:logical
## NA's:8 NA's:8 NA's:8
##
##
##
##
##

cellulomonas <- repro_only[grepl("Cellulomonas|cellulomonas",repro_only$Culture),]
cellulomonas$Culture <- as.factor(as.character(cellulomonas$Culture))
cellulomonas$Specimens <- as.factor(as.character(cellulomonas$Specimens))
summary(cellulomonas$Culture)

## Cellulomonas spp/Microbacterium spp Cellulomonas spp/Microbacterium spp.
## 1 1
## Cellulomonas spp/Microbacterium spp.*
## 1

summary(cellulomonas$Specimens)

## Uterine swab-J Uterine swab-K Uterine Swab J x 1
## 1 1 1

summary(cellulomonas)

## ï..Job_Number ClientCode JobDate Species PostCode
## Min. :561878 Min. : 692.0 14/8/2017 :1 EQU:3 Min. :6112
## 1st Qu.:589862 1st Qu.: 692.0 23/10/2018:1 1st Qu.:6163
## Median :617845 Median : 692.0 4/4/2018 :1 Median :6214
## Mean :613660 Mean : 838.3 1/1/2016 :0 Mean :6180
## 3rd Qu.:639551 3rd Qu.: 911.5 1/10/2015 :0 3rd Qu.:6214
## Max. :661257 Max. :1131.0 1/10/2019 :0 Max. :6214
## (Other) :0
## Specimens Prefix Culture
## Uterine swab-J :1 F :0 Cellulomonas spp/Microbacterium spp :1
## Uterine swab-K :1 M1:3 Cellulomonas spp/Microbacterium spp. :1
## Uterine Swab J x 1:1 U :0 Cellulomonas spp/Microbacterium spp.*:1
##
##
##
##
## Amikacin Amoxycillin Ampicillin Bactracin Carbenicillin Ceftazidime Ceftiofur
## :3 :3 :0 :3 :3 :3 :0
## R:0 I:0 R:1 S:0 R:0 R:0 R:1
## S:0 R:0 S:2 S:0 S:0 S:2
## X:0 S:0 X:0 X:0 X:0 X:0
## X:0
##
##
## Cefuroxime Cephalexin Chloramphenicol Ciprofloxacin Clavulox Clavulox_Clavulox
## :3 :3 :3 :3 :3 :3
## S:0 R:0 R:0 I:0 R:0 S:0
## S:0 S:0 R:0 S:0
## X:0 X:0 S:0 X:0
##
##
##
## Clindamycin Cloxacillin Doxcycline Doxycycline Doxyycline Enrofloxacin
## :3 :3 :3 :3 :3 :0
## R:0 R:0 R:0 I:0 S:0 I:0
## S:0 S:0 S:0 R:0 R:0
## X:0 X:0 S:0 S:3
## X:0
##
##
## Enrofloxaxin Erythromycin Fusidic_Acid Gentamicin Lincomycin Marbofloxacin
## :3 :3 :3 :0 :3 :3
## R:0 R:0 R:0 I:0 R:0 I:0
## S:0 S:0 R:1 S:0 R:0
## X:0 X:0 S:2 S:0
## X:0
##
##
## Minocycline Mupirocin Neomycin Norfloxacin Novobiocin Ofloxacin Penicillin
## :3 :3 :0 :3 :3 :3 :3
## S:0 R:0 I:0 R:0 R:0 R:0 R:0
## S:0 R:1 S:0 S:0 S:0 S:0
## S:2 X:0
## X:0
##
##
## Penicillin_G Pipercillin Polymyxin_B Rifampicin Soframycin Streptomycin
## :0 :3 :3 :3 :3 Mode:logical
## R:1 S:0 R:0 R:0 R:0 NA's:3
## S:2 S:0 S:0 S:0
## X:0 X:0 X:0 X:0
##
##
##
## Sulphatrimethoprim Tetracycline Ticarcillin Timentin TestDate
## :0 :0 :3 :3 Mode:logical
## R:0 I:0 R:0 I:0 NA's:3
## S:3 R:1 S:0 R:0
## X:0 S:2 X:0 S:0
## X:0 X:0
##
##
## ULeucocytes UEpithelial URBC UCasts
## :3 :3 :3 :3
## <1 :0 <1 :0 <1 :0 None seen:0
## >200 :0 1 :0 >200:0
## 135 :0 18 :0 1 :0
## 2 :0 2 :0 116 :0
## 29 :0 36 :0 3 :0
## (Other):0 (Other):0
## UCrystals UBacteria USpermatoza
## :3 :3 Mode:logical
## 1+ Ca carbonate :0 1+ :0 NA's:3
## 3+ Ca carbonate, Occ Ca oxalate :0 3+ :0
## 3+ calcium carbonate :0 Few :0
## 3+ Calcium carbonate, few calcium oxalate:0 Occasional:0
## Ca carbonate 3+ :0
## (Other) :0
## UFungal UDebris UGram USG UPh UProtein
## :3 :3 Mode:logical Min. : NA :3 :3
## 2+ Hyphae:0 1+:0 NA's:3 1st Qu.: NA >=9.0:0 1+ :0
## 3+:0 Median : NA 6 :0 2+ :0
## Mean :NaN 8 :0 3+ :0
## 3rd Qu.: NA 8.5 :0 Negative:0
## Max. : NA 9 :0
## NA's :3
## UGlucose UKetones UBlood UBilirubin UCultureNote
## :3 :3 :3 :3 :3
## 3+ :0 1+ :0 1+ :0 1+ :0 >10,000 CFU/mL :0
## Negative:0 Negative:0 3+ :0 3+ :0 >100,000 CFU/mL:0
## Trace :0 Trace :0 Negative:0 Negative:0 2,000 CFU/mL :0
## 3,200 CFU/mL :0
## 4,000 CFU/mL :0
## (Other) :0
## M1Leucocytes M1RBC M1Epithelial
## :3 :3 :3
## 1+ :0 1+ :0 1+ :0
## 2+ :0 2+ :0 2+ :0
## 3+ :0 3+ :0 3+ :0
## FEW :0 FEW :0 FEW :0
## NONE SEEN:0 NONE SEEN:0 NONE SEEN:0
## (Other) :0 (Other) :0 (Other) :0
## M1Fungal FMicroForExcess FLeucocytes
## :3 Mode:logical :3
## 2+ fungal hyphae :0 NA's:3 None seen:0
## 2+ Yeast cells and Fungal Hyphae:0
## FEW * :0
## FEW FUNGAL HYPHAE :0
## FUNGAL HYPHAE 1+ :0
## (Other) :0
## FRBC FCampylobacter FYeast FProtozoaCystsOva
## :3 :3 :3 :3
## None seen:0 None seen:0 None seen :0 None seen:0
## Very occasional:0
##
##
##
##
## FTrypsin10 FTrypsin100 FOccultBlood
## Mode:logical Mode:logical Mode:logical
## NA's:3 NA's:3 NA's:3
##
##
##
##
##

proteus <- repro_only[grepl("Proteus|proteus",repro_only$Culture),]
proteus$Culture <- as.factor(as.character(proteus$Culture))
proteus$Specimens <- as.factor(as.character(proteus$Specimens))
summary(proteus$Culture)

## Proteus mirabilis Proteus vulgaris
## 4 1

summary(proteus$Specimens)

## Uterine Fluid BH Uterine lavage-J, Agar media-H
## 1 1
## Uterine swab-J Uterine Swab J x 1
## 1 1
## Vaginal swab-J
## 1

summary(proteus)

## ï..Job_Number ClientCode JobDate Species PostCode
## Min. :409888 Min. : 101.0 1/10/2015 :1 EQU:5 Min. :6121
## 1st Qu.:415721 1st Qu.: 160.0 2/11/2019 :1 1st Qu.:6171
## Median :421690 Median : 301.0 20/11/2015:1 Median :6172
## Mean :551046 Mean : 522.4 27/10/2015:1 Mean :6223
## 3rd Qu.:750569 3rd Qu.:1025.0 4/10/2019 :1 3rd Qu.:6326
## Max. :757360 Max. :1025.0 1/1/2016 :0 Max. :6326
## (Other) :0
## Specimens Prefix Culture Amikacin
## Uterine Fluid BH :1 F :0 Proteus mirabilis:4 :5
## Uterine lavage-J, Agar media-H:1 M1:5 Proteus vulgaris :1 R:0
## Uterine swab-J :1 U :0 S:0
## Uterine Swab J x 1 :1 X:0
## Vaginal swab-J :1
##
##
## Amoxycillin Ampicillin Bactracin Carbenicillin Ceftazidime Ceftiofur
## :5 :0 :5 :5 :5 :0
## I:0 R:3 S:0 R:0 R:0 R:0
## R:0 S:1 S:0 S:0 S:5
## S:0 X:1 X:0 X:0 X:0
## X:0
##
##
## Cefuroxime Cephalexin Chloramphenicol Ciprofloxacin Clavulox Clavulox_Clavulox
## :5 :5 :5 :5 :5 :5
## S:0 R:0 R:0 I:0 R:0 S:0
## S:0 S:0 R:0 S:0
## X:0 X:0 S:0 X:0
##
##
##
## Clindamycin Cloxacillin Doxcycline Doxycycline Doxyycline Enrofloxacin
## :5 :5 :5 :5 :5 :0
## R:0 R:0 R:0 I:0 S:0 I:0
## S:0 S:0 S:0 R:0 R:0
## X:0 X:0 S:0 S:5
## X:0
##
##
## Enrofloxaxin Erythromycin Fusidic_Acid Gentamicin Lincomycin Marbofloxacin
## :5 :5 :5 :0 :5 :5
## R:0 R:0 R:0 I:0 R:0 I:0
## S:0 S:0 R:3 S:0 R:0
## X:0 X:0 S:2 S:0
## X:0
##
##
## Minocycline Mupirocin Neomycin Norfloxacin Novobiocin Ofloxacin Penicillin
## :5 :5 :0 :5 :5 :5 :5
## S:0 R:0 I:0 R:0 R:0 R:0 R:0
## S:0 R:3 S:0 S:0 S:0 S:0
## S:2 X:0
## X:0
##
##
## Penicillin_G Pipercillin Polymyxin_B Rifampicin Soframycin Streptomycin
## :0 :5 :5 :5 :5 Mode:logical
## R:0 S:0 R:0 R:0 R:0 NA's:5
## S:0 S:0 S:0 S:0
## X:5 X:0 X:0 X:0
##
##
##
## Sulphatrimethoprim Tetracycline Ticarcillin Timentin TestDate
## :0 :0 :5 :2 Mode:logical
## R:2 I:0 R:0 I:0 NA's:5
## S:3 R:0 S:0 R:2
## X:0 S:0 X:0 S:1
## X:5 X:0
##
##
## ULeucocytes UEpithelial URBC UCasts
## :5 :5 :5 :5
## <1 :0 <1 :0 <1 :0 None seen:0
## >200 :0 1 :0 >200:0
## 135 :0 18 :0 1 :0
## 2 :0 2 :0 116 :0
## 29 :0 36 :0 3 :0
## (Other):0 (Other):0
## UCrystals UBacteria USpermatoza
## :5 :5 Mode:logical
## 1+ Ca carbonate :0 1+ :0 NA's:5
## 3+ Ca carbonate, Occ Ca oxalate :0 3+ :0
## 3+ calcium carbonate :0 Few :0
## 3+ Calcium carbonate, few calcium oxalate:0 Occasional:0
## Ca carbonate 3+ :0
## (Other) :0
## UFungal UDebris UGram USG UPh UProtein
## :5 :5 Mode:logical Min. : NA :5 :5
## 2+ Hyphae:0 1+:0 NA's:5 1st Qu.: NA >=9.0:0 1+ :0
## 3+:0 Median : NA 6 :0 2+ :0
## Mean :NaN 8 :0 3+ :0
## 3rd Qu.: NA 8.5 :0 Negative:0
## Max. : NA 9 :0
## NA's :5
## UGlucose UKetones UBlood UBilirubin UCultureNote
## :5 :5 :5 :5 :5
## 3+ :0 1+ :0 1+ :0 1+ :0 >10,000 CFU/mL :0
## Negative:0 Negative:0 3+ :0 3+ :0 >100,000 CFU/mL:0
## Trace :0 Trace :0 Negative:0 Negative:0 2,000 CFU/mL :0
## 3,200 CFU/mL :0
## 4,000 CFU/mL :0
## (Other) :0
## M1Leucocytes M1RBC M1Epithelial
## :5 :5 :5
## 1+ :0 1+ :0 1+ :0
## 2+ :0 2+ :0 2+ :0
## 3+ :0 3+ :0 3+ :0
## FEW :0 FEW :0 FEW :0
## NONE SEEN:0 NONE SEEN:0 NONE SEEN:0
## (Other) :0 (Other) :0 (Other) :0
## M1Fungal FMicroForExcess FLeucocytes
## :5 Mode:logical :5
## 2+ fungal hyphae :0 NA's:5 None seen:0
## 2+ Yeast cells and Fungal Hyphae:0
## FEW * :0
## FEW FUNGAL HYPHAE :0
## FUNGAL HYPHAE 1+ :0
## (Other) :0
## FRBC FCampylobacter FYeast FProtozoaCystsOva
## :5 :5 :5 :5
## None seen:0 None seen:0 None seen :0 None seen:0
## Very occasional:0
##
##
##
##
## FTrypsin10 FTrypsin100 FOccultBlood
## Mode:logical Mode:logical Mode:logical
## NA's:5 NA's:5 NA's:5
##
##
##
##
##

lactococcus <- repro_only[grepl("Lactococcus|lactococcus",repro_only$Culture),]
lactococcus$Culture <- as.factor(as.character(lactococcus$Culture))
lactococcus$Specimens <- as.factor(as.character(lactococcus$Specimens))
summary(lactococcus$Culture)

## Lactococcus garvieae
## 2

summary(lactococcus$Specimens)

## Uterine swab-J Vaginal swab-J
## 1 1

summary(lactococcus)

## ï..Job_Number ClientCode JobDate Species PostCode
## Min. :416538 Min. :160 29/10/2015:1 EQU:2 Min. :6069
## 1st Qu.:417027 1st Qu.:197 6/11/2015 :1 1st Qu.:6082
## Median :417517 Median :234 1/1/2016 :0 Median :6095
## Mean :417517 Mean :234 1/10/2015 :0 Mean :6095
## 3rd Qu.:418006 3rd Qu.:271 1/10/2019 :0 3rd Qu.:6108
## Max. :418495 Max. :308 1/11/2016 :0 Max. :6121
## (Other) :0
## Specimens Prefix Culture Amikacin Amoxycillin
## Uterine swab-J:1 F :0 Lactococcus garvieae:2 :2 :2
## Vaginal swab-J:1 M1:2 R:0 I:0
## U :0 S:0 R:0
## X:0 S:0
## X:0
##
##
## Ampicillin Bactracin Carbenicillin Ceftazidime Ceftiofur Cefuroxime Cephalexin
## :0 :2 :2 :2 :0 :2 :2
## R:0 S:0 R:0 R:0 R:0 S:0 R:0
## S:2 S:0 S:0 S:2 S:0
## X:0 X:0 X:0 X:0 X:0
##
##
##
## Chloramphenicol Ciprofloxacin Clavulox Clavulox_Clavulox Clindamycin
## :2 :2 :2 :2 :2
## R:0 I:0 R:0 S:0 R:0
## S:0 R:0 S:0 S:0
## X:0 S:0 X:0 X:0
##
##
##
## Cloxacillin Doxcycline Doxycycline Doxyycline Enrofloxacin Enrofloxaxin
## :2 :2 :2 :2 :0 :2
## R:0 R:0 I:0 S:0 I:1 R:0
## S:0 S:0 R:0 R:0
## X:0 S:0 S:1
## X:0
##
##
## Erythromycin Fusidic_Acid Gentamicin Lincomycin Marbofloxacin Minocycline
## :2 :2 :0 :2 :2 :2
## R:0 R:0 I:0 R:0 I:0 S:0
## S:0 S:0 R:2 S:0 R:0
## X:0 X:0 S:0 S:0
## X:0
##
##
## Mupirocin Neomycin Norfloxacin Novobiocin Ofloxacin Penicillin Penicillin_G
## :2 :0 :2 :2 :2 :2 :0
## R:0 I:0 R:0 R:0 R:0 R:0 R:0
## S:0 R:2 S:0 S:0 S:0 S:0 S:2
## S:0 X:0 X:0
## X:0
##
##
## Pipercillin Polymyxin_B Rifampicin Soframycin Streptomycin
## :2 :2 :2 :2 Mode:logical
## S:0 R:0 R:0 R:0 NA's:2
## S:0 S:0 S:0
## X:0 X:0 X:0
##
##
##
## Sulphatrimethoprim Tetracycline Ticarcillin Timentin TestDate
## :0 :0 :2 :0 Mode:logical
## R:0 I:0 R:0 I:0 NA's:2
## S:2 R:0 S:0 R:0
## X:0 S:2 X:0 S:2
## X:0 X:0
##
##
## ULeucocytes UEpithelial URBC UCasts
## :2 :2 :2 :2
## <1 :0 <1 :0 <1 :0 None seen:0
## >200 :0 1 :0 >200:0
## 135 :0 18 :0 1 :0
## 2 :0 2 :0 116 :0
## 29 :0 36 :0 3 :0
## (Other):0 (Other):0
## UCrystals UBacteria USpermatoza
## :2 :2 Mode:logical
## 1+ Ca carbonate :0 1+ :0 NA's:2
## 3+ Ca carbonate, Occ Ca oxalate :0 3+ :0
## 3+ calcium carbonate :0 Few :0
## 3+ Calcium carbonate, few calcium oxalate:0 Occasional:0
## Ca carbonate 3+ :0
## (Other) :0
## UFungal UDebris UGram USG UPh UProtein
## :2 :2 Mode:logical Min. : NA :2 :2
## 2+ Hyphae:0 1+:0 NA's:2 1st Qu.: NA >=9.0:0 1+ :0
## 3+:0 Median : NA 6 :0 2+ :0
## Mean :NaN 8 :0 3+ :0
## 3rd Qu.: NA 8.5 :0 Negative:0
## Max. : NA 9 :0
## NA's :2
## UGlucose UKetones UBlood UBilirubin UCultureNote
## :2 :2 :2 :2 :2
## 3+ :0 1+ :0 1+ :0 1+ :0 >10,000 CFU/mL :0
## Negative:0 Negative:0 3+ :0 3+ :0 >100,000 CFU/mL:0
## Trace :0 Trace :0 Negative:0 Negative:0 2,000 CFU/mL :0
## 3,200 CFU/mL :0
## 4,000 CFU/mL :0
## (Other) :0
## M1Leucocytes M1RBC M1Epithelial
## :2 :2 :2
## 1+ :0 1+ :0 1+ :0
## 2+ :0 2+ :0 2+ :0
## 3+ :0 3+ :0 3+ :0
## FEW :0 FEW :0 FEW :0
## NONE SEEN:0 NONE SEEN:0 NONE SEEN:0
## (Other) :0 (Other) :0 (Other) :0
## M1Fungal FMicroForExcess FLeucocytes
## :2 Mode:logical :2
## 2+ fungal hyphae :0 NA's:2 None seen:0
## 2+ Yeast cells and Fungal Hyphae:0
## FEW * :0
## FEW FUNGAL HYPHAE :0
## FUNGAL HYPHAE 1+ :0
## (Other) :0
## FRBC FCampylobacter FYeast FProtozoaCystsOva
## :2 :2 :2 :2
## None seen:0 None seen:0 None seen :0 None seen:0
## Very occasional:0
##
##
##
##
## FTrypsin10 FTrypsin100 FOccultBlood
## Mode:logical Mode:logical Mode:logical
## NA's:2 NA's:2 NA's:2
##
##
##
##
##

unidentified <- repro_only[grepl("UNIDENTIFIED|Unidentified|unidentified",repro_only$Culture),]
unidentified$Culture <- as.factor(as.character(unidentified$Culture))
unidentified$Specimens <- as.factor(as.character(unidentified$Specimens))
summary(unidentified$Culture)

## UNIDENTIFIED UNIDENTIFIED# UNIDENTIFIED* UNIDENTIFIED**
## 7 1 1 1

summary(unidentified$Specimens)

## Uterine lavage H x 1 Uterine swab-J
## 1 9

summary(unidentified)

## ï..Job_Number ClientCode JobDate Species PostCode
## Min. :416258 Min. : 84.0 10/11/2018:2 EQU:10 Min. :6084
## 1st Qu.:619626 1st Qu.: 293.0 12/2/2018 :1 1st Qu.:6176
## Median :677205 Median : 692.0 23/10/2018:1 Median :6214
## Mean :657179 Mean : 637.9 28/10/2015:1 Mean :6262
## 3rd Qu.:727852 3rd Qu.: 860.0 29/11/2017:1 3rd Qu.:6315
## Max. :764476 Max. :1240.0 3/12/2019 :1 Max. :6503
## (Other) :3
## Specimens Prefix Culture Amikacin Amoxycillin
## Uterine lavage H x 1:1 F : 0 UNIDENTIFIED :7 :10 :10
## Uterine swab-J :9 M1:10 UNIDENTIFIED# :1 R: 0 I: 0
## U : 0 UNIDENTIFIED* :1 S: 0 R: 0
## UNIDENTIFIED**:1 X: 0 S: 0
## X: 0
##
##
## Ampicillin Bactracin Carbenicillin Ceftazidime Ceftiofur Cefuroxime Cephalexin
## :1 :10 :10 :10 :1 :10 :10
## R:1 S: 0 R: 0 R: 0 R:3 S: 0 R: 0
## S:8 S: 0 S: 0 S:6 S: 0
## X:0 X: 0 X: 0 X:0 X: 0
##
##
##
## Chloramphenicol Ciprofloxacin Clavulox Clavulox_Clavulox Clindamycin
## :10 :10 :10 :10 :10
## R: 0 I: 0 R: 0 S: 0 R: 0
## S: 0 R: 0 S: 0 S: 0
## X: 0 S: 0 X: 0 X: 0
##
##
##
## Cloxacillin Doxcycline Doxycycline Doxyycline Enrofloxacin Enrofloxaxin
## :10 :10 :10 :10 :1 :10
## R: 0 R: 0 I: 0 S: 0 I:2 R: 0
## S: 0 S: 0 R: 0 R:1
## X: 0 S: 0 S:6
## X: 0
##
##
## Erythromycin Fusidic_Acid Gentamicin Lincomycin Marbofloxacin Minocycline
## :10 :10 :1 :10 :10 :10
## R: 0 R: 0 I:0 R: 0 I: 0 S: 0
## S: 0 S: 0 R:2 S: 0 R: 0
## X: 0 X: 0 S:7 S: 0
## X:0
##
##
## Mupirocin Neomycin Norfloxacin Novobiocin Ofloxacin Penicillin Penicillin_G
## :10 :1 :10 :10 :10 :10 :1
## R: 0 I:0 R: 0 R: 0 R: 0 R: 0 R:2
## S: 0 R:3 S: 0 S: 0 S: 0 S: 0 S:7
## S:6 X: 0 X:0
## X:0
##
##
## Pipercillin Polymyxin_B Rifampicin Soframycin Streptomycin
## :10 :10 :10 :10 Mode:logical
## S: 0 R: 0 R: 0 R: 0 NA's:10
## S: 0 S: 0 S: 0
## X: 0 X: 0 X: 0
##
##
##
## Sulphatrimethoprim Tetracycline Ticarcillin Timentin TestDate
## :1 :1 :10 :9 Mode:logical
## R:2 I:0 R: 0 I:0 NA's:10
## S:7 R:2 S: 0 R:0
## X:0 S:7 X: 0 S:1
## X:0 X:0
##
##
## ULeucocytes UEpithelial URBC UCasts
## :10 :10 :10 :10
## <1 : 0 <1 : 0 <1 : 0 None seen: 0
## >200 : 0 1 : 0 >200: 0
## 135 : 0 18 : 0 1 : 0
## 2 : 0 2 : 0 116 : 0
## 29 : 0 36 : 0 3 : 0
## (Other): 0 (Other): 0
## UCrystals UBacteria USpermatoza
## :10 :10 Mode:logical
## 1+ Ca carbonate : 0 1+ : 0 NA's:10
## 3+ Ca carbonate, Occ Ca oxalate : 0 3+ : 0
## 3+ calcium carbonate : 0 Few : 0
## 3+ Calcium carbonate, few calcium oxalate: 0 Occasional: 0
## Ca carbonate 3+ : 0
## (Other) : 0
## UFungal UDebris UGram USG UPh UProtein
## :10 :10 Mode:logical Min. : NA :10 :10
## 2+ Hyphae: 0 1+: 0 NA's:10 1st Qu.: NA >=9.0: 0 1+ : 0
## 3+: 0 Median : NA 6 : 0 2+ : 0
## Mean :NaN 8 : 0 3+ : 0
## 3rd Qu.: NA 8.5 : 0 Negative: 0
## Max. : NA 9 : 0
## NA's :10
## UGlucose UKetones UBlood UBilirubin UCultureNote
## :10 :10 :10 :10 :10
## 3+ : 0 1+ : 0 1+ : 0 1+ : 0 >10,000 CFU/mL : 0
## Negative: 0 Negative: 0 3+ : 0 3+ : 0 >100,000 CFU/mL: 0
## Trace : 0 Trace : 0 Negative: 0 Negative: 0 2,000 CFU/mL : 0
## 3,200 CFU/mL : 0
## 4,000 CFU/mL : 0
## (Other) : 0
## M1Leucocytes M1RBC M1Epithelial
## :10 :10 :10
## 1+ : 0 1+ : 0 1+ : 0
## 2+ : 0 2+ : 0 2+ : 0
## 3+ : 0 3+ : 0 3+ : 0
## FEW : 0 FEW : 0 FEW : 0
## NONE SEEN: 0 NONE SEEN: 0 NONE SEEN: 0
## (Other) : 0 (Other) : 0 (Other) : 0
## M1Fungal FMicroForExcess FLeucocytes
## :10 Mode:logical :10
## 2+ fungal hyphae : 0 NA's:10 None seen: 0
## 2+ Yeast cells and Fungal Hyphae: 0
## FEW * : 0
## FEW FUNGAL HYPHAE : 0
## FUNGAL HYPHAE 1+ : 0
## (Other) : 0
## FRBC FCampylobacter FYeast FProtozoaCystsOva
## :10 :10 :10 :10
## None seen: 0 None seen: 0 None seen : 0 None seen: 0
## Very occasional: 0
##
##
##
##
## FTrypsin10 FTrypsin100 FOccultBlood
## Mode:logical Mode:logical Mode:logical
## NA's:10 NA's:10 NA's:10
##
##
##
##
##

aeromonas <- repro_only[grepl("Aeromonas|aeromonas",repro_only$Culture),]
aeromonas$Culture <- as.factor(as.character(aeromonas$Culture))
aeromonas$Specimens <- as.factor(as.character(aeromonas$Specimens))
summary(aeromonas$Culture)

## Aeromonas media Aeromonas sobria Aeromonas sp.*
## 1 1 1

summary(aeromonas$Specimens)

## Uterine flush H x 1 Uterine lavage H x 1 Uterine swab-J
## 1 1 1

summary(aeromonas)

## ï..Job_Number ClientCode JobDate Species PostCode
## Min. :417974 Min. : 168.0 15/11/2018:1 EQU:3 Min. :6163
## 1st Qu.:503024 1st Qu.: 704.0 23/11/2017:1 1st Qu.:6163
## Median :588074 Median :1240.0 5/11/2015 :1 Median :6163
## Mean :561637 Mean : 882.7 1/1/2016 :0 Mean :6205
## 3rd Qu.:633469 3rd Qu.:1240.0 1/10/2015 :0 3rd Qu.:6226
## Max. :678864 Max. :1240.0 1/10/2019 :0 Max. :6290
## (Other) :0
## Specimens Prefix Culture Amikacin Amoxycillin
## Uterine flush H x 1 :1 F :0 Aeromonas media :1 :3 :3
## Uterine lavage H x 1:1 M1:3 Aeromonas sobria:1 R:0 I:0
## Uterine swab-J :1 U :0 Aeromonas sp.* :1 S:0 R:0
## X:0 S:0
## X:0
##
##
## Ampicillin Bactracin Carbenicillin Ceftazidime Ceftiofur Cefuroxime Cephalexin
## :0 :3 :3 :3 :0 :3 :3
## R:3 S:0 R:0 R:0 R:0 S:0 R:0
## S:0 S:0 S:0 S:3 S:0
## X:0 X:0 X:0 X:0 X:0
##
##
##
## Chloramphenicol Ciprofloxacin Clavulox Clavulox_Clavulox Clindamycin
## :3 :3 :3 :3 :3
## R:0 I:0 R:0 S:0 R:0
## S:0 R:0 S:0 S:0
## X:0 S:0 X:0 X:0
##
##
##
## Cloxacillin Doxcycline Doxycycline Doxyycline Enrofloxacin Enrofloxaxin
## :3 :3 :3 :3 :0 :3
## R:0 R:0 I:0 S:0 I:1 R:0
## S:0 S:0 R:0 R:0
## X:0 S:0 S:2
## X:0
##
##
## Erythromycin Fusidic_Acid Gentamicin Lincomycin Marbofloxacin Minocycline
## :3 :3 :0 :3 :3 :3
## R:0 R:0 I:0 R:0 I:0 S:0
## S:0 S:0 R:1 S:0 R:0
## X:0 X:0 S:2 S:0
## X:0
##
##
## Mupirocin Neomycin Norfloxacin Novobiocin Ofloxacin Penicillin Penicillin_G
## :3 :0 :3 :3 :3 :3 :0
## R:0 I:0 R:0 R:0 R:0 R:0 R:3
## S:0 R:2 S:0 S:0 S:0 S:0 S:0
## S:1 X:0 X:0
## X:0
##
##
## Pipercillin Polymyxin_B Rifampicin Soframycin Streptomycin
## :3 :3 :3 :3 Mode:logical
## S:0 R:0 R:0 R:0 NA's:3
## S:0 S:0 S:0
## X:0 X:0 X:0
##
##
##
## Sulphatrimethoprim Tetracycline Ticarcillin Timentin TestDate
## :0 :0 :3 :2 Mode:logical
## R:2 I:0 R:0 I:0 NA's:3
## S:1 R:0 S:0 R:0
## X:0 S:3 X:0 S:1
## X:0 X:0
##
##
## ULeucocytes UEpithelial URBC UCasts
## :3 :3 :3 :3
## <1 :0 <1 :0 <1 :0 None seen:0
## >200 :0 1 :0 >200:0
## 135 :0 18 :0 1 :0
## 2 :0 2 :0 116 :0
## 29 :0 36 :0 3 :0
## (Other):0 (Other):0
## UCrystals UBacteria USpermatoza
## :3 :3 Mode:logical
## 1+ Ca carbonate :0 1+ :0 NA's:3
## 3+ Ca carbonate, Occ Ca oxalate :0 3+ :0
## 3+ calcium carbonate :0 Few :0
## 3+ Calcium carbonate, few calcium oxalate:0 Occasional:0
## Ca carbonate 3+ :0
## (Other) :0
## UFungal UDebris UGram USG UPh UProtein
## :3 :3 Mode:logical Min. : NA :3 :3
## 2+ Hyphae:0 1+:0 NA's:3 1st Qu.: NA >=9.0:0 1+ :0
## 3+:0 Median : NA 6 :0 2+ :0
## Mean :NaN 8 :0 3+ :0
## 3rd Qu.: NA 8.5 :0 Negative:0
## Max. : NA 9 :0
## NA's :3
## UGlucose UKetones UBlood UBilirubin UCultureNote
## :3 :3 :3 :3 :3
## 3+ :0 1+ :0 1+ :0 1+ :0 >10,000 CFU/mL :0
## Negative:0 Negative:0 3+ :0 3+ :0 >100,000 CFU/mL:0
## Trace :0 Trace :0 Negative:0 Negative:0 2,000 CFU/mL :0
## 3,200 CFU/mL :0
## 4,000 CFU/mL :0
## (Other) :0
## M1Leucocytes M1RBC M1Epithelial
## :3 :3 :3
## 1+ :0 1+ :0 1+ :0
## 2+ :0 2+ :0 2+ :0
## 3+ :0 3+ :0 3+ :0
## FEW :0 FEW :0 FEW :0
## NONE SEEN:0 NONE SEEN:0 NONE SEEN:0
## (Other) :0 (Other) :0 (Other) :0
## M1Fungal FMicroForExcess FLeucocytes
## :3 Mode:logical :3
## 2+ fungal hyphae :0 NA's:3 None seen:0
## 2+ Yeast cells and Fungal Hyphae:0
## FEW * :0
## FEW FUNGAL HYPHAE :0
## FUNGAL HYPHAE 1+ :0
## (Other) :0
## FRBC FCampylobacter FYeast FProtozoaCystsOva
## :3 :3 :3 :3
## None seen:0 None seen:0 None seen :0 None seen:0
## Very occasional:0
##
##
##
##
## FTrypsin10 FTrypsin100 FOccultBlood
## Mode:logical Mode:logical Mode:logical
## NA's:3 NA's:3 NA's:3
##
##
##
##
##

lefsonia <- repro_only[grepl("Lefsonia|lefsonia",repro_only$Culture),]
lefsonia$Culture <- as.factor(as.character(lefsonia$Culture))
lefsonia$Specimens <- as.factor(as.character(lefsonia$Specimens))
summary(lefsonia$Culture)

## Lefsonia aquatica
## 1

summary(lefsonia$Specimens)

## Uterine swab-J
## 1

summary(lefsonia)

## ï..Job_Number ClientCode JobDate Species PostCode
## Min. :426703 Min. :692 11/12/2015:1 EQU:1 Min. :6214
## 1st Qu.:426703 1st Qu.:692 1/1/2016 :0 1st Qu.:6214
## Median :426703 Median :692 1/10/2015 :0 Median :6214
## Mean :426703 Mean :692 1/10/2019 :0 Mean :6214
## 3rd Qu.:426703 3rd Qu.:692 1/11/2016 :0 3rd Qu.:6214
## Max. :426703 Max. :692 1/11/2017 :0 Max. :6214
## (Other) :0
## Specimens Prefix Culture Amikacin Amoxycillin
## Uterine swab-J:1 F :0 Lefsonia aquatica:1 :1 :1
## M1:1 R:0 I:0
## U :0 S:0 R:0
## X:0 S:0
## X:0
##
##
## Ampicillin Bactracin Carbenicillin Ceftazidime Ceftiofur Cefuroxime Cephalexin
## :0 :1 :1 :1 :0 :1 :1
## R:0 S:0 R:0 R:0 R:0 S:0 R:0
## S:1 S:0 S:0 S:1 S:0
## X:0 X:0 X:0 X:0 X:0
##
##
##
## Chloramphenicol Ciprofloxacin Clavulox Clavulox_Clavulox Clindamycin
## :1 :1 :1 :1 :1
## R:0 I:0 R:0 S:0 R:0
## S:0 R:0 S:0 S:0
## X:0 S:0 X:0 X:0
##
##
##
## Cloxacillin Doxcycline Doxycycline Doxyycline Enrofloxacin Enrofloxaxin
## :1 :1 :1 :1 :0 :1
## R:0 R:0 I:0 S:0 I:0 R:0
## S:0 S:0 R:0 R:0
## X:0 S:0 S:1
## X:0
##
##
## Erythromycin Fusidic_Acid Gentamicin Lincomycin Marbofloxacin Minocycline
## :1 :1 :0 :1 :1 :1
## R:0 R:0 I:0 R:0 I:0 S:0
## S:0 S:0 R:1 S:0 R:0
## X:0 X:0 S:0 S:0
## X:0
##
##
## Mupirocin Neomycin Norfloxacin Novobiocin Ofloxacin Penicillin Penicillin_G
## :1 :0 :1 :1 :1 :1 :0
## R:0 I:0 R:0 R:0 R:0 R:0 R:0
## S:0 R:0 S:0 S:0 S:0 S:0 S:1
## S:1 X:0 X:0
## X:0
##
##
## Pipercillin Polymyxin_B Rifampicin Soframycin Streptomycin
## :1 :1 :1 :1 Mode:logical
## S:0 R:0 R:0 R:0 NA's:1
## S:0 S:0 S:0
## X:0 X:0 X:0
##
##
##
## Sulphatrimethoprim Tetracycline Ticarcillin Timentin TestDate
## :0 :0 :1 :0 Mode:logical
## R:0 I:0 R:0 I:0 NA's:1
## S:1 R:0 S:0 R:0
## X:0 S:1 X:0 S:1
## X:0 X:0
##
##
## ULeucocytes UEpithelial URBC UCasts
## :1 :1 :1 :1
## <1 :0 <1 :0 <1 :0 None seen:0
## >200 :0 1 :0 >200:0
## 135 :0 18 :0 1 :0
## 2 :0 2 :0 116 :0
## 29 :0 36 :0 3 :0
## (Other):0 (Other):0
## UCrystals UBacteria USpermatoza
## :1 :1 Mode:logical
## 1+ Ca carbonate :0 1+ :0 NA's:1
## 3+ Ca carbonate, Occ Ca oxalate :0 3+ :0
## 3+ calcium carbonate :0 Few :0
## 3+ Calcium carbonate, few calcium oxalate:0 Occasional:0
## Ca carbonate 3+ :0
## (Other) :0
## UFungal UDebris UGram USG UPh UProtein
## :1 :1 Mode:logical Min. : NA :1 :1
## 2+ Hyphae:0 1+:0 NA's:1 1st Qu.: NA >=9.0:0 1+ :0
## 3+:0 Median : NA 6 :0 2+ :0
## Mean :NaN 8 :0 3+ :0
## 3rd Qu.: NA 8.5 :0 Negative:0
## Max. : NA 9 :0
## NA's :1
## UGlucose UKetones UBlood UBilirubin UCultureNote
## :1 :1 :1 :1 :1
## 3+ :0 1+ :0 1+ :0 1+ :0 >10,000 CFU/mL :0
## Negative:0 Negative:0 3+ :0 3+ :0 >100,000 CFU/mL:0
## Trace :0 Trace :0 Negative:0 Negative:0 2,000 CFU/mL :0
## 3,200 CFU/mL :0
## 4,000 CFU/mL :0
## (Other) :0
## M1Leucocytes M1RBC M1Epithelial
## :1 :1 :1
## 1+ :0 1+ :0 1+ :0
## 2+ :0 2+ :0 2+ :0
## 3+ :0 3+ :0 3+ :0
## FEW :0 FEW :0 FEW :0
## NONE SEEN:0 NONE SEEN:0 NONE SEEN:0
## (Other) :0 (Other) :0 (Other) :0
## M1Fungal FMicroForExcess FLeucocytes
## :1 Mode:logical :1
## 2+ fungal hyphae :0 NA's:1 None seen:0
## 2+ Yeast cells and Fungal Hyphae:0
## FEW * :0
## FEW FUNGAL HYPHAE :0
## FUNGAL HYPHAE 1+ :0
## (Other) :0
## FRBC FCampylobacter FYeast FProtozoaCystsOva
## :1 :1 :1 :1
## None seen:0 None seen:0 None seen :0 None seen:0
## Very occasional:0
##
##
##
##
## FTrypsin10 FTrypsin100 FOccultBlood
## Mode:logical Mode:logical Mode:logical
## NA's:1 NA's:1 NA's:1
##
##
##
##
##

bordetella <- repro_only[grepl("Bordetella|bordetella",repro_only$Culture),]
bordetella$Culture <- as.factor(as.character(bordetella$Culture))
bordetella$Specimens <- as.factor(as.character(bordetella$Specimens))
summary(bordetella$Culture)

## Bordetella bronchiseptica Bordetella bronchiseptica*
## 1 1
## Bordetella sp.
## 1

summary(bordetella$Specimens)

## Eq Uterine Swab J x 1 Uterine swab-J
## 1 2

summary(bordetella)

## ï..Job_Number ClientCode JobDate Species PostCode
## Min. :426745 Min. : 160.0 1/9/2018 :1 EQU:3 Min. :6121
## 1st Qu.:468671 1st Qu.: 592.5 12/12/2015:1 1st Qu.:6224
## Median :510596 Median :1025.0 19/12/2016:1 Median :6326
## Mean :529130 Mean : 736.7 1/1/2016 :0 Mean :6258
## 3rd Qu.:580322 3rd Qu.:1025.0 1/10/2015 :0 3rd Qu.:6326
## Max. :650048 Max. :1025.0 1/10/2019 :0 Max. :6326
## (Other) :0
## Specimens Prefix Culture Amikacin
## Eq Uterine Swab J x 1:1 F :0 Bordetella bronchiseptica :1 :3
## Uterine swab-J :2 M1:3 Bordetella bronchiseptica*:1 R:0
## U :0 Bordetella sp. :1 S:0
## X:0
##
##
##
## Amoxycillin Ampicillin Bactracin Carbenicillin Ceftazidime Ceftiofur
## :3 :0 :3 :3 :3 :0
## I:0 R:2 S:0 R:0 R:0 R:3
## R:0 S:1 S:0 S:0 S:0
## S:0 X:0 X:0 X:0 X:0
## X:0
##
##
## Cefuroxime Cephalexin Chloramphenicol Ciprofloxacin Clavulox Clavulox_Clavulox
## :3 :3 :3 :3 :3 :3
## S:0 R:0 R:0 I:0 R:0 S:0
## S:0 S:0 R:0 S:0
## X:0 X:0 S:0 X:0
##
##
##
## Clindamycin Cloxacillin Doxcycline Doxycycline Doxyycline Enrofloxacin
## :3 :3 :3 :3 :3 :0
## R:0 R:0 R:0 I:0 S:0 I:0
## S:0 S:0 S:0 R:0 R:0
## X:0 X:0 S:0 S:3
## X:0
##
##
## Enrofloxaxin Erythromycin Fusidic_Acid Gentamicin Lincomycin Marbofloxacin
## :3 :3 :3 :0 :3 :3
## R:0 R:0 R:0 I:0 R:0 I:0
## S:0 S:0 R:0 S:0 R:0
## X:0 X:0 S:3 S:0
## X:0
##
##
## Minocycline Mupirocin Neomycin Norfloxacin Novobiocin Ofloxacin Penicillin
## :3 :3 :0 :3 :3 :3 :3
## S:0 R:0 I:0 R:0 R:0 R:0 R:0
## S:0 R:0 S:0 S:0 S:0 S:0
## S:3 X:0
## X:0
##
##
## Penicillin_G Pipercillin Polymyxin_B Rifampicin Soframycin Streptomycin
## :0 :3 :3 :3 :3 Mode:logical
## R:3 S:0 R:0 R:0 R:0 NA's:3
## S:0 S:0 S:0 S:0
## X:0 X:0 X:0 X:0
##
##
##
## Sulphatrimethoprim Tetracycline Ticarcillin Timentin TestDate
## :0 :0 :3 :1 Mode:logical
## R:0 I:0 R:0 I:0 NA's:3
## S:3 R:0 S:0 R:0
## X:0 S:3 X:0 S:2
## X:0 X:0
##
##
## ULeucocytes UEpithelial URBC UCasts
## :3 :3 :3 :3
## <1 :0 <1 :0 <1 :0 None seen:0
## >200 :0 1 :0 >200:0
## 135 :0 18 :0 1 :0
## 2 :0 2 :0 116 :0
## 29 :0 36 :0 3 :0
## (Other):0 (Other):0
## UCrystals UBacteria USpermatoza
## :3 :3 Mode:logical
## 1+ Ca carbonate :0 1+ :0 NA's:3
## 3+ Ca carbonate, Occ Ca oxalate :0 3+ :0
## 3+ calcium carbonate :0 Few :0
## 3+ Calcium carbonate, few calcium oxalate:0 Occasional:0
## Ca carbonate 3+ :0
## (Other) :0
## UFungal UDebris UGram USG UPh UProtein
## :3 :3 Mode:logical Min. : NA :3 :3
## 2+ Hyphae:0 1+:0 NA's:3 1st Qu.: NA >=9.0:0 1+ :0
## 3+:0 Median : NA 6 :0 2+ :0
## Mean :NaN 8 :0 3+ :0
## 3rd Qu.: NA 8.5 :0 Negative:0
## Max. : NA 9 :0
## NA's :3
## UGlucose UKetones UBlood UBilirubin UCultureNote
## :3 :3 :3 :3 :3
## 3+ :0 1+ :0 1+ :0 1+ :0 >10,000 CFU/mL :0
## Negative:0 Negative:0 3+ :0 3+ :0 >100,000 CFU/mL:0
## Trace :0 Trace :0 Negative:0 Negative:0 2,000 CFU/mL :0
## 3,200 CFU/mL :0
## 4,000 CFU/mL :0
## (Other) :0
## M1Leucocytes M1RBC M1Epithelial
## :3 :3 :3
## 1+ :0 1+ :0 1+ :0
## 2+ :0 2+ :0 2+ :0
## 3+ :0 3+ :0 3+ :0
## FEW :0 FEW :0 FEW :0
## NONE SEEN:0 NONE SEEN:0 NONE SEEN:0
## (Other) :0 (Other) :0 (Other) :0
## M1Fungal FMicroForExcess FLeucocytes
## :3 Mode:logical :3
## 2+ fungal hyphae :0 NA's:3 None seen:0
## 2+ Yeast cells and Fungal Hyphae:0
## FEW * :0
## FEW FUNGAL HYPHAE :0
## FUNGAL HYPHAE 1+ :0
## (Other) :0
## FRBC FCampylobacter FYeast FProtozoaCystsOva
## :3 :3 :3 :3
## None seen:0 None seen:0 None seen :0 None seen:0
## Very occasional:0
##
##
##
##
## FTrypsin10 FTrypsin100 FOccultBlood
## Mode:logical Mode:logical Mode:logical
## NA's:3 NA's:3 NA's:3
##
##
##
##
##

actinomyces <- repro_only[grepl("Actinomyces|actinomyces",repro_only$Culture),]
actinomyces$Culture <- as.factor(as.character(actinomyces$Culture))
actinomyces$Specimens <- as.factor(as.character(actinomyces$Specimens))
summary(actinomyces$Culture)

## Actinomyces species
## 2

summary(actinomyces$Specimens)

## Uterine Fluid H x 1 , add 16/11 Uterine swab-J
## 1 1

summary(actinomyces)

## ï..Job_Number ClientCode JobDate Species PostCode
## Min. :420050 Min. :865.0 13/11/2015:1 EQU:2 Min. :6055
## 1st Qu.:423613 1st Qu.:892.5 20/1/2016 :1 1st Qu.:6062
## Median :427176 Median :920.0 1/1/2016 :0 Median :6070
## Mean :427176 Mean :920.0 1/10/2015 :0 Mean :6070
## 3rd Qu.:430739 3rd Qu.:947.5 1/10/2019 :0 3rd Qu.:6077
## Max. :434302 Max. :975.0 1/11/2016 :0 Max. :6084
## (Other) :0
## Specimens Prefix Culture Amikacin
## Uterine Fluid H x 1 , add 16/11:1 F :0 Actinomyces species:2 :2
## Uterine swab-J :1 M1:2 R:0
## U :0 S:0
## X:0
##
##
##
## Amoxycillin Ampicillin Bactracin Carbenicillin Ceftazidime Ceftiofur
## :2 :0 :2 :2 :2 :0
## I:0 R:0 S:0 R:0 R:0 R:0
## R:0 S:2 S:0 S:0 S:2
## S:0 X:0 X:0 X:0 X:0
## X:0
##
##
## Cefuroxime Cephalexin Chloramphenicol Ciprofloxacin Clavulox Clavulox_Clavulox
## :2 :2 :2 :2 :2 :2
## S:0 R:0 R:0 I:0 R:0 S:0
## S:0 S:0 R:0 S:0
## X:0 X:0 S:0 X:0
##
##
##
## Clindamycin Cloxacillin Doxcycline Doxycycline Doxyycline Enrofloxacin
## :2 :2 :2 :2 :2 :0
## R:0 R:0 R:0 I:0 S:0 I:0
## S:0 S:0 S:0 R:0 R:0
## X:0 X:0 S:0 S:2
## X:0
##
##
## Enrofloxaxin Erythromycin Fusidic_Acid Gentamicin Lincomycin Marbofloxacin
## :2 :2 :2 :0 :2 :2
## R:0 R:0 R:0 I:0 R:0 I:0
## S:0 S:0 R:0 S:0 R:0
## X:0 X:0 S:2 S:0
## X:0
##
##
## Minocycline Mupirocin Neomycin Norfloxacin Novobiocin Ofloxacin Penicillin
## :2 :2 :0 :2 :2 :2 :2
## S:0 R:0 I:0 R:0 R:0 R:0 R:0
## S:0 R:0 S:0 S:0 S:0 S:0
## S:2 X:0
## X:0
##
##
## Penicillin_G Pipercillin Polymyxin_B Rifampicin Soframycin Streptomycin
## :0 :2 :2 :2 :2 Mode:logical
## R:0 S:0 R:0 R:0 R:0 NA's:2
## S:2 S:0 S:0 S:0
## X:0 X:0 X:0 X:0
##
##
##
## Sulphatrimethoprim Tetracycline Ticarcillin Timentin TestDate
## :0 :0 :2 :0 Mode:logical
## R:0 I:0 R:0 I:0 NA's:2
## S:2 R:0 S:0 R:0
## X:0 S:2 X:0 S:2
## X:0 X:0
##
##
## ULeucocytes UEpithelial URBC UCasts
## :2 :2 :2 :2
## <1 :0 <1 :0 <1 :0 None seen:0
## >200 :0 1 :0 >200:0
## 135 :0 18 :0 1 :0
## 2 :0 2 :0 116 :0
## 29 :0 36 :0 3 :0
## (Other):0 (Other):0
## UCrystals UBacteria USpermatoza
## :2 :2 Mode:logical
## 1+ Ca carbonate :0 1+ :0 NA's:2
## 3+ Ca carbonate, Occ Ca oxalate :0 3+ :0
## 3+ calcium carbonate :0 Few :0
## 3+ Calcium carbonate, few calcium oxalate:0 Occasional:0
## Ca carbonate 3+ :0
## (Other) :0
## UFungal UDebris UGram USG UPh UProtein
## :2 :2 Mode:logical Min. : NA :2 :2
## 2+ Hyphae:0 1+:0 NA's:2 1st Qu.: NA >=9.0:0 1+ :0
## 3+:0 Median : NA 6 :0 2+ :0
## Mean :NaN 8 :0 3+ :0
## 3rd Qu.: NA 8.5 :0 Negative:0
## Max. : NA 9 :0
## NA's :2
## UGlucose UKetones UBlood UBilirubin UCultureNote
## :2 :2 :2 :2 :2
## 3+ :0 1+ :0 1+ :0 1+ :0 >10,000 CFU/mL :0
## Negative:0 Negative:0 3+ :0 3+ :0 >100,000 CFU/mL:0
## Trace :0 Trace :0 Negative:0 Negative:0 2,000 CFU/mL :0
## 3,200 CFU/mL :0
## 4,000 CFU/mL :0
## (Other) :0
## M1Leucocytes M1RBC M1Epithelial
## :2 :2 :2
## 1+ :0 1+ :0 1+ :0
## 2+ :0 2+ :0 2+ :0
## 3+ :0 3+ :0 3+ :0
## FEW :0 FEW :0 FEW :0
## NONE SEEN:0 NONE SEEN:0 NONE SEEN:0
## (Other) :0 (Other) :0 (Other) :0
## M1Fungal FMicroForExcess FLeucocytes
## :2 Mode:logical :2
## 2+ fungal hyphae :0 NA's:2 None seen:0
## 2+ Yeast cells and Fungal Hyphae:0
## FEW * :0
## FEW FUNGAL HYPHAE :0
## FUNGAL HYPHAE 1+ :0
## (Other) :0
## FRBC FCampylobacter FYeast FProtozoaCystsOva
## :2 :2 :2 :2
## None seen:0 None seen:0 None seen :0 None seen:0
## Very occasional:0
##
##
##
##
## FTrypsin10 FTrypsin100 FOccultBlood
## Mode:logical Mode:logical Mode:logical
## NA's:2 NA's:2 NA's:2
##
##
##
##
##

escherichia <- repro_only[grepl("Escherichia|escherichia",repro_only$Culture),]
escherichia$Culture <- as.factor(as.character(escherichia$Culture))
escherichia$Specimens <- as.factor(as.character(escherichia$Specimens))
summary(escherichia$Culture)

## Escherichia hermannii Escherichia hermannii* Escherichia vulneris
## 1 1 1

summary(escherichia$Specimens)

## premate swab J x 1 Uterine swab-J
## 1 1
## Uterine swab-J, Smear Q x 1
## 1

summary(escherichia)

## ï..Job_Number ClientCode JobDate Species PostCode
## Min. :455300 Min. : 365.0 13/3/2019:1 EQU:3 Min. :6112
## 1st Qu.:554897 1st Qu.: 528.5 21/9/2018:1 1st Qu.:6163
## Median :654493 Median : 692.0 29/4/2016:1 Median :6214
## Mean :604862 Mean : 729.3 1/1/2016 :0 Mean :6359
## 3rd Qu.:679643 3rd Qu.: 911.5 1/10/2015:0 3rd Qu.:6482
## Max. :704792 Max. :1131.0 1/10/2019:0 Max. :6751
## (Other) :0
## Specimens Prefix Culture Amikacin
## premate swab J x 1 :1 F :0 Escherichia hermannii :1 :3
## Uterine swab-J :1 M1:3 Escherichia hermannii*:1 R:0
## Uterine swab-J, Smear Q x 1:1 U :0 Escherichia vulneris :1 S:0
## X:0
##
##
##
## Amoxycillin Ampicillin Bactracin Carbenicillin Ceftazidime Ceftiofur
## :2 :1 :3 :3 :3 :0
## I:0 R:2 S:0 R:0 R:0 R:0
## R:1 S:0 S:0 S:0 S:3
## S:0 X:0 X:0 X:0 X:0
## X:0
##
##
## Cefuroxime Cephalexin Chloramphenicol Ciprofloxacin Clavulox Clavulox_Clavulox
## :3 :3 :3 :3 :3 :3
## S:0 R:0 R:0 I:0 R:0 S:0
## S:0 S:0 R:0 S:0
## X:0 X:0 S:0 X:0
##
##
##
## Clindamycin Cloxacillin Doxcycline Doxycycline Doxyycline Enrofloxacin
## :3 :3 :3 :3 :3 :1
## R:0 R:0 R:0 I:0 S:0 I:0
## S:0 S:0 S:0 R:0 R:0
## X:0 X:0 S:0 S:2
## X:0
##
##
## Enrofloxaxin Erythromycin Fusidic_Acid Gentamicin Lincomycin Marbofloxacin
## :3 :2 :3 :0 :3 :3
## R:0 R:1 R:0 I:0 R:0 I:0
## S:0 S:0 R:0 S:0 R:0
## X:0 X:0 S:3 S:0
## X:0
##
##
## Minocycline Mupirocin Neomycin Norfloxacin Novobiocin Ofloxacin Penicillin
## :3 :3 :0 :3 :3 :3 :2
## S:0 R:0 I:0 R:0 R:0 R:0 R:1
## S:0 R:1 S:0 S:0 S:0 S:0
## S:2 X:0
## X:0
##
##
## Penicillin_G Pipercillin Polymyxin_B Rifampicin Soframycin Streptomycin
## :1 :3 :3 :3 :3 Mode:logical
## R:2 S:0 R:0 R:0 R:0 NA's:3
## S:0 S:0 S:0 S:0
## X:0 X:0 X:0 X:0
##
##
##
## Sulphatrimethoprim Tetracycline Ticarcillin Timentin TestDate
## :0 :0 :3 :3 Mode:logical
## R:0 I:0 R:0 I:0 NA's:3
## S:3 R:0 S:0 R:0
## X:0 S:3 X:0 S:0
## X:0 X:0
##
##
## ULeucocytes UEpithelial URBC UCasts
## :3 :3 :3 :3
## <1 :0 <1 :0 <1 :0 None seen:0
## >200 :0 1 :0 >200:0
## 135 :0 18 :0 1 :0
## 2 :0 2 :0 116 :0
## 29 :0 36 :0 3 :0
## (Other):0 (Other):0
## UCrystals UBacteria USpermatoza
## :3 :3 Mode:logical
## 1+ Ca carbonate :0 1+ :0 NA's:3
## 3+ Ca carbonate, Occ Ca oxalate :0 3+ :0
## 3+ calcium carbonate :0 Few :0
## 3+ Calcium carbonate, few calcium oxalate:0 Occasional:0
## Ca carbonate 3+ :0
## (Other) :0
## UFungal UDebris UGram USG UPh UProtein
## :3 :3 Mode:logical Min. : NA :3 :3
## 2+ Hyphae:0 1+:0 NA's:3 1st Qu.: NA >=9.0:0 1+ :0
## 3+:0 Median : NA 6 :0 2+ :0
## Mean :NaN 8 :0 3+ :0
## 3rd Qu.: NA 8.5 :0 Negative:0
## Max. : NA 9 :0
## NA's :3
## UGlucose UKetones UBlood UBilirubin UCultureNote
## :3 :3 :3 :3 :3
## 3+ :0 1+ :0 1+ :0 1+ :0 >10,000 CFU/mL :0
## Negative:0 Negative:0 3+ :0 3+ :0 >100,000 CFU/mL:0
## Trace :0 Trace :0 Negative:0 Negative:0 2,000 CFU/mL :0
## 3,200 CFU/mL :0
## 4,000 CFU/mL :0
## (Other) :0
## M1Leucocytes M1RBC M1Epithelial
## :3 :3 :3
## 1+ :0 1+ :0 1+ :0
## 2+ :0 2+ :0 2+ :0
## 3+ :0 3+ :0 3+ :0
## FEW :0 FEW :0 FEW :0
## NONE SEEN:0 NONE SEEN:0 NONE SEEN:0
## (Other) :0 (Other) :0 (Other) :0
## M1Fungal FMicroForExcess FLeucocytes
## :3 Mode:logical :3
## 2+ fungal hyphae :0 NA's:3 None seen:0
## 2+ Yeast cells and Fungal Hyphae:0
## FEW * :0
## FEW FUNGAL HYPHAE :0
## FUNGAL HYPHAE 1+ :0
## (Other) :0
## FRBC FCampylobacter FYeast FProtozoaCystsOva
## :3 :3 :3 :3
## None seen:0 None seen:0 None seen :0 None seen:0
## Very occasional:0
##
##
##
##
## FTrypsin10 FTrypsin100 FOccultBlood
## Mode:logical Mode:logical Mode:logical
## NA's:3 NA's:3 NA's:3
##
##
##
##
##

Achromobacter <- repro_only[grepl("Achromobacter|achromobacter",repro_only$Culture),]
Achromobacter$Culture <- as.factor(as.character(Achromobacter$Culture))
Achromobacter$Specimens <- as.factor(as.character(Achromobacter$Specimens))
summary(Achromobacter$Culture)

## Achromobacter denitrificans* Achromobacter sp.*
## 1 1
## Achromobacter xylosoxidans
## 1

summary(Achromobacter$Specimens)

## 1 x premate swab PM swab J x 1
## 2 1

summary(Achromobacter)

## ï..Job_Number ClientCode JobDate Species PostCode
## Min. :724526 Min. : 23.0 13/6/2019:2 EQU:3 Min. :6214
## 1st Qu.:724526 1st Qu.: 23.0 28/8/2019:1 1st Qu.:6370
## Median :724526 Median : 23.0 1/1/2016 :0 Median :6525
## Mean :730365 Mean :246.0 1/10/2015:0 Mean :6421
## 3rd Qu.:733284 3rd Qu.:357.5 1/10/2019:0 3rd Qu.:6525
## Max. :742042 Max. :692.0 1/11/2016:0 Max. :6525
## (Other) :0
## Specimens Prefix Culture Amikacin
## 1 x premate swab:2 F :0 Achromobacter denitrificans*:1 :3
## PM swab J x 1 :1 M1:3 Achromobacter sp.* :1 R:0
## U :0 Achromobacter xylosoxidans :1 S:0
## X:0
##
##
##
## Amoxycillin Ampicillin Bactracin Carbenicillin Ceftazidime Ceftiofur
## :3 :0 :3 :3 :3 :0
## I:0 R:1 S:0 R:0 R:0 R:3
## R:0 S:2 S:0 S:0 S:0
## S:0 X:0 X:0 X:0 X:0
## X:0
##
##
## Cefuroxime Cephalexin Chloramphenicol Ciprofloxacin Clavulox Clavulox_Clavulox
## :3 :3 :3 :3 :3 :3
## S:0 R:0 R:0 I:0 R:0 S:0
## S:0 S:0 R:0 S:0
## X:0 X:0 S:0 X:0
##
##
##
## Clindamycin Cloxacillin Doxcycline Doxycycline Doxyycline Enrofloxacin
## :3 :3 :3 :3 :3 :0
## R:0 R:0 R:0 I:0 S:0 I:1
## S:0 S:0 S:0 R:0 R:2
## X:0 X:0 S:0 S:0
## X:0
##
##
## Enrofloxaxin Erythromycin Fusidic_Acid Gentamicin Lincomycin Marbofloxacin
## :3 :3 :3 :0 :3 :3
## R:0 R:0 R:0 I:0 R:0 I:0
## S:0 S:0 R:2 S:0 R:0
## X:0 X:0 S:1 S:0
## X:0
##
##
## Minocycline Mupirocin Neomycin Norfloxacin Novobiocin Ofloxacin Penicillin
## :3 :3 :0 :3 :3 :3 :3
## S:0 R:0 I:0 R:0 R:0 R:0 R:0
## S:0 R:2 S:0 S:0 S:0 S:0
## S:1 X:0
## X:0
##
##
## Penicillin_G Pipercillin Polymyxin_B Rifampicin Soframycin Streptomycin
## :0 :3 :3 :3 :3 Mode:logical
## R:3 S:0 R:0 R:0 R:0 NA's:3
## S:0 S:0 S:0 S:0
## X:0 X:0 X:0 X:0
##
##
##
## Sulphatrimethoprim Tetracycline Ticarcillin Timentin TestDate
## :0 :0 :3 :3 Mode:logical
## R:0 I:0 R:0 I:0 NA's:3
## S:3 R:2 S:0 R:0
## X:0 S:1 X:0 S:0
## X:0 X:0
##
##
## ULeucocytes UEpithelial URBC UCasts
## :3 :3 :3 :3
## <1 :0 <1 :0 <1 :0 None seen:0
## >200 :0 1 :0 >200:0
## 135 :0 18 :0 1 :0
## 2 :0 2 :0 116 :0
## 29 :0 36 :0 3 :0
## (Other):0 (Other):0
## UCrystals UBacteria USpermatoza
## :3 :3 Mode:logical
## 1+ Ca carbonate :0 1+ :0 NA's:3
## 3+ Ca carbonate, Occ Ca oxalate :0 3+ :0
## 3+ calcium carbonate :0 Few :0
## 3+ Calcium carbonate, few calcium oxalate:0 Occasional:0
## Ca carbonate 3+ :0
## (Other) :0
## UFungal UDebris UGram USG UPh UProtein
## :3 :3 Mode:logical Min. : NA :3 :3
## 2+ Hyphae:0 1+:0 NA's:3 1st Qu.: NA >=9.0:0 1+ :0
## 3+:0 Median : NA 6 :0 2+ :0
## Mean :NaN 8 :0 3+ :0
## 3rd Qu.: NA 8.5 :0 Negative:0
## Max. : NA 9 :0
## NA's :3
## UGlucose UKetones UBlood UBilirubin UCultureNote
## :3 :3 :3 :3 :3
## 3+ :0 1+ :0 1+ :0 1+ :0 >10,000 CFU/mL :0
## Negative:0 Negative:0 3+ :0 3+ :0 >100,000 CFU/mL:0
## Trace :0 Trace :0 Negative:0 Negative:0 2,000 CFU/mL :0
## 3,200 CFU/mL :0
## 4,000 CFU/mL :0
## (Other) :0
## M1Leucocytes M1RBC M1Epithelial
## :3 :3 :3
## 1+ :0 1+ :0 1+ :0
## 2+ :0 2+ :0 2+ :0
## 3+ :0 3+ :0 3+ :0
## FEW :0 FEW :0 FEW :0
## NONE SEEN:0 NONE SEEN:0 NONE SEEN:0
## (Other) :0 (Other) :0 (Other) :0
## M1Fungal FMicroForExcess FLeucocytes
## :3 Mode:logical :3
## 2+ fungal hyphae :0 NA's:3 None seen:0
## 2+ Yeast cells and Fungal Hyphae:0
## FEW * :0
## FEW FUNGAL HYPHAE :0
## FUNGAL HYPHAE 1+ :0
## (Other) :0
## FRBC FCampylobacter FYeast FProtozoaCystsOva
## :3 :3 :3 :3
## None seen:0 None seen:0 None seen :0 None seen:0
## Very occasional:0
##
##
##
##
## FTrypsin10 FTrypsin100 FOccultBlood
## Mode:logical Mode:logical Mode:logical
## NA's:3 NA's:3 NA's:3
##
##
##
##
##

Actinobacillus <- repro_only[grepl("Actinobacillus|actinobacillus",repro_only$Culture),]
Actinobacillus$Culture <- as.factor(as.character(Actinobacillus$Culture))
Actinobacillus$Specimens <- as.factor(as.character(Actinobacillus$Specimens))
summary(Actinobacillus$Culture)

## Actinobacillus sp. Actinobacillus suis/equuli
## 1 1

summary(Actinobacillus$Specimens)

## Uterine Fluid H x 1 Uterine swab-J
## 1 1

summary(Actinobacillus)

## ï..Job_Number ClientCode JobDate Species PostCode
## Min. :419451 Min. :160.0 11/11/2015:1 EQU:2 Min. :6084
## 1st Qu.:509735 1st Qu.:336.2 14/2/2020 :1 1st Qu.:6093
## Median :600019 Median :512.5 1/1/2016 :0 Median :6102
## Mean :600019 Mean :512.5 1/10/2015 :0 Mean :6102
## 3rd Qu.:690302 3rd Qu.:688.8 1/10/2019 :0 3rd Qu.:6112
## Max. :780586 Max. :865.0 1/11/2016 :0 Max. :6121
## (Other) :0
## Specimens Prefix Culture Amikacin
## Uterine Fluid H x 1:1 F :0 Actinobacillus sp. :1 :2
## Uterine swab-J :1 M1:2 Actinobacillus suis/equuli:1 R:0
## U :0 S:0
## X:0
##
##
##
## Amoxycillin Ampicillin Bactracin Carbenicillin Ceftazidime Ceftiofur
## :2 :0 :2 :2 :2 :0
## I:0 R:0 S:0 R:0 R:0 R:0
## R:0 S:2 S:0 S:0 S:2
## S:0 X:0 X:0 X:0 X:0
## X:0
##
##
## Cefuroxime Cephalexin Chloramphenicol Ciprofloxacin Clavulox Clavulox_Clavulox
## :2 :2 :2 :2 :2 :2
## S:0 R:0 R:0 I:0 R:0 S:0
## S:0 S:0 R:0 S:0
## X:0 X:0 S:0 X:0
##
##
##
## Clindamycin Cloxacillin Doxcycline Doxycycline Doxyycline Enrofloxacin
## :2 :2 :2 :2 :2 :0
## R:0 R:0 R:0 I:0 S:0 I:0
## S:0 S:0 S:0 R:0 R:0
## X:0 X:0 S:0 S:2
## X:0
##
##
## Enrofloxaxin Erythromycin Fusidic_Acid Gentamicin Lincomycin Marbofloxacin
## :2 :2 :2 :0 :2 :2
## R:0 R:0 R:0 I:0 R:0 I:0
## S:0 S:0 R:1 S:0 R:0
## X:0 X:0 S:1 S:0
## X:0
##
##
## Minocycline Mupirocin Neomycin Norfloxacin Novobiocin Ofloxacin Penicillin
## :2 :2 :0 :2 :2 :2 :2
## S:0 R:0 I:0 R:0 R:0 R:0 R:0
## S:0 R:2 S:0 S:0 S:0 S:0
## S:0 X:0
## X:0
##
##
## Penicillin_G Pipercillin Polymyxin_B Rifampicin Soframycin Streptomycin
## :0 :2 :2 :2 :2 Mode:logical
## R:2 S:0 R:0 R:0 R:0 NA's:2
## S:0 S:0 S:0 S:0
## X:0 X:0 X:0 X:0
##
##
##
## Sulphatrimethoprim Tetracycline Ticarcillin Timentin TestDate
## :0 :0 :2 :1 Mode:logical
## R:0 I:0 R:0 I:0 NA's:2
## S:2 R:0 S:0 R:0
## X:0 S:2 X:0 S:1
## X:0 X:0
##
##
## ULeucocytes UEpithelial URBC UCasts
## :2 :2 :2 :2
## <1 :0 <1 :0 <1 :0 None seen:0
## >200 :0 1 :0 >200:0
## 135 :0 18 :0 1 :0
## 2 :0 2 :0 116 :0
## 29 :0 36 :0 3 :0
## (Other):0 (Other):0
## UCrystals UBacteria USpermatoza
## :2 :2 Mode:logical
## 1+ Ca carbonate :0 1+ :0 NA's:2
## 3+ Ca carbonate, Occ Ca oxalate :0 3+ :0
## 3+ calcium carbonate :0 Few :0
## 3+ Calcium carbonate, few calcium oxalate:0 Occasional:0
## Ca carbonate 3+ :0
## (Other) :0
## UFungal UDebris UGram USG UPh UProtein
## :2 :2 Mode:logical Min. : NA :2 :2
## 2+ Hyphae:0 1+:0 NA's:2 1st Qu.: NA >=9.0:0 1+ :0
## 3+:0 Median : NA 6 :0 2+ :0
## Mean :NaN 8 :0 3+ :0
## 3rd Qu.: NA 8.5 :0 Negative:0
## Max. : NA 9 :0
## NA's :2
## UGlucose UKetones UBlood UBilirubin UCultureNote
## :2 :2 :2 :2 :2
## 3+ :0 1+ :0 1+ :0 1+ :0 >10,000 CFU/mL :0
## Negative:0 Negative:0 3+ :0 3+ :0 >100,000 CFU/mL:0
## Trace :0 Trace :0 Negative:0 Negative:0 2,000 CFU/mL :0
## 3,200 CFU/mL :0
## 4,000 CFU/mL :0
## (Other) :0
## M1Leucocytes M1RBC M1Epithelial
## :2 :2 :2
## 1+ :0 1+ :0 1+ :0
## 2+ :0 2+ :0 2+ :0
## 3+ :0 3+ :0 3+ :0
## FEW :0 FEW :0 FEW :0
## NONE SEEN:0 NONE SEEN:0 NONE SEEN:0
## (Other) :0 (Other) :0 (Other) :0
## M1Fungal FMicroForExcess FLeucocytes
## :2 Mode:logical :2
## 2+ fungal hyphae :0 NA's:2 None seen:0
## 2+ Yeast cells and Fungal Hyphae:0
## FEW * :0
## FEW FUNGAL HYPHAE :0
## FUNGAL HYPHAE 1+ :0
## (Other) :0
## FRBC FCampylobacter FYeast FProtozoaCystsOva
## :2 :2 :2 :2
## None seen:0 None seen:0 None seen :0 None seen:0
## Very occasional:0
##
##
##
##
## FTrypsin10 FTrypsin100 FOccultBlood
## Mode:logical Mode:logical Mode:logical
## NA's:2 NA's:2 NA's:2
##
##
##
##
##

brevibacterium <- repro_only[grepl("Brevibacterium|brevibacterium",repro_only$Culture),]
brevibacterium$Culture <- as.factor(as.character(brevibacterium$Culture))
brevibacterium$Specimens <- as.factor(as.character(brevibacterium$Specimens))
summary(brevibacterium$Culture)

## Brevibacterium spp. ##
## 1

summary(brevibacterium$Specimens)

## Uterine swab-J
## 1

summary(brevibacterium)

## ï..Job_Number ClientCode JobDate Species PostCode
## Min. :495686 Min. :807 14/10/2016:1 EQU:1 Min. :6931
## 1st Qu.:495686 1st Qu.:807 1/1/2016 :0 1st Qu.:6931
## Median :495686 Median :807 1/10/2015 :0 Median :6931
## Mean :495686 Mean :807 1/10/2019 :0 Mean :6931
## 3rd Qu.:495686 3rd Qu.:807 1/11/2016 :0 3rd Qu.:6931
## Max. :495686 Max. :807 1/11/2017 :0 Max. :6931
## (Other) :0
## Specimens Prefix Culture Amikacin Amoxycillin
## Uterine swab-J:1 F :0 Brevibacterium spp. ##:1 :1 :1
## M1:1 R:0 I:0
## U :0 S:0 R:0
## X:0 S:0
## X:0
##
##
## Ampicillin Bactracin Carbenicillin Ceftazidime Ceftiofur Cefuroxime Cephalexin
## :0 :1 :1 :1 :0 :1 :1
## R:0 S:0 R:0 R:0 R:0 S:0 R:0
## S:1 S:0 S:0 S:1 S:0
## X:0 X:0 X:0 X:0 X:0
##
##
##
## Chloramphenicol Ciprofloxacin Clavulox Clavulox_Clavulox Clindamycin
## :1 :1 :1 :1 :1
## R:0 I:0 R:0 S:0 R:0
## S:0 R:0 S:0 S:0
## X:0 S:0 X:0 X:0
##
##
##
## Cloxacillin Doxcycline Doxycycline Doxyycline Enrofloxacin Enrofloxaxin
## :1 :1 :1 :1 :0 :1
## R:0 R:0 I:0 S:0 I:0 R:0
## S:0 S:0 R:0 R:0
## X:0 S:0 S:1
## X:0
##
##
## Erythromycin Fusidic_Acid Gentamicin Lincomycin Marbofloxacin Minocycline
## :1 :1 :0 :1 :1 :1
## R:0 R:0 I:0 R:0 I:0 S:0
## S:0 S:0 R:0 S:0 R:0
## X:0 X:0 S:1 S:0
## X:0
##
##
## Mupirocin Neomycin Norfloxacin Novobiocin Ofloxacin Penicillin Penicillin_G
## :1 :0 :1 :1 :1 :1 :0
## R:0 I:0 R:0 R:0 R:0 R:0 R:0
## S:0 R:0 S:0 S:0 S:0 S:0 S:1
## S:1 X:0 X:0
## X:0
##
##
## Pipercillin Polymyxin_B Rifampicin Soframycin Streptomycin
## :1 :1 :1 :1 Mode:logical
## S:0 R:0 R:0 R:0 NA's:1
## S:0 S:0 S:0
## X:0 X:0 X:0
##
##
##
## Sulphatrimethoprim Tetracycline Ticarcillin Timentin TestDate
## :0 :0 :1 :0 Mode:logical
## R:0 I:0 R:0 I:0 NA's:1
## S:1 R:0 S:0 R:0
## X:0 S:1 X:0 S:1
## X:0 X:0
##
##
## ULeucocytes UEpithelial URBC UCasts
## :1 :1 :1 :1
## <1 :0 <1 :0 <1 :0 None seen:0
## >200 :0 1 :0 >200:0
## 135 :0 18 :0 1 :0
## 2 :0 2 :0 116 :0
## 29 :0 36 :0 3 :0
## (Other):0 (Other):0
## UCrystals UBacteria USpermatoza
## :1 :1 Mode:logical
## 1+ Ca carbonate :0 1+ :0 NA's:1
## 3+ Ca carbonate, Occ Ca oxalate :0 3+ :0
## 3+ calcium carbonate :0 Few :0
## 3+ Calcium carbonate, few calcium oxalate:0 Occasional:0
## Ca carbonate 3+ :0
## (Other) :0
## UFungal UDebris UGram USG UPh UProtein
## :1 :1 Mode:logical Min. : NA :1 :1
## 2+ Hyphae:0 1+:0 NA's:1 1st Qu.: NA >=9.0:0 1+ :0
## 3+:0 Median : NA 6 :0 2+ :0
## Mean :NaN 8 :0 3+ :0
## 3rd Qu.: NA 8.5 :0 Negative:0
## Max. : NA 9 :0
## NA's :1
## UGlucose UKetones UBlood UBilirubin UCultureNote
## :1 :1 :1 :1 :1
## 3+ :0 1+ :0 1+ :0 1+ :0 >10,000 CFU/mL :0
## Negative:0 Negative:0 3+ :0 3+ :0 >100,000 CFU/mL:0
## Trace :0 Trace :0 Negative:0 Negative:0 2,000 CFU/mL :0
## 3,200 CFU/mL :0
## 4,000 CFU/mL :0
## (Other) :0
## M1Leucocytes M1RBC M1Epithelial
## :1 :1 :1
## 1+ :0 1+ :0 1+ :0
## 2+ :0 2+ :0 2+ :0
## 3+ :0 3+ :0 3+ :0
## FEW :0 FEW :0 FEW :0
## NONE SEEN:0 NONE SEEN:0 NONE SEEN:0
## (Other) :0 (Other) :0 (Other) :0
## M1Fungal FMicroForExcess FLeucocytes
## :1 Mode:logical :1
## 2+ fungal hyphae :0 NA's:1 None seen:0
## 2+ Yeast cells and Fungal Hyphae:0
## FEW * :0
## FEW FUNGAL HYPHAE :0
## FUNGAL HYPHAE 1+ :0
## (Other) :0
## FRBC FCampylobacter FYeast FProtozoaCystsOva
## :1 :1 :1 :1
## None seen:0 None seen:0 None seen :0 None seen:0
## Very occasional:0
##
##
##
##
## FTrypsin10 FTrypsin100 FOccultBlood
## Mode:logical Mode:logical Mode:logical
## NA's:1 NA's:1 NA's:1
##
##
##
##
##

arcanobacterium <- repro_only[grepl("Arcanobacterium|arcanobacterium",repro_only$Culture),]
arcanobacterium$Culture <- as.factor(as.character(arcanobacterium$Culture))
arcanobacterium$Specimens <- as.factor(as.character(arcanobacterium$Specimens))
summary(arcanobacterium$Culture)

## Arcanobacterium haemolyticum
## 1

summary(arcanobacterium$Specimens)

## Uterine swab-J
## 1

summary(arcanobacterium)

## ï..Job_Number ClientCode JobDate Species PostCode
## Min. :500486 Min. :149 5/11/2016:1 EQU:1 Min. :6280
## 1st Qu.:500486 1st Qu.:149 1/1/2016 :0 1st Qu.:6280
## Median :500486 Median :149 1/10/2015:0 Median :6280
## Mean :500486 Mean :149 1/10/2019:0 Mean :6280
## 3rd Qu.:500486 3rd Qu.:149 1/11/2016:0 3rd Qu.:6280
## Max. :500486 Max. :149 1/11/2017:0 Max. :6280
## (Other) :0
## Specimens Prefix Culture Amikacin
## Uterine swab-J:1 F :0 Arcanobacterium haemolyticum:1 :1
## M1:1 R:0
## U :0 S:0
## X:0
##
##
##
## Amoxycillin Ampicillin Bactracin Carbenicillin Ceftazidime Ceftiofur
## :1 :0 :1 :1 :1 :0
## I:0 R:0 S:0 R:0 R:0 R:0
## R:0 S:1 S:0 S:0 S:1
## S:0 X:0 X:0 X:0 X:0
## X:0
##
##
## Cefuroxime Cephalexin Chloramphenicol Ciprofloxacin Clavulox Clavulox_Clavulox
## :1 :1 :1 :1 :1 :1
## S:0 R:0 R:0 I:0 R:0 S:0
## S:0 S:0 R:0 S:0
## X:0 X:0 S:0 X:0
##
##
##
## Clindamycin Cloxacillin Doxcycline Doxycycline Doxyycline Enrofloxacin
## :1 :1 :1 :1 :1 :0
## R:0 R:0 R:0 I:0 S:0 I:0
## S:0 S:0 S:0 R:0 R:0
## X:0 X:0 S:0 S:1
## X:0
##
##
## Enrofloxaxin Erythromycin Fusidic_Acid Gentamicin Lincomycin Marbofloxacin
## :1 :1 :1 :0 :1 :1
## R:0 R:0 R:0 I:0 R:0 I:0
## S:0 S:0 R:0 S:0 R:0
## X:0 X:0 S:1 S:0
## X:0
##
##
## Minocycline Mupirocin Neomycin Norfloxacin Novobiocin Ofloxacin Penicillin
## :1 :1 :0 :1 :1 :1 :1
## S:0 R:0 I:0 R:0 R:0 R:0 R:0
## S:0 R:0 S:0 S:0 S:0 S:0
## S:1 X:0
## X:0
##
##
## Penicillin_G Pipercillin Polymyxin_B Rifampicin Soframycin Streptomycin
## :0 :1 :1 :1 :1 Mode:logical
## R:0 S:0 R:0 R:0 R:0 NA's:1
## S:1 S:0 S:0 S:0
## X:0 X:0 X:0 X:0
##
##
##
## Sulphatrimethoprim Tetracycline Ticarcillin Timentin TestDate
## :0 :0 :1 :0 Mode:logical
## R:0 I:0 R:0 I:0 NA's:1
## S:1 R:0 S:0 R:0
## X:0 S:1 X:0 S:1
## X:0 X:0
##
##
## ULeucocytes UEpithelial URBC UCasts
## :1 :1 :1 :1
## <1 :0 <1 :0 <1 :0 None seen:0
## >200 :0 1 :0 >200:0
## 135 :0 18 :0 1 :0
## 2 :0 2 :0 116 :0
## 29 :0 36 :0 3 :0
## (Other):0 (Other):0
## UCrystals UBacteria USpermatoza
## :1 :1 Mode:logical
## 1+ Ca carbonate :0 1+ :0 NA's:1
## 3+ Ca carbonate, Occ Ca oxalate :0 3+ :0
## 3+ calcium carbonate :0 Few :0
## 3+ Calcium carbonate, few calcium oxalate:0 Occasional:0
## Ca carbonate 3+ :0
## (Other) :0
## UFungal UDebris UGram USG UPh UProtein
## :1 :1 Mode:logical Min. : NA :1 :1
## 2+ Hyphae:0 1+:0 NA's:1 1st Qu.: NA >=9.0:0 1+ :0
## 3+:0 Median : NA 6 :0 2+ :0
## Mean :NaN 8 :0 3+ :0
## 3rd Qu.: NA 8.5 :0 Negative:0
## Max. : NA 9 :0
## NA's :1
## UGlucose UKetones UBlood UBilirubin UCultureNote
## :1 :1 :1 :1 :1
## 3+ :0 1+ :0 1+ :0 1+ :0 >10,000 CFU/mL :0
## Negative:0 Negative:0 3+ :0 3+ :0 >100,000 CFU/mL:0
## Trace :0 Trace :0 Negative:0 Negative:0 2,000 CFU/mL :0
## 3,200 CFU/mL :0
## 4,000 CFU/mL :0
## (Other) :0
## M1Leucocytes M1RBC M1Epithelial
## :1 :1 :1
## 1+ :0 1+ :0 1+ :0
## 2+ :0 2+ :0 2+ :0
## 3+ :0 3+ :0 3+ :0
## FEW :0 FEW :0 FEW :0
## NONE SEEN:0 NONE SEEN:0 NONE SEEN:0
## (Other) :0 (Other) :0 (Other) :0
## M1Fungal FMicroForExcess FLeucocytes
## :1 Mode:logical :1
## 2+ fungal hyphae :0 NA's:1 None seen:0
## 2+ Yeast cells and Fungal Hyphae:0
## FEW * :0
## FEW FUNGAL HYPHAE :0
## FUNGAL HYPHAE 1+ :0
## (Other) :0
## FRBC FCampylobacter FYeast FProtozoaCystsOva
## :1 :1 :1 :1
## None seen:0 None seen:0 None seen :0 None seen:0
## Very occasional:0
##
##
##
##
## FTrypsin10 FTrypsin100 FOccultBlood
## Mode:logical Mode:logical Mode:logical
## NA's:1 NA's:1 NA's:1
##
##
##
##
##

trueperella <- repro_only[grepl("Trueperella|trueperella",repro_only$Culture),]
trueperella$Culture <- as.factor(as.character(trueperella$Culture))
trueperella$Specimens <- as.factor(as.character(trueperella$Specimens))
summary(trueperella$Culture)

## Trueperella pyogenes
## 1

summary(trueperella$Specimens)

## Uterine swab-J
## 1

summary(trueperella)

## ï..Job_Number ClientCode JobDate Species PostCode
## Min. :502698 Min. :101 15/11/2016:1 EQU:1 Min. :6171
## 1st Qu.:502698 1st Qu.:101 1/1/2016 :0 1st Qu.:6171
## Median :502698 Median :101 1/10/2015 :0 Median :6171
## Mean :502698 Mean :101 1/10/2019 :0 Mean :6171
## 3rd Qu.:502698 3rd Qu.:101 1/11/2016 :0 3rd Qu.:6171
## Max. :502698 Max. :101 1/11/2017 :0 Max. :6171
## (Other) :0
## Specimens Prefix Culture Amikacin Amoxycillin
## Uterine swab-J:1 F :0 Trueperella pyogenes:1 :1 :1
## M1:1 R:0 I:0
## U :0 S:0 R:0
## X:0 S:0
## X:0
##
##
## Ampicillin Bactracin Carbenicillin Ceftazidime Ceftiofur Cefuroxime Cephalexin
## :0 :1 :1 :1 :0 :1 :1
## R:0 S:0 R:0 R:0 R:0 S:0 R:0
## S:1 S:0 S:0 S:1 S:0
## X:0 X:0 X:0 X:0 X:0
##
##
##
## Chloramphenicol Ciprofloxacin Clavulox Clavulox_Clavulox Clindamycin
## :1 :1 :1 :1 :1
## R:0 I:0 R:0 S:0 R:0
## S:0 R:0 S:0 S:0
## X:0 S:0 X:0 X:0
##
##
##
## Cloxacillin Doxcycline Doxycycline Doxyycline Enrofloxacin Enrofloxaxin
## :1 :1 :1 :1 :0 :1
## R:0 R:0 I:0 S:0 I:0 R:0
## S:0 S:0 R:0 R:0
## X:0 S:0 S:1
## X:0
##
##
## Erythromycin Fusidic_Acid Gentamicin Lincomycin Marbofloxacin Minocycline
## :1 :1 :0 :1 :1 :1
## R:0 R:0 I:0 R:0 I:0 S:0
## S:0 S:0 R:0 S:0 R:0
## X:0 X:0 S:1 S:0
## X:0
##
##
## Mupirocin Neomycin Norfloxacin Novobiocin Ofloxacin Penicillin Penicillin_G
## :1 :0 :1 :1 :1 :1 :0
## R:0 I:0 R:0 R:0 R:0 R:0 R:0
## S:0 R:0 S:0 S:0 S:0 S:0 S:1
## S:1 X:0 X:0
## X:0
##
##
## Pipercillin Polymyxin_B Rifampicin Soframycin Streptomycin
## :1 :1 :1 :1 Mode:logical
## S:0 R:0 R:0 R:0 NA's:1
## S:0 S:0 S:0
## X:0 X:0 X:0
##
##
##
## Sulphatrimethoprim Tetracycline Ticarcillin Timentin TestDate
## :0 :0 :1 :0 Mode:logical
## R:0 I:0 R:0 I:0 NA's:1
## S:1 R:0 S:0 R:0
## X:0 S:1 X:0 S:1
## X:0 X:0
##
##
## ULeucocytes UEpithelial URBC UCasts
## :1 :1 :1 :1
## <1 :0 <1 :0 <1 :0 None seen:0
## >200 :0 1 :0 >200:0
## 135 :0 18 :0 1 :0
## 2 :0 2 :0 116 :0
## 29 :0 36 :0 3 :0
## (Other):0 (Other):0
## UCrystals UBacteria USpermatoza
## :1 :1 Mode:logical
## 1+ Ca carbonate :0 1+ :0 NA's:1
## 3+ Ca carbonate, Occ Ca oxalate :0 3+ :0
## 3+ calcium carbonate :0 Few :0
## 3+ Calcium carbonate, few calcium oxalate:0 Occasional:0
## Ca carbonate 3+ :0
## (Other) :0
## UFungal UDebris UGram USG UPh UProtein
## :1 :1 Mode:logical Min. : NA :1 :1
## 2+ Hyphae:0 1+:0 NA's:1 1st Qu.: NA >=9.0:0 1+ :0
## 3+:0 Median : NA 6 :0 2+ :0
## Mean :NaN 8 :0 3+ :0
## 3rd Qu.: NA 8.5 :0 Negative:0
## Max. : NA 9 :0
## NA's :1
## UGlucose UKetones UBlood UBilirubin UCultureNote
## :1 :1 :1 :1 :1
## 3+ :0 1+ :0 1+ :0 1+ :0 >10,000 CFU/mL :0
## Negative:0 Negative:0 3+ :0 3+ :0 >100,000 CFU/mL:0
## Trace :0 Trace :0 Negative:0 Negative:0 2,000 CFU/mL :0
## 3,200 CFU/mL :0
## 4,000 CFU/mL :0
## (Other) :0
## M1Leucocytes M1RBC M1Epithelial
## :1 :1 :1
## 1+ :0 1+ :0 1+ :0
## 2+ :0 2+ :0 2+ :0
## 3+ :0 3+ :0 3+ :0
## FEW :0 FEW :0 FEW :0
## NONE SEEN:0 NONE SEEN:0 NONE SEEN:0
## (Other) :0 (Other) :0 (Other) :0
## M1Fungal FMicroForExcess FLeucocytes
## :1 Mode:logical :1
## 2+ fungal hyphae :0 NA's:1 None seen:0
## 2+ Yeast cells and Fungal Hyphae:0
## FEW * :0
## FEW FUNGAL HYPHAE :0
## FUNGAL HYPHAE 1+ :0
## (Other) :0
## FRBC FCampylobacter FYeast FProtozoaCystsOva
## :1 :1 :1 :1
## None seen:0 None seen:0 None seen :0 None seen:0
## Very occasional:0
##
##
##
##
## FTrypsin10 FTrypsin100 FOccultBlood
## Mode:logical Mode:logical Mode:logical
## NA's:1 NA's:1 NA's:1
##
##
##
##
##

comamonas <- repro_only[grepl("Comamonas|comamonas",repro_only$Culture),]
comamonas$Culture <- as.factor(as.character(comamonas$Culture))
comamonas$Specimens <- as.factor(as.character(comamonas$Specimens))
summary(comamonas$Culture)

## Comamonas testosteroni A*
## 1

summary(comamonas$Specimens)

## Uterine swab-J, Uterine biopsy H x 1
## 1

summary(comamonas)

## ï..Job_Number ClientCode JobDate Species PostCode
## Min. :620567 Min. :692 16/4/2018:1 EQU:1 Min. :6214
## 1st Qu.:620567 1st Qu.:692 1/1/2016 :0 1st Qu.:6214
## Median :620567 Median :692 1/10/2015:0 Median :6214
## Mean :620567 Mean :692 1/10/2019:0 Mean :6214
## 3rd Qu.:620567 3rd Qu.:692 1/11/2016:0 3rd Qu.:6214
## Max. :620567 Max. :692 1/11/2017:0 Max. :6214
## (Other) :0
## Specimens Prefix Culture
## Uterine swab-J, Uterine biopsy H x 1:1 F :0 Comamonas testosteroni A*:1
## M1:1
## U :0
##
##
##
##
## Amikacin Amoxycillin Ampicillin Bactracin Carbenicillin Ceftazidime Ceftiofur
## :1 :1 :0 :1 :1 :1 :0
## R:0 I:0 R:0 S:0 R:0 R:0 R:0
## S:0 R:0 S:1 S:0 S:0 S:1
## X:0 S:0 X:0 X:0 X:0 X:0
## X:0
##
##
## Cefuroxime Cephalexin Chloramphenicol Ciprofloxacin Clavulox Clavulox_Clavulox
## :1 :1 :1 :1 :1 :1
## S:0 R:0 R:0 I:0 R:0 S:0
## S:0 S:0 R:0 S:0
## X:0 X:0 S:0 X:0
##
##
##
## Clindamycin Cloxacillin Doxcycline Doxycycline Doxyycline Enrofloxacin
## :1 :1 :1 :1 :1 :0
## R:0 R:0 R:0 I:0 S:0 I:0
## S:0 S:0 S:0 R:0 R:0
## X:0 X:0 S:0 S:1
## X:0
##
##
## Enrofloxaxin Erythromycin Fusidic_Acid Gentamicin Lincomycin Marbofloxacin
## :1 :1 :1 :0 :1 :1
## R:0 R:0 R:0 I:0 R:0 I:0
## S:0 S:0 R:1 S:0 R:0
## X:0 X:0 S:0 S:0
## X:0
##
##
## Minocycline Mupirocin Neomycin Norfloxacin Novobiocin Ofloxacin Penicillin
## :1 :1 :0 :1 :1 :1 :1
## S:0 R:0 I:0 R:0 R:0 R:0 R:0
## S:0 R:1 S:0 S:0 S:0 S:0
## S:0 X:0
## X:0
##
##
## Penicillin_G Pipercillin Polymyxin_B Rifampicin Soframycin Streptomycin
## :0 :1 :1 :1 :1 Mode:logical
## R:1 S:0 R:0 R:0 R:0 NA's:1
## S:0 S:0 S:0 S:0
## X:0 X:0 X:0 X:0
##
##
##
## Sulphatrimethoprim Tetracycline Ticarcillin Timentin TestDate
## :0 :0 :1 :1 Mode:logical
## R:0 I:0 R:0 I:0 NA's:1
## S:1 R:0 S:0 R:0
## X:0 S:1 X:0 S:0
## X:0 X:0
##
##
## ULeucocytes UEpithelial URBC UCasts
## :1 :1 :1 :1
## <1 :0 <1 :0 <1 :0 None seen:0
## >200 :0 1 :0 >200:0
## 135 :0 18 :0 1 :0
## 2 :0 2 :0 116 :0
## 29 :0 36 :0 3 :0
## (Other):0 (Other):0
## UCrystals UBacteria USpermatoza
## :1 :1 Mode:logical
## 1+ Ca carbonate :0 1+ :0 NA's:1
## 3+ Ca carbonate, Occ Ca oxalate :0 3+ :0
## 3+ calcium carbonate :0 Few :0
## 3+ Calcium carbonate, few calcium oxalate:0 Occasional:0
## Ca carbonate 3+ :0
## (Other) :0
## UFungal UDebris UGram USG UPh UProtein
## :1 :1 Mode:logical Min. : NA :1 :1
## 2+ Hyphae:0 1+:0 NA's:1 1st Qu.: NA >=9.0:0 1+ :0
## 3+:0 Median : NA 6 :0 2+ :0
## Mean :NaN 8 :0 3+ :0
## 3rd Qu.: NA 8.5 :0 Negative:0
## Max. : NA 9 :0
## NA's :1
## UGlucose UKetones UBlood UBilirubin UCultureNote
## :1 :1 :1 :1 :1
## 3+ :0 1+ :0 1+ :0 1+ :0 >10,000 CFU/mL :0
## Negative:0 Negative:0 3+ :0 3+ :0 >100,000 CFU/mL:0
## Trace :0 Trace :0 Negative:0 Negative:0 2,000 CFU/mL :0
## 3,200 CFU/mL :0
## 4,000 CFU/mL :0
## (Other) :0
## M1Leucocytes M1RBC M1Epithelial
## :1 :1 :1
## 1+ :0 1+ :0 1+ :0
## 2+ :0 2+ :0 2+ :0
## 3+ :0 3+ :0 3+ :0
## FEW :0 FEW :0 FEW :0
## NONE SEEN:0 NONE SEEN:0 NONE SEEN:0
## (Other) :0 (Other) :0 (Other) :0
## M1Fungal FMicroForExcess FLeucocytes
## :1 Mode:logical :1
## 2+ fungal hyphae :0 NA's:1 None seen:0
## 2+ Yeast cells and Fungal Hyphae:0
## FEW * :0
## FEW FUNGAL HYPHAE :0
## FUNGAL HYPHAE 1+ :0
## (Other) :0
## FRBC FCampylobacter FYeast FProtozoaCystsOva
## :1 :1 :1 :1
## None seen:0 None seen:0 None seen :0 None seen:0
## Very occasional:0
##
##
##
##
## FTrypsin10 FTrypsin100 FOccultBlood
## Mode:logical Mode:logical Mode:logical
## NA's:1 NA's:1 NA's:1
##
##
##
##
##

pantoea <- repro_only[grepl("Pantoea|pantoea",repro_only$Culture),]
pantoea$Culture <- as.factor(as.character(pantoea$Culture))
pantoea$Specimens <- as.factor(as.character(pantoea$Specimens))
summary(pantoea$Culture)

## Pantoea sp. Pantoea sp.*
## 6 1

summary(pantoea$Specimens)

## uterine lavage H x 1 Uterine Lavage H x 1
## 1 1
## Uterine swab-J Uterine swab-J, Uterine swab- L
## 1 1
## Uterine swab-L Uterus lesion swab-J
## 1 1
## Vaginal swab-J
## 1

summary(pantoea)

## ï..Job_Number ClientCode JobDate Species PostCode
## Min. :408759 Min. : 101.0 1/10/2019 :1 EQU:7 Min. :6125
## 1st Qu.:577565 1st Qu.: 101.0 12/9/2018 :1 1st Qu.:6163
## Median :749623 Median :1131.0 15/11/2016:1 Median :6163
## Mean :654751 Mean : 736.3 25/9/2015 :1 Mean :6161
## 3rd Qu.:753461 3rd Qu.:1240.0 26/11/2019:1 3rd Qu.:6171
## Max. :762825 Max. :1240.0 29/10/2019:1 Max. :6171
## (Other) :1
## Specimens Prefix Culture Amikacin
## uterine lavage H x 1 :1 F :0 Pantoea sp. :6 :7
## Uterine Lavage H x 1 :1 M1:7 Pantoea sp.*:1 R:0
## Uterine swab-J :1 U :0 S:0
## Uterine swab-J, Uterine swab- L:1 X:0
## Uterine swab-L :1
## Uterus lesion swab-J :1
## Vaginal swab-J :1
## Amoxycillin Ampicillin Bactracin Carbenicillin Ceftazidime Ceftiofur
## :7 :0 :7 :7 :7 :0
## I:0 R:4 S:0 R:0 R:0 R:1
## R:0 S:3 S:0 S:0 S:6
## S:0 X:0 X:0 X:0 X:0
## X:0
##
##
## Cefuroxime Cephalexin Chloramphenicol Ciprofloxacin Clavulox Clavulox_Clavulox
## :7 :7 :7 :7 :7 :7
## S:0 R:0 R:0 I:0 R:0 S:0
## S:0 S:0 R:0 S:0
## X:0 X:0 S:0 X:0
##
##
##
## Clindamycin Cloxacillin Doxcycline Doxycycline Doxyycline Enrofloxacin
## :7 :7 :7 :7 :7 :0
## R:0 R:0 R:0 I:0 S:0 I:0
## S:0 S:0 S:0 R:0 R:0
## X:0 X:0 S:0 S:7
## X:0
##
##
## Enrofloxaxin Erythromycin Fusidic_Acid Gentamicin Lincomycin Marbofloxacin
## :7 :7 :7 :0 :7 :7
## R:0 R:0 R:0 I:0 R:0 I:0
## S:0 S:0 R:1 S:0 R:0
## X:0 X:0 S:6 S:0
## X:0
##
##
## Minocycline Mupirocin Neomycin Norfloxacin Novobiocin Ofloxacin Penicillin
## :7 :7 :0 :7 :7 :7 :7
## S:0 R:0 I:0 R:0 R:0 R:0 R:0
## S:0 R:1 S:0 S:0 S:0 S:0
## S:6 X:0
## X:0
##
##
## Penicillin_G Pipercillin Polymyxin_B Rifampicin Soframycin Streptomycin
## :0 :7 :7 :7 :7 Mode:logical
## R:6 S:0 R:0 R:0 R:0 NA's:7
## S:1 S:0 S:0 S:0
## X:0 X:0 X:0 X:0
##
##
##
## Sulphatrimethoprim Tetracycline Ticarcillin Timentin TestDate
## :0 :0 :7 :5 Mode:logical
## R:2 I:0 R:0 I:0 NA's:7
## S:5 R:1 S:0 R:0
## X:0 S:6 X:0 S:2
## X:0 X:0
##
##
## ULeucocytes UEpithelial URBC UCasts
## :7 :7 :7 :7
## <1 :0 <1 :0 <1 :0 None seen:0
## >200 :0 1 :0 >200:0
## 135 :0 18 :0 1 :0
## 2 :0 2 :0 116 :0
## 29 :0 36 :0 3 :0
## (Other):0 (Other):0
## UCrystals UBacteria USpermatoza
## :7 :7 Mode:logical
## 1+ Ca carbonate :0 1+ :0 NA's:7
## 3+ Ca carbonate, Occ Ca oxalate :0 3+ :0
## 3+ calcium carbonate :0 Few :0
## 3+ Calcium carbonate, few calcium oxalate:0 Occasional:0
## Ca carbonate 3+ :0
## (Other) :0
## UFungal UDebris UGram USG UPh UProtein
## :7 :7 Mode:logical Min. : NA :7 :7
## 2+ Hyphae:0 1+:0 NA's:7 1st Qu.: NA >=9.0:0 1+ :0
## 3+:0 Median : NA 6 :0 2+ :0
## Mean :NaN 8 :0 3+ :0
## 3rd Qu.: NA 8.5 :0 Negative:0
## Max. : NA 9 :0
## NA's :7
## UGlucose UKetones UBlood UBilirubin UCultureNote
## :7 :7 :7 :7 :7
## 3+ :0 1+ :0 1+ :0 1+ :0 >10,000 CFU/mL :0
## Negative:0 Negative:0 3+ :0 3+ :0 >100,000 CFU/mL:0
## Trace :0 Trace :0 Negative:0 Negative:0 2,000 CFU/mL :0
## 3,200 CFU/mL :0
## 4,000 CFU/mL :0
## (Other) :0
## M1Leucocytes M1RBC M1Epithelial
## :7 :7 :7
## 1+ :0 1+ :0 1+ :0
## 2+ :0 2+ :0 2+ :0
## 3+ :0 3+ :0 3+ :0
## FEW :0 FEW :0 FEW :0
## NONE SEEN:0 NONE SEEN:0 NONE SEEN:0
## (Other) :0 (Other) :0 (Other) :0
## M1Fungal FMicroForExcess FLeucocytes
## :7 Mode:logical :7
## 2+ fungal hyphae :0 NA's:7 None seen:0
## 2+ Yeast cells and Fungal Hyphae:0
## FEW * :0
## FEW FUNGAL HYPHAE :0
## FUNGAL HYPHAE 1+ :0
## (Other) :0
## FRBC FCampylobacter FYeast FProtozoaCystsOva
## :7 :7 :7 :7
## None seen:0 None seen:0 None seen :0 None seen:0
## Very occasional:0
##
##
##
##
## FTrypsin10 FTrypsin100 FOccultBlood
## Mode:logical Mode:logical Mode:logical
## NA's:7 NA's:7 NA's:7
##
##
##
##
##

nocardia <- repro_only[grepl("Nocardia|nocardia",repro_only$Culture),]
nocardia$Culture <- as.factor(as.character(nocardia$Culture))
nocardia$Specimens <- as.factor(as.character(nocardia$Specimens))
summary(nocardia$Culture)

## Probable Nocardia sp.
## 1

summary(nocardia$Specimens)

## Uterine swab-J
## 1

summary(nocardia)

## ï..Job_Number ClientCode JobDate Species PostCode
## Min. :512932 Min. :101 31/12/2016:1 EQU:1 Min. :6171
## 1st Qu.:512932 1st Qu.:101 1/1/2016 :0 1st Qu.:6171
## Median :512932 Median :101 1/10/2015 :0 Median :6171
## Mean :512932 Mean :101 1/10/2019 :0 Mean :6171
## 3rd Qu.:512932 3rd Qu.:101 1/11/2016 :0 3rd Qu.:6171
## Max. :512932 Max. :101 1/11/2017 :0 Max. :6171
## (Other) :0
## Specimens Prefix Culture Amikacin Amoxycillin
## Uterine swab-J:1 F :0 Probable Nocardia sp.:1 :1 :1
## M1:1 R:0 I:0
## U :0 S:0 R:0
## X:0 S:0
## X:0
##
##
## Ampicillin Bactracin Carbenicillin Ceftazidime Ceftiofur Cefuroxime Cephalexin
## :0 :1 :1 :1 :0 :1 :1
## R:1 S:0 R:0 R:0 R:1 S:0 R:0
## S:0 S:0 S:0 S:0 S:0
## X:0 X:0 X:0 X:0 X:0
##
##
##
## Chloramphenicol Ciprofloxacin Clavulox Clavulox_Clavulox Clindamycin
## :1 :1 :1 :1 :1
## R:0 I:0 R:0 S:0 R:0
## S:0 R:0 S:0 S:0
## X:0 S:0 X:0 X:0
##
##
##
## Cloxacillin Doxcycline Doxycycline Doxyycline Enrofloxacin Enrofloxaxin
## :1 :1 :1 :1 :0 :1
## R:0 R:0 I:0 S:0 I:0 R:0
## S:0 S:0 R:0 R:1
## X:0 S:0 S:0
## X:0
##
##
## Erythromycin Fusidic_Acid Gentamicin Lincomycin Marbofloxacin Minocycline
## :1 :1 :0 :1 :1 :1
## R:0 R:0 I:0 R:0 I:0 S:0
## S:0 S:0 R:0 S:0 R:0
## X:0 X:0 S:1 S:0
## X:0
##
##
## Mupirocin Neomycin Norfloxacin Novobiocin Ofloxacin Penicillin Penicillin_G
## :1 :0 :1 :1 :1 :1 :0
## R:0 I:0 R:0 R:0 R:0 R:0 R:1
## S:0 R:0 S:0 S:0 S:0 S:0 S:0
## S:1 X:0 X:0
## X:0
##
##
## Pipercillin Polymyxin_B Rifampicin Soframycin Streptomycin
## :1 :1 :1 :1 Mode:logical
## S:0 R:0 R:0 R:0 NA's:1
## S:0 S:0 S:0
## X:0 X:0 X:0
##
##
##
## Sulphatrimethoprim Tetracycline Ticarcillin Timentin TestDate
## :0 :0 :1 :1 Mode:logical
## R:1 I:0 R:0 I:0 NA's:1
## S:0 R:1 S:0 R:0
## X:0 S:0 X:0 S:0
## X:0 X:0
##
##
## ULeucocytes UEpithelial URBC UCasts
## :1 :1 :1 :1
## <1 :0 <1 :0 <1 :0 None seen:0
## >200 :0 1 :0 >200:0
## 135 :0 18 :0 1 :0
## 2 :0 2 :0 116 :0
## 29 :0 36 :0 3 :0
## (Other):0 (Other):0
## UCrystals UBacteria USpermatoza
## :1 :1 Mode:logical
## 1+ Ca carbonate :0 1+ :0 NA's:1
## 3+ Ca carbonate, Occ Ca oxalate :0 3+ :0
## 3+ calcium carbonate :0 Few :0
## 3+ Calcium carbonate, few calcium oxalate:0 Occasional:0
## Ca carbonate 3+ :0
## (Other) :0
## UFungal UDebris UGram USG UPh UProtein
## :1 :1 Mode:logical Min. : NA :1 :1
## 2+ Hyphae:0 1+:0 NA's:1 1st Qu.: NA >=9.0:0 1+ :0
## 3+:0 Median : NA 6 :0 2+ :0
## Mean :NaN 8 :0 3+ :0
## 3rd Qu.: NA 8.5 :0 Negative:0
## Max. : NA 9 :0
## NA's :1
## UGlucose UKetones UBlood UBilirubin UCultureNote
## :1 :1 :1 :1 :1
## 3+ :0 1+ :0 1+ :0 1+ :0 >10,000 CFU/mL :0
## Negative:0 Negative:0 3+ :0 3+ :0 >100,000 CFU/mL:0
## Trace :0 Trace :0 Negative:0 Negative:0 2,000 CFU/mL :0
## 3,200 CFU/mL :0
## 4,000 CFU/mL :0
## (Other) :0
## M1Leucocytes M1RBC M1Epithelial
## :1 :1 :1
## 1+ :0 1+ :0 1+ :0
## 2+ :0 2+ :0 2+ :0
## 3+ :0 3+ :0 3+ :0
## FEW :0 FEW :0 FEW :0
## NONE SEEN:0 NONE SEEN:0 NONE SEEN:0
## (Other) :0 (Other) :0 (Other) :0
## M1Fungal FMicroForExcess FLeucocytes
## :1 Mode:logical :1
## 2+ fungal hyphae :0 NA's:1 None seen:0
## 2+ Yeast cells and Fungal Hyphae:0
## FEW * :0
## FEW FUNGAL HYPHAE :0
## FUNGAL HYPHAE 1+ :0
## (Other) :0
## FRBC FCampylobacter FYeast FProtozoaCystsOva
## :1 :1 :1 :1
## None seen:0 None seen:0 None seen :0 None seen:0
## Very occasional:0
##
##
##
##
## FTrypsin10 FTrypsin100 FOccultBlood
## Mode:logical Mode:logical Mode:logical
## NA's:1 NA's:1 NA's:1
##
##
##
##
##

contaminant <- repro_only[grepl("Contaminant|contaminant",repro_only$Culture),]
contaminant$Culture <- as.factor(as.character(contaminant$Culture))
contaminant$Specimens <- as.factor(as.character(contaminant$Specimens))
summary(contaminant$Culture)

## Environmental contaminant
## 1

summary(contaminant$Specimens)

## Uterine swab-L
## 1

summary(contaminant)

## ï..Job_Number ClientCode JobDate Species PostCode
## Min. :619219 Min. :72 10/4/2018:1 EQU:1 Min. :6231
## 1st Qu.:619219 1st Qu.:72 1/1/2016 :0 1st Qu.:6231
## Median :619219 Median :72 1/10/2015:0 Median :6231
## Mean :619219 Mean :72 1/10/2019:0 Mean :6231
## 3rd Qu.:619219 3rd Qu.:72 1/11/2016:0 3rd Qu.:6231
## Max. :619219 Max. :72 1/11/2017:0 Max. :6231
## (Other) :0
## Specimens Prefix Culture Amikacin Amoxycillin
## Uterine swab-L:1 F :0 Environmental contaminant:1 :1 :1
## M1:1 R:0 I:0
## U :0 S:0 R:0
## X:0 S:0
## X:0
##
##
## Ampicillin Bactracin Carbenicillin Ceftazidime Ceftiofur Cefuroxime Cephalexin
## :0 :1 :1 :1 :0 :1 :1
## R:1 S:0 R:0 R:0 R:1 S:0 R:0
## S:0 S:0 S:0 S:0 S:0
## X:0 X:0 X:0 X:0 X:0
##
##
##
## Chloramphenicol Ciprofloxacin Clavulox Clavulox_Clavulox Clindamycin
## :1 :1 :1 :1 :1
## R:0 I:0 R:0 S:0 R:0
## S:0 R:0 S:0 S:0
## X:0 S:0 X:0 X:0
##
##
##
## Cloxacillin Doxcycline Doxycycline Doxyycline Enrofloxacin Enrofloxaxin
## :1 :1 :1 :1 :0 :1
## R:0 R:0 I:0 S:0 I:0 R:0
## S:0 S:0 R:0 R:0
## X:0 S:0 S:1
## X:0
##
##
## Erythromycin Fusidic_Acid Gentamicin Lincomycin Marbofloxacin Minocycline
## :1 :1 :0 :1 :1 :1
## R:0 R:0 I:0 R:0 I:0 S:0
## S:0 S:0 R:0 S:0 R:0
## X:0 X:0 S:1 S:0
## X:0
##
##
## Mupirocin Neomycin Norfloxacin Novobiocin Ofloxacin Penicillin Penicillin_G
## :1 :0 :1 :1 :1 :1 :0
## R:0 I:0 R:0 R:0 R:0 R:0 R:1
## S:0 R:0 S:0 S:0 S:0 S:0 S:0
## S:1 X:0 X:0
## X:0
##
##
## Pipercillin Polymyxin_B Rifampicin Soframycin Streptomycin
## :1 :1 :1 :1 Mode:logical
## S:0 R:0 R:0 R:0 NA's:1
## S:0 S:0 S:0
## X:0 X:0 X:0
##
##
##
## Sulphatrimethoprim Tetracycline Ticarcillin Timentin TestDate
## :0 :0 :1 :1 Mode:logical
## R:0 I:0 R:0 I:0 NA's:1
## S:1 R:0 S:0 R:0
## X:0 S:1 X:0 S:0
## X:0 X:0
##
##
## ULeucocytes UEpithelial URBC UCasts
## :1 :1 :1 :1
## <1 :0 <1 :0 <1 :0 None seen:0
## >200 :0 1 :0 >200:0
## 135 :0 18 :0 1 :0
## 2 :0 2 :0 116 :0
## 29 :0 36 :0 3 :0
## (Other):0 (Other):0
## UCrystals UBacteria USpermatoza
## :1 :1 Mode:logical
## 1+ Ca carbonate :0 1+ :0 NA's:1
## 3+ Ca carbonate, Occ Ca oxalate :0 3+ :0
## 3+ calcium carbonate :0 Few :0
## 3+ Calcium carbonate, few calcium oxalate:0 Occasional:0
## Ca carbonate 3+ :0
## (Other) :0
## UFungal UDebris UGram USG UPh UProtein
## :1 :1 Mode:logical Min. : NA :1 :1
## 2+ Hyphae:0 1+:0 NA's:1 1st Qu.: NA >=9.0:0 1+ :0
## 3+:0 Median : NA 6 :0 2+ :0
## Mean :NaN 8 :0 3+ :0
## 3rd Qu.: NA 8.5 :0 Negative:0
## Max. : NA 9 :0
## NA's :1
## UGlucose UKetones UBlood UBilirubin UCultureNote
## :1 :1 :1 :1 :1
## 3+ :0 1+ :0 1+ :0 1+ :0 >10,000 CFU/mL :0
## Negative:0 Negative:0 3+ :0 3+ :0 >100,000 CFU/mL:0
## Trace :0 Trace :0 Negative:0 Negative:0 2,000 CFU/mL :0
## 3,200 CFU/mL :0
## 4,000 CFU/mL :0
## (Other) :0
## M1Leucocytes M1RBC M1Epithelial
## :1 :1 :1
## 1+ :0 1+ :0 1+ :0
## 2+ :0 2+ :0 2+ :0
## 3+ :0 3+ :0 3+ :0
## FEW :0 FEW :0 FEW :0
## NONE SEEN:0 NONE SEEN:0 NONE SEEN:0
## (Other) :0 (Other) :0 (Other) :0
## M1Fungal FMicroForExcess FLeucocytes
## :1 Mode:logical :1
## 2+ fungal hyphae :0 NA's:1 None seen:0
## 2+ Yeast cells and Fungal Hyphae:0
## FEW * :0
## FEW FUNGAL HYPHAE :0
## FUNGAL HYPHAE 1+ :0
## (Other) :0
## FRBC FCampylobacter FYeast FProtozoaCystsOva
## :1 :1 :1 :1
## None seen:0 None seen:0 None seen :0 None seen:0
## Very occasional:0
##
##
##
##
## FTrypsin10 FTrypsin100 FOccultBlood
## Mode:logical Mode:logical Mode:logical
## NA's:1 NA's:1 NA's:1
##
##
##
##
##

#CEFTIOFUR
#Have a look the job dates
repro_only$JobDate <- as.factor(repro_only$JobDate)
summary(repro_only$JobDate)

## 7/9/2018 20/12/2016 18/10/2016 1/9/2018 21/9/2018 15/11/2018 30/8/2016
## 14 12 9 8 8 7 6
## 6/9/2019 8/11/2018 1/11/2018 18/12/2018 20/11/2018 22/10/2015 26/10/2016
## 6 6 5 5 5 5 5
## 27/10/2018 28/10/2015 28/8/2019 29/9/2018 3/11/2015 30/11/2017 5/11/2018
## 5 5 5 5 5 5 5
## 6/11/2019 8/11/2019 9/12/2019 10/10/2018 12/3/2020 13/11/2017 15/11/2016
## 5 5 5 4 4 4 4
## 15/9/2017 17/11/2015 2/10/2019 20/10/2015 20/11/2015 21/11/2019 23/11/2017
## 4 4 4 4 4 4 4
## 23/12/2019 25/10/2018 25/9/2015 26/11/2019 28/12/2018 28/9/2019 31/8/2018
## 4 4 4 4 4 4 4
## 4/10/2019 4/11/2016 4/11/2017 5/11/2015 7/11/2019 7/9/2016 11/10/2018
## 4 4 4 4 4 4 3
## 11/11/2015 11/9/2018 12/12/2015 12/2/2018 12/9/2017 13/1/2020 13/10/2015
## 3 3 3 3 3 3 3
## 13/11/2019 13/12/2016 14/11/2018 16/10/2015 16/11/2017 16/12/2015 17/10/2017
## 3 3 3 3 3 3 3
## 17/9/2019 18/11/2016 19/11/2016 2/1/2019 2/11/2016 2/2/2017 2/9/2015
## 3 3 3 3 3 3 3
## 2/9/2019 21/10/2019 21/11/2015 22/10/2018 23/11/2015 23/8/2018 25/10/2019
## 3 3 3 3 3 3 3
## 25/11/2016 25/8/2017 26/11/2018 27/2/2019 28/11/2019 28/9/2017 29/11/2017
## 3 3 3 3 3 3 3
## 29/11/2019 30/10/2019 30/11/2018 30/8/2018 31/1/2020 31/8/2019 4/12/2019
## 3 3 3 3 3 3 3
## 5/10/2016 5/3/2019 6/10/2015 6/10/2018 6/11/2015 6/12/2016 7/2/2019
## 3 3 3 3 3 3 3
## 8/10/2016 (Other)
## 3 484

#Remove contaminant from the culture list
repro_only$Culture <- as.factor(as.character(repro_only$Culture))
repro_only <- repro_only[grepl("contaminant",repro_only$Culture)==F,]
summary(repro_only$Culture)

## Non haem E. coli Beta haem. Streptococcus
## 253 171
## Enterococcus sp. Beta haem. Streptococcus#
## 48 37
## Pseudomonas aeruginosa Serratia marcescens
## 28 24
## Enterobacter sp. Klebsiella pneumoniae
## 23 18
## Staphylococcus aureus Haem E.coli
## 12 11
## Enterobacter sp.* Staphylococcus pseudintermedius
## 9 8
## Non haem E. coli* Staphylococcus capitis
## 7 7
## UNIDENTIFIED Acinetobacter lwoffii
## 7 6
## Corynebacterium sp. Pantoea sp.
## 6 6
## Streptococcus uberis Enterobacter aerogenes
## 6 5
## Klebsiella oxytoca Klebsiella pneumoniae*
## 5 5
## Sphingomonas paucimobilis Staphylococcus warneri
## 5 5
## Citrobacter koseri Enterococcus sp.*
## 4 4
## Kocuria rosea Proteus mirabilis
## 4 4
## Staphylococcus schleiferi Acinetobacter lwoffii*
## 4 3
## Enterobacter sp.# Non haem E. coli#
## 3 3
## Staphylococcus haemolyticus* Staphylococcus xylosus
## 3 3
## Streptococcus thoraltensis Acinetobacter sp.
## 3 2
## Actinomyces species Arthrobacter spp.
## 2 2
## Citrobacter koseri* Enterobacter aerogenes*
## 2 2
## Kocuria kristinae* Lactococcus garvieae
## 2 2
## Non haem Streptococcus* Pseudomonas sp.
## 2 2
## Pseudomonas sp.* Pseudomonas stutzeri*
## 2 2
## Serratia liquefaciens Staphylococcus sciuri*
## 2 2
## Staphylococcus xylosus* Streptococcus alactolyticus*
## 2 2
## Streptococcus pseudoporcinus Streptococcus sanguinis
## 2 2
## Achromobacter denitrificans* Achromobacter sp.*
## 1 1
## Achromobacter xylosoxidans Acinetobacter haemolyticus
## 1 1
## Acinetobacter iwoffii Acinetobacter lwoffii#
## 1 1
## Acinetobacter radioresistens Acinetobacter sp.*
## 1 1
## Actinobacillus sp. Actinobacillus suis/equuli
## 1 1
## Aerococcus viridans* Aeromonas media
## 1 1
## Aeromonas sobria Aeromonas sp.*
## 1 1
## Arcanobacterium haemolyticum Arthrobacter spp
## 1 1
## Beta haem. Streptococcus # Beta haem. Streptococcus*
## 1 1
## Bordetella bronchiseptica Bordetella bronchiseptica*
## 1 1
## Bordetella sp. Brevibacterium spp. ##
## 1 1
## Candida parasilosis# Cellulomonas spp/Microbacterium spp
## 1 1
## Cellulomonas spp/Microbacterium spp. Cellulomonas spp/Microbacterium spp.*
## 1 1
## Citrobacter amalonaticus Citrobacter freundii
## 1 1
## Citrobacter freundii# Citrobacter sp.
## 1 1
## Coagulase negative Staphylococcus sp. Comamonas testosteroni A*
## 1 1
## Corynebacterium glucuronolyticum Corynebacterium propinquum
## 1 1
## Corynebacterium sp.* Corynebacterium urealyticum
## 1 1
## Enterococcus casseliflavus Enterococcus faecalis
## 1 1
## Enterococcus sp. (beta haemolytic) Escherichia hermannii
## 1 1
## Escherichia hermannii* Escherichia vulneris
## 1 1
## Ewingella americana Klebsiella oxytoca*
## 1 1
## Kluyvera intermedia* Kocuria kristinae
## 1 1
## Kocuria rosea* (Other)
## 1 52

#Make groups for all of the data from 2015, 2016, 2017, 2018, 2019, 2020
Year2015 <- repro_only[grepl("2015",repro_only$JobDate),]
Year2015$JobDate <- as.factor(as.character(Year2015$JobDate))
Year2015$Specimens <- as.factor(as.character(Year2015$Specimens))
summary(Year2015$JobDate)

## 1/10/2015 10/10/2015 10/11/2015 10/12/2015 11/11/2015 11/12/2015 12/11/2015
## 2 2 1 1 3 1 1
## 12/12/2015 12/8/2015 12/9/2015 13/10/2015 13/11/2015 14/10/2015 14/9/2015
## 3 1 1 3 2 2 1
## 15/12/2015 15/9/2015 16/10/2015 16/11/2015 16/12/2015 17/11/2015 17/12/2015
## 2 1 3 2 3 4 1
## 17/9/2015 18/12/2015 18/8/2015 18/9/2015 19/8/2015 19/9/2015 2/10/2015
## 1 1 1 2 1 2 1
## 2/11/2015 2/12/2015 2/9/2015 20/10/2015 20/11/2015 21/11/2015 22/10/2015
## 1 1 3 4 4 3 5
## 22/8/2015 22/9/2015 23/10/2015 23/11/2015 23/12/2015 23/9/2015 24/10/2015
## 1 1 2 3 2 1 2
## 24/11/2015 24/9/2015 25/11/2015 25/12/2015 25/9/2015 26/10/2015 27/10/2015
## 1 2 2 1 4 1 2
## 27/11/2015 28/10/2015 29/10/2015 29/8/2015 3/10/2015 3/11/2015 3/12/2015
## 2 5 2 1 2 5 2
## 3/8/2015 30/10/2015 30/11/2015 30/12/2015 30/9/2015 31/10/2015 31/8/2015
## 1 2 1 2 2 1 1
## 4/11/2015 4/12/2015 5/10/2015 5/11/2015 6/10/2015 6/11/2015 7/10/2015
## 2 1 1 4 3 3 1
## 7/11/2015 7/12/2015 7/8/2015 7/9/2015 9/10/2015 9/11/2015 9/12/2015
## 1 1 1 1 2 2 1
## 9/9/2015
## 1

summary(Year2015$Specimens)

## 1 x Uterine Container
## 1
## Cervical swab-J
## 4
## Clitoral Swab - J
## 1
## Placenta Swab Jx 1
## 1
## Swab mare x1 J
## 1
## Uterine avage fluid H x 1
## 1
## Uterine Fluid BH
## 1
## Uterine Fluid D x 2
## 1
## Uterine Fluid H x 1
## 4
## Uterine Fluid H x 1 , add 16/11
## 1
## Uterine Flush Hx 1
## 1
## Uterine Lavage
## 1
## Uterine Lavage 2 x 50ml Tubes
## 1
## Uterine Lavage H x 1
## 2
## Uterine Lavage H x 2
## 1
## Uterine swab-J
## 98
## Uterine swab-J , add 30/12
## 1
## Uterine swab-J , Smear Q x 3 , Histo H x 1
## 1
## uterine swab-L
## 1
## Uterine swab-L
## 1
## uterus fluid H x 1
## 1
## uterus swab-J
## 1
## Uterus swab-J
## 1
## uterus swab-J x 1
## 1
## Uterus swab-J x 1
## 1
## Vaginal swab-J
## 20

summary(Year2015)

## ï..Job_Number ClientCode JobDate Species PostCode
## Min. :396877 Min. : 19 22/10/2015: 5 EQU:149 Min. :6055
## 1st Qu.:410760 1st Qu.: 139 28/10/2015: 5 1st Qu.:6121
## Median :416392 Median : 301 3/11/2015 : 5 Median :6171
## Mean :416024 Mean : 489 17/11/2015: 4 Mean :6195
## 3rd Qu.:421679 3rd Qu.: 975 20/10/2015: 4 3rd Qu.:6290
## Max. :429543 Max. :1131 20/11/2015: 4 Max. :6530
## (Other) :122
## Specimens Prefix Culture
## Uterine swab-J :98 F : 0 Non haem E. coli :45
## Vaginal swab-J :20 M1:149 Beta haem. Streptococcus :27
## Cervical swab-J : 4 U : 0 Haem E.coli : 8
## Uterine Fluid H x 1 : 4 Serratia marcescens : 5
## Uterine Lavage H x 1 : 2 Staphylococcus pseudintermedius: 5
## 1 x Uterine Container: 1 Klebsiella pneumoniae : 4
## (Other) :20 (Other) :55
## Amikacin Amoxycillin Ampicillin Bactracin Carbenicillin Ceftazidime Ceftiofur
## :148 :147 : 2 :149 :148 :148 : 1
## R: 0 I: 0 R:40 S: 0 R: 1 R: 0 R: 14
## S: 1 R: 1 S:86 S: 0 S: 1 S:125
## X: 0 S: 1 X:21 X: 0 X: 0 X: 9
## X: 0
##
##
## Cefuroxime Cephalexin Chloramphenicol Ciprofloxacin Clavulox Clavulox_Clavulox
## :149 :148 :148 :147 :148 :149
## S: 0 R: 0 R: 0 I: 1 R: 0 S: 0
## S: 1 S: 1 R: 0 S: 1
## X: 0 X: 0 S: 1 X: 0
##
##
##
## Clindamycin Cloxacillin Doxcycline Doxycycline Doxyycline Enrofloxacin
## :149 :149 :149 :148 :149 : 2
## R: 0 R: 0 R: 0 I: 0 S: 0 I: 27
## S: 0 S: 0 S: 0 R: 0 R: 17
## X: 0 X: 0 S: 1 S:103
## X: 0
##
##
## Enrofloxaxin Erythromycin Fusidic_Acid Gentamicin Lincomycin Marbofloxacin
## :149 :147 :149 : 0 :149 :146
## R: 0 R: 0 R: 0 I: 2 R: 0 I: 1
## S: 2 S: 0 R:23 S: 0 R: 0
## X: 0 X: 0 S:87 S: 2
## X:37
##
##
## Minocycline Mupirocin Neomycin Norfloxacin Novobiocin Ofloxacin Penicillin
## :149 :148 : 1 :147 :149 :149 :147
## S: 0 R: 1 I: 1 R: 1 R: 0 R: 0 R: 1
## S: 0 R:47 S: 1 S: 0 S: 0 S: 1
## S:63 X: 0
## X:37
##
##
## Penicillin_G Pipercillin Polymyxin_B Rifampicin Soframycin Streptomycin
## : 3 :149 :149 :148 :149 Mode:logical
## R:27 S: 0 R: 0 R: 1 R: 0 NA's:149
## S:50 S: 0 S: 0 S: 0
## X:69 X: 0 X: 0 X: 0
##
##
##
## Sulphatrimethoprim Tetracycline Ticarcillin Timentin TestDate
## : 0 : 1 :148 : 3 Mode:logical
## R: 34 I: 0 R: 1 I: 2 NA's:149
## S:106 R:46 S: 0 R: 19
## X: 9 S:95 X: 0 S:116
## X: 7 X: 9
##
##
## ULeucocytes UEpithelial URBC UCasts
## :149 :149 :149 :149
## <1 : 0 <1 : 0 <1 : 0 None seen: 0
## >200 : 0 1 : 0 >200: 0
## 135 : 0 18 : 0 1 : 0
## 2 : 0 2 : 0 116 : 0
## 29 : 0 36 : 0 3 : 0
## (Other): 0 (Other): 0
## UCrystals UBacteria
## :149 :149
## 1+ Ca carbonate : 0 1+ : 0
## 3+ Ca carbonate, Occ Ca oxalate : 0 3+ : 0
## 3+ calcium carbonate : 0 Few : 0
## 3+ Calcium carbonate, few calcium oxalate: 0 Occasional: 0
## Ca carbonate 3+ : 0
## (Other) : 0
## USpermatoza UFungal UDebris UGram USG
## Mode:logical :149 :149 Mode:logical Min. : NA
## NA's:149 2+ Hyphae: 0 1+: 0 NA's:149 1st Qu.: NA
## 3+: 0 Median : NA
## Mean :NaN
## 3rd Qu.: NA
## Max. : NA
## NA's :149
## UPh UProtein UGlucose UKetones UBlood
## :149 :149 :149 :149 :149
## >=9.0: 0 1+ : 0 3+ : 0 1+ : 0 1+ : 0
## 6 : 0 2+ : 0 Negative: 0 Negative: 0 3+ : 0
## 8 : 0 3+ : 0 Trace : 0 Trace : 0 Negative: 0
## 8.5 : 0 Negative: 0
## 9 : 0
##
## UBilirubin UCultureNote M1Leucocytes M1RBC
## :149 :149 :148 :148
## 1+ : 0 >10,000 CFU/mL : 0 NONE SEEN: 1 VERY OCCASIONAL: 1
## 3+ : 0 >100,000 CFU/mL: 0 1+ : 0 1+ : 0
## Negative: 0 2,000 CFU/mL : 0 2+ : 0 2+ : 0
## 3,200 CFU/mL : 0 3+ : 0 3+ : 0
## 4,000 CFU/mL : 0 FEW : 0 FEW : 0
## (Other) : 0 (Other) : 0 (Other) : 0
## M1Epithelial M1Fungal FMicroForExcess
## :148 :148 Mode:logical
## NONE SEEN: 1 NONE SEEN : 1 NA's:149
## 1+ : 0 2+ fungal hyphae : 0
## 2+ : 0 2+ Yeast cells and Fungal Hyphae: 0
## 3+ : 0 FEW * : 0
## FEW : 0 FEW FUNGAL HYPHAE : 0
## (Other) : 0 (Other) : 0
## FLeucocytes FRBC FCampylobacter FYeast
## :149 :149 :149 :149
## None seen: 0 None seen: 0 None seen: 0 None seen : 0
## Very occasional: 0
##
##
##
##
## FProtozoaCystsOva FTrypsin10 FTrypsin100 FOccultBlood
## :149 Mode:logical Mode:logical Mode:logical
## None seen: 0 NA's:149 NA's:149 NA's:149
##
##
##
##
##

Year2016 <- repro_only[grepl("2016",repro_only$JobDate),]
Year2016$JobDate <- as.factor(as.character(Year2016$JobDate))
Year2016$Specimens <- as.factor(as.character(Year2016$Specimens))
summary(Year2016$JobDate)

## 20/12/2016 18/10/2016 30/8/2016 26/10/2016 15/11/2016 4/11/2016 7/9/2016
## 12 9 6 5 4 4 4
## 13/12/2016 18/11/2016 19/11/2016 2/11/2016 25/11/2016 5/10/2016 6/12/2016
## 3 3 3 3 3 3 3
## 8/10/2016 1/12/2016 11/1/2016 11/10/2016 11/8/2016 12/7/2016 13/9/2016
## 3 2 2 2 2 2 2
## 17/12/2016 19/1/2016 19/12/2016 20/1/2016 21/11/2016 21/12/2016 23/1/2016
## 2 2 2 2 2 2 2
## 23/9/2016 24/11/2016 25/8/2016 27/10/2016 28/10/2016 28/11/2016 3/11/2016
## 2 2 2 2 2 2 2
## 3/9/2016 31/12/2016 5/11/2016 8/1/2016 8/9/2016 9/11/2016 9/12/2016
## 2 2 2 2 2 2 2
## 1/1/2016 1/11/2016 1/2/2016 1/3/2016 1/7/2016 10/10/2016 10/11/2016
## 1 1 1 1 1 1 1
## 10/9/2016 11/11/2016 12/1/2016 12/10/2016 12/12/2016 12/9/2016 13/2/2016
## 1 1 1 1 1 1 1
## 14/1/2016 14/10/2016 14/11/2016 15/12/2016 16/11/2016 16/12/2016 16/8/2016
## 1 1 1 1 1 1 1
## 17/10/2016 18/1/2016 18/4/2016 18/8/2016 19/3/2016 19/8/2016 19/9/2016
## 1 1 1 1 1 1 1
## 2/2/2016 2/9/2016 20/10/2016 22/1/2016 22/10/2016 22/9/2016 24/9/2016
## 1 1 1 1 1 1 1
## 25/10/2016 26/2/2016 26/8/2016 27/1/2016 28/12/2016 28/7/2016 28/9/2016
## 1 1 1 1 1 1 1
## 29/11/2016 29/12/2016 29/4/2016 29/6/2016 29/9/2016 3/3/2016 30/11/2016
## 1 1 1 1 1 1 1
## 30/12/2016 4/1/2016 5/1/2016 5/9/2016 6/1/2016 6/10/2016 6/2/2016
## 1 1 1 1 1 1 1
## 7/4/2016 (Other)
## 1 3

summary(Year2016$Specimens)

## 1 x J premate
## 7
## 1 x Premate
## 1
## 1x J cliterus
## 1
## Blood D x 1 , Blood B x 1 , Uterine swab-J , Smear Q x 2
## 1
## Blood D x 1, Blood B x 1, Uterine swab-J
## 1
## Cervical swab-J
## 13
## Cervix J x 1
## 1
## Cervix swab J x 1
## 1
## Clitoral Swab J x 1
## 1
## Endometrial Swab J x 1 , Histo H x 1
## 1
## Pre Mating Swab J x 1
## 1
## Premate swab- J
## 3
## premate swab-J x 1
## 1
## Premate Swab 1xJ`
## 1
## Premate swab J
## 1
## Premate Swab J x 1
## 5
## Premate Swab pot x 1
## 2
## Premating Swab J x 1
## 3
## Urterine swab- J
## 1
## Uterine Fluid D x 1
## 1
## Uterine fluid H
## 1
## uterine fluid H x 1
## 1
## Uterine Fluid H x 1 , add 29/11
## 1
## uterine fluid tube x 2 , add 29/11
## 1
## uterine fluid tubes x 2
## 1
## Uterine Fluid x H
## 1
## Uterine Flush H x 1
## 2
## Uterine Lavage 50ml x 2
## 1
## Uterine lavage H x 1, add 13/12
## 1
## Uterine swab- J
## 1
## Uterine swab-J
## 113
## Uterine swab-J , Smear Q x 2 , add 18/1
## 1
## Uterine swab-J,
## 1
## Uterine swab-J, Uterine swab- L
## 1
## uterine swab-K
## 1
## Uterine Swab 1xJ
## 1
## Uterine swab J x 1
## 1
## Uterine Swab J x 1
## 1
## Vaginal swab-J
## 4

summary(Year2016)

## ï..Job_Number ClientCode JobDate Species PostCode
## Min. :430057 Min. : 3.0 20/12/2016: 12 EQU:182 Min. :6055
## 1st Qu.:485719 1st Qu.: 160.0 18/10/2016: 9 1st Qu.:6121
## Median :496233 Median : 168.0 30/8/2016 : 6 Median :6171
## Mean :487224 Mean : 499.8 26/10/2016: 5 Mean :6212
## 3rd Qu.:503938 3rd Qu.: 975.0 15/11/2016: 4 3rd Qu.:6290
## Max. :512932 Max. :1200.0 4/11/2016 : 4 Max. :6931
## (Other) :142
## Specimens Prefix Culture Amikacin
## Uterine swab-J :113 F : 0 Non haem E. coli :52 :182
## Cervical swab-J : 13 M1:182 Beta haem. Streptococcus :26 R: 0
## 1 x J premate : 7 U : 0 Beta haem. Streptococcus#:23 S: 0
## Premate Swab J x 1: 5 Serratia marcescens :11 X: 0
## Vaginal swab-J : 4 Enterococcus sp. : 8
## Premate swab- J : 3 Klebsiella pneumoniae : 6
## (Other) : 37 (Other) :56
## Amoxycillin Ampicillin Bactracin Carbenicillin Ceftazidime Ceftiofur
## :177 : 5 :182 :182 :182 : 0
## I: 0 R: 34 S: 0 R: 0 R: 0 R: 16
## R: 2 S:114 S: 0 S: 0 S:151
## S: 3 X: 29 X: 0 X: 0 X: 15
## X: 0
##
##
## Cefuroxime Cephalexin Chloramphenicol Ciprofloxacin Clavulox Clavulox_Clavulox
## :182 :182 :182 :182 :182 :182
## S: 0 R: 0 R: 0 I: 0 R: 0 S: 0
## S: 0 S: 0 R: 0 S: 0
## X: 0 X: 0 S: 0 X: 0
##
##
##
## Clindamycin Cloxacillin Doxcycline Doxycycline Doxyycline Enrofloxacin
## :182 :182 :182 :182 :182 : 5
## R: 0 R: 0 R: 0 I: 0 S: 0 I: 46
## S: 0 S: 0 S: 0 R: 0 R: 7
## X: 0 X: 0 S: 0 S:124
## X: 0
##
##
## Enrofloxaxin Erythromycin Fusidic_Acid Gentamicin Lincomycin Marbofloxacin
## :182 :177 :182 : 0 :182 :182
## R: 0 R: 1 R: 0 I: 0 R: 0 I: 0
## S: 2 S: 0 R:21 S: 0 R: 0
## X: 2 X: 0 S:95 S: 0
## X:66
##
##
## Minocycline Mupirocin Neomycin Norfloxacin Novobiocin Ofloxacin Penicillin
## :182 :182 : 0 :182 :182 :182 :177
## S: 0 R: 0 I: 0 R: 0 R: 0 R: 0 R: 1
## S: 0 R:56 S: 0 S: 0 S: 0 S: 2
## S:60 X: 2
## X:66
##
##
## Penicillin_G Pipercillin Polymyxin_B Rifampicin Soframycin Streptomycin
## : 5 :182 :182 :182 :182 Mode:logical
## R:32 S: 0 R: 0 R: 0 R: 0 NA's:182
## S:78 S: 0 S: 0 S: 0
## X:67 X: 0 X: 0 X: 0
##
##
##
## Sulphatrimethoprim Tetracycline Ticarcillin Timentin TestDate
## : 0 : 0 :182 : 10 Mode:logical
## R: 30 I: 0 R: 0 I: 0 NA's:182
## S:137 R:79 S: 0 R: 21
## X: 15 S:97 X: 0 S:135
## X: 6 X: 16
##
##
## ULeucocytes UEpithelial URBC UCasts
## :182 :182 :182 :182
## <1 : 0 <1 : 0 <1 : 0 None seen: 0
## >200 : 0 1 : 0 >200: 0
## 135 : 0 18 : 0 1 : 0
## 2 : 0 2 : 0 116 : 0
## 29 : 0 36 : 0 3 : 0
## (Other): 0 (Other): 0
## UCrystals UBacteria
## :182 :182
## 1+ Ca carbonate : 0 1+ : 0
## 3+ Ca carbonate, Occ Ca oxalate : 0 3+ : 0
## 3+ calcium carbonate : 0 Few : 0
## 3+ Calcium carbonate, few calcium oxalate: 0 Occasional: 0
## Ca carbonate 3+ : 0
## (Other) : 0
## USpermatoza UFungal UDebris UGram USG
## Mode:logical :182 :182 Mode:logical Min. : NA
## NA's:182 2+ Hyphae: 0 1+: 0 NA's:182 1st Qu.: NA
## 3+: 0 Median : NA
## Mean :NaN
## 3rd Qu.: NA
## Max. : NA
## NA's :182
## UPh UProtein UGlucose UKetones UBlood
## :182 :182 :182 :182 :182
## >=9.0: 0 1+ : 0 3+ : 0 1+ : 0 1+ : 0
## 6 : 0 2+ : 0 Negative: 0 Negative: 0 3+ : 0
## 8 : 0 3+ : 0 Trace : 0 Trace : 0 Negative: 0
## 8.5 : 0 Negative: 0
## 9 : 0
##
## UBilirubin UCultureNote M1Leucocytes M1RBC
## :182 :182 :182 :182
## 1+ : 0 >10,000 CFU/mL : 0 1+ : 0 1+ : 0
## 3+ : 0 >100,000 CFU/mL: 0 2+ : 0 2+ : 0
## Negative: 0 2,000 CFU/mL : 0 3+ : 0 3+ : 0
## 3,200 CFU/mL : 0 FEW : 0 FEW : 0
## 4,000 CFU/mL : 0 NONE SEEN: 0 NONE SEEN: 0
## (Other) : 0 (Other) : 0 (Other) : 0
## M1Epithelial M1Fungal FMicroForExcess
## :182 :182 Mode:logical
## 1+ : 0 2+ fungal hyphae : 0 NA's:182
## 2+ : 0 2+ Yeast cells and Fungal Hyphae: 0
## 3+ : 0 FEW * : 0
## FEW : 0 FEW FUNGAL HYPHAE : 0
## NONE SEEN: 0 FUNGAL HYPHAE 1+ : 0
## (Other) : 0 (Other) : 0
## FLeucocytes FRBC FCampylobacter FYeast
## :182 :182 :182 :182
## None seen: 0 None seen: 0 None seen: 0 None seen : 0
## Very occasional: 0
##
##
##
##
## FProtozoaCystsOva FTrypsin10 FTrypsin100 FOccultBlood
## :182 Mode:logical Mode:logical Mode:logical
## None seen: 0 NA's:182 NA's:182 NA's:182
##
##
##
##
##

Year2017 <- repro_only[grepl("2017",repro_only$JobDate),]
Year2017$JobDate <- as.factor(as.character(Year2017$JobDate))
Year2017$Specimens <- as.factor(as.character(Year2017$Specimens))
summary(Year2017$JobDate)

## 1/12/2017 1/3/2017 1/9/2017 10/1/2017 10/11/2017 10/2/2017 11/10/2017
## 1 1 1 1 1 1 1
## 11/2/2017 12/1/2017 12/12/2017 12/4/2017 12/9/2017 13/1/2017 13/10/2017
## 1 1 1 1 3 2 2
## 13/11/2017 13/12/2017 13/9/2017 14/10/2017 14/11/2017 14/3/2017 14/8/2017
## 4 1 1 1 2 1 1
## 14/9/2017 15/11/2017 15/12/2017 15/9/2017 16/1/2017 16/10/2017 16/11/2017
## 1 1 1 4 1 1 3
## 16/2/2017 16/8/2017 17/10/2017 18/11/2017 19/1/2017 19/10/2017 19/9/2017
## 1 1 3 1 2 1 1
## 2/11/2017 2/12/2017 2/2/2017 20/11/2017 20/9/2017 21/11/2017 21/4/2017
## 2 1 3 1 1 1 1
## 21/9/2017 22/11/2017 22/9/2017 23/1/2017 23/11/2017 23/12/2017 24/10/2017
## 1 1 2 1 4 1 1
## 25/10/2017 25/11/2017 25/8/2017 26/10/2017 26/8/2017 27/1/2017 27/11/2017
## 1 1 3 1 1 1 1
## 27/12/2017 27/9/2017 28/10/2017 28/11/2017 28/8/2017 28/9/2017 29/11/2017
## 1 1 1 2 1 3 3
## 29/12/2017 29/8/2017 29/9/2017 3/1/2017 3/2/2017 30/10/2017 30/11/2017
## 1 2 1 2 2 2 5
## 30/8/2017 30/9/2017 31/1/2017 31/10/2017 31/8/2017 4/1/2017 4/10/2017
## 1 2 1 1 1 1 2
## 4/11/2017 4/12/2017 4/2/2017 5/1/2017 5/10/2017 5/12/2017 5/9/2017
## 4 1 1 1 2 2 1
## 6/1/2017 6/11/2017 6/9/2017 7/10/2017 7/11/2017 7/8/2017 7/9/2017
## 1 1 2 2 2 2 1
## 8/12/2017 9/10/2017 9/11/2017 9/12/2017 9/2/2017 9/8/2017
## 3 1 1 1 1 1

summary(Year2017$Specimens)

## 1 x J endometrium
## 1
## 1 x J premate
## 7
## 1 x J uterine
## 1
## 1 x J uterine biopsy
## 1
## 1 x J uterine lavage
## 1
## 1 x J uterine swab
## 1
## Cervical swab-J
## 2
## Clitoral swab-J
## 1
## culture.plates uterine swab 2 x J
## 1
## EQ PM Swab J pot x 1
## 1
## Permate Swab J x 1
## 1
## premate swab J x 1
## 1
## Premate swab J x 1
## 3
## Premate Swab J x 1
## 4
## Premate Swab J x 1, Uterine Flush B x 2
## 1
## Premate swab Jx1
## 2
## Premate Swab Jx1, Uterine lavage fluid Dx1
## 1
## Premate Swab L x 1
## 1
## premate swabJ
## 3
## Premating swab Jx1, Smear Qx1
## 1
## Swab "mare" J
## 1
## Urine H x 1 , Uterine swab-J
## 1
## uterine fluid H x 1
## 1
## Uterine fluid in falcon tube
## 1
## uterine fluid tube x 1
## 1
## Uterine flush 1xTube
## 1
## Uterine Flush H pot x 1
## 1
## uterine Flush H x 1
## 2
## Uterine flush H x 1
## 5
## Uterine larvge-J
## 1
## uterine lavage fluid H x 1
## 1
## Uterine lavage H x 1
## 1
## Uterine Lavage H x 1
## 9
## Uterine lavage in 4 x 50mL tubes
## 1
## uterine lavage x 1
## 1
## Uterine swab-J
## 71
## Uterine swab-J x 1
## 1
## Uterine swab-J, Slide Q x 1
## 1
## Uterine swab-JQ
## 1
## Uterine swab-K
## 2
## Uterine swab-L
## 1
## Uterine swab J x 1, Smear Q x 2 , add 6/12
## 1
## Uterine swab J x 1
## 1
## Uterine Swab J x 1
## 1
## Uterine swab L x 1
## 1
## Vaginal swab-J
## 3
## Vulval Discharge Swab J x 1
## 1

summary(Year2017)

## ï..Job_Number ClientCode JobDate Species PostCode
## Min. :513193 Min. : 19.0 30/11/2017: 5 EQU:148 Min. :6055
## 1st Qu.:564656 1st Qu.: 101.0 13/11/2017: 4 1st Qu.:6122
## Median :574378 Median : 538.5 15/9/2017 : 4 Median :6171
## Mean :567561 Mean : 596.9 23/11/2017: 4 Mean :6193
## 3rd Qu.:586526 3rd Qu.:1200.0 4/11/2017 : 4 3rd Qu.:6214
## Max. :595802 Max. :1240.0 12/9/2017 : 3 Max. :6714
## (Other) :124
## Specimens Prefix Culture Amikacin
## Uterine swab-J :71 F : 0 Non haem E. coli :48 :147
## Uterine Lavage H x 1: 9 M1:148 Beta haem. Streptococcus :22 R: 1
## 1 x J premate : 7 U : 0 Beta haem. Streptococcus#:14 S: 0
## Uterine flush H x 1 : 5 Pseudomonas aeruginosa :10 X: 0
## Premate Swab J x 1 : 4 Enterobacter sp. : 8
## Premate swab J x 1 : 3 Serratia marcescens : 7
## (Other) :49 (Other) :39
## Amoxycillin Ampicillin Bactracin Carbenicillin Ceftazidime Ceftiofur
## :146 : 2 :148 :147 :147 : 0
## I: 0 R:36 S: 0 R: 1 R: 0 R: 17
## R: 1 S:75 S: 0 S: 1 S:119
## S: 0 X:35 X: 0 X: 0 X: 12
## X: 1
##
##
## Cefuroxime Cephalexin Chloramphenicol Ciprofloxacin Clavulox Clavulox_Clavulox
## :148 :148 :148 :148 :148 :148
## S: 0 R: 0 R: 0 I: 0 R: 0 S: 0
## S: 0 S: 0 R: 0 S: 0
## X: 0 X: 0 S: 0 X: 0
##
##
##
## Clindamycin Cloxacillin Doxcycline Doxycycline Doxyycline Enrofloxacin
## :148 :148 :148 :147 :148 : 2
## R: 0 R: 0 R: 0 I: 0 S: 0 I:45
## S: 0 S: 0 S: 0 R: 1 R:11
## X: 0 X: 0 S: 0 S:90
## X: 0
##
##
## Enrofloxaxin Erythromycin Fusidic_Acid Gentamicin Lincomycin Marbofloxacin
## :148 :146 :148 : 0 :148 :147
## R: 0 R: 1 R: 0 I: 0 R: 0 I: 0
## S: 0 S: 0 R:23 S: 0 R: 0
## X: 1 X: 0 S:87 S: 1
## X:38
##
##
## Minocycline Mupirocin Neomycin Norfloxacin Novobiocin Ofloxacin Penicillin
## :148 :148 : 0 :147 :148 :148 :146
## S: 0 R: 0 I: 0 R: 0 R: 0 R: 0 R: 1
## S: 0 R:59 S: 1 S: 0 S: 0 S: 0
## S:51 X: 1
## X:38
##
##
## Penicillin_G Pipercillin Polymyxin_B Rifampicin Soframycin Streptomycin
## : 2 :148 :148 :148 :148 Mode:logical
## R:19 S: 0 R: 0 R: 0 R: 0 NA's:148
## S:49 S: 0 S: 0 S: 0
## X:78 X: 0 X: 0 X: 0
##
##
##
## Sulphatrimethoprim Tetracycline Ticarcillin Timentin TestDate
## : 0 : 0 :147 :148 Mode:logical
## R: 31 I: 0 R: 0 I: 0 NA's:148
## S:105 R:66 S: 1 R: 0
## X: 12 S:72 X: 0 S: 0
## X:10 X: 0
##
##
## ULeucocytes UEpithelial URBC UCasts
## :147 :147 :147 :147
## <1 : 1 <1 : 1 <1 : 1 None seen: 1
## >200 : 0 1 : 0 >200: 0
## 135 : 0 18 : 0 1 : 0
## 2 : 0 2 : 0 116 : 0
## 29 : 0 36 : 0 3 : 0
## (Other): 0 (Other): 0
## UCrystals UBacteria
## :147 :147
## Calcium oxalate 3+ : 1 1+ : 0
## 1+ Ca carbonate : 0 3+ : 0
## 3+ Ca carbonate, Occ Ca oxalate : 0 Few : 1
## 3+ calcium carbonate : 0 Occasional: 0
## 3+ Calcium carbonate, few calcium oxalate: 0
## (Other) : 0
## USpermatoza UFungal UDebris UGram USG
## Mode:logical :148 :148 Mode:logical Min. :1
## NA's:148 2+ Hyphae: 0 1+: 0 NA's:148 1st Qu.:1
## 3+: 0 Median :1
## Mean :1
## 3rd Qu.:1
## Max. :1
## NA's :147
## UPh UProtein UGlucose UKetones UBlood
## :147 :147 :147 :147 :147
## >=9.0: 0 1+ : 0 3+ : 0 1+ : 0 1+ : 0
## 6 : 0 2+ : 0 Negative: 1 Negative: 1 3+ : 0
## 8 : 1 3+ : 0 Trace : 0 Trace : 0 Negative: 1
## 8.5 : 0 Negative: 1
## 9 : 0
##
## UBilirubin UCultureNote M1Leucocytes M1RBC
## :147 :148 :148 :148
## 1+ : 0 >10,000 CFU/mL : 0 1+ : 0 1+ : 0
## 3+ : 0 >100,000 CFU/mL: 0 2+ : 0 2+ : 0
## Negative: 1 2,000 CFU/mL : 0 3+ : 0 3+ : 0
## 3,200 CFU/mL : 0 FEW : 0 FEW : 0
## 4,000 CFU/mL : 0 NONE SEEN: 0 NONE SEEN: 0
## (Other) : 0 (Other) : 0 (Other) : 0
## M1Epithelial M1Fungal FMicroForExcess
## :148 :148 Mode:logical
## 1+ : 0 2+ fungal hyphae : 0 NA's:148
## 2+ : 0 2+ Yeast cells and Fungal Hyphae: 0
## 3+ : 0 FEW * : 0
## FEW : 0 FEW FUNGAL HYPHAE : 0
## NONE SEEN: 0 FUNGAL HYPHAE 1+ : 0
## (Other) : 0 (Other) : 0
## FLeucocytes FRBC FCampylobacter FYeast
## :148 :148 :148 :148
## None seen: 0 None seen: 0 None seen: 0 None seen : 0
## Very occasional: 0
##
##
##
##
## FProtozoaCystsOva FTrypsin10 FTrypsin100 FOccultBlood
## :148 Mode:logical Mode:logical Mode:logical
## None seen: 0 NA's:148 NA's:148 NA's:148
##
##
##
##
##

Year2018 <- repro_only[grepl("2018",repro_only$JobDate),]
Year2018$JobDate <- as.factor(as.character(Year2018$JobDate))
Year2018$Specimens <- as.factor(as.character(Year2018$Specimens))
summary(Year2018$JobDate)

## 1/11/2018 1/12/2018 1/9/2018 10/1/2018 10/10/2018 10/11/2018 10/9/2018
## 5 2 8 2 4 2 1
## 11/10/2018 11/12/2018 11/9/2018 12/1/2018 12/10/2018 12/2/2018 12/3/2018
## 3 2 3 1 1 3 1
## 12/6/2018 12/9/2018 13/11/2018 13/12/2018 13/9/2018 14/11/2018 14/2/2018
## 1 2 1 1 2 3 1
## 14/8/2018 15/1/2018 15/11/2018 16/11/2018 16/4/2018 17/2/2018 17/9/2018
## 1 2 7 1 1 2 2
## 18/1/2018 18/12/2018 18/9/2018 19/10/2018 19/11/2018 20/11/2018 20/12/2018
## 1 5 1 2 2 5 1
## 21/11/2018 21/2/2018 21/9/2018 22/10/2018 22/12/2018 23/10/2018 23/5/2018
## 1 2 8 3 2 2 1
## 23/6/2018 23/8/2018 24/1/2018 24/10/2018 24/8/2018 25/1/2018 25/10/2018
## 1 3 1 1 1 1 4
## 25/6/2018 26/11/2018 27/10/2018 27/11/2018 27/2/2018 27/6/2018 28/12/2018
## 1 3 5 1 1 1 4
## 28/8/2018 29/1/2018 29/9/2018 3/1/2018 3/11/2018 3/12/2018 3/2/2018
## 2 1 5 1 1 1 1
## 30/11/2018 30/8/2018 31/10/2018 31/8/2018 4/10/2018 4/12/2018 4/4/2018
## 3 3 1 4 2 1 1
## 4/9/2018 5/10/2018 5/11/2018 5/2/2018 5/9/2018 6/10/2018 6/11/2018
## 1 1 5 1 2 3 2
## 6/4/2018 7/2/2018 7/9/2018 8/1/2018 8/10/2018 8/11/2018 8/3/2018
## 1 1 14 2 3 6 1
## 9/10/2018
## 2

summary(Year2018$Specimens)

## 1 x J uterine 1 x J uterine lavage
## 1 3
## Cervix swab J x 1 Clit Swab J x 1
## 1 1
## clitoral swab -J Clitoral swab J x 1
## 2 3
## Clitoral swab J x 2, Blood BD x 1 Clitoral swab Jx1
## 1 3
## Clittoral swab -J Eq Premate Swab J
## 3 1
## Eq Uterine Swab J x 1 Mare Endometritis Swab J x 1
## 2 1
## Mare Flush H x 1 Pre-mating Swab Jx 1
## 1 2
## Pre mate swab Jx1 Premate Clittoral J
## 1 2
## Premate J x 1 premate swab J x 1
## 2 4
## Premate Swab J x 1 premate swab Jx1
## 4 1
## Premate Swab Sm Pot premate swabJ
## 1 1
## Uterine Fluid H x 1 Uterine Flush-H x 1
## 2 1
## Uterine flush 1x-H Uterine flush D x 1
## 1 1
## Uterine flush H x 1 uterine flush in H x 1
## 2 1
## Uterine J x 1 Uterine Lavage
## 3 3
## uterine lavage H x 1 Uterine lavage H x 1
## 1 6
## Uterine Lavage H x 1 Uterine Lavage Hx 1
## 3 1
## Uterine lavage Hx1 Uterine Lavage in 50mL Tube x 1
## 1 1
## Uterine lavage in Hx1 Uterine laverage Hx1
## 2 2
## Uterine swab-J uterine swab-J x 1
## 106 1
## Uterine swab-J x 1 Uterine swab-J, Smear Q x 1
## 1 8
## Uterine swab-J, Uterine biopsy H x 1 Uterine swab-J, Uterine lavage H x 1
## 1 2
## uterine swab-K Uterine swab-L
## 2 1
## uterine swab - K x 1 Uterine swab J x 1
## 1 1
## Uterine Swab J x 1 Uterine Swab L x 1
## 4 1

summary(Year2018)

## ï..Job_Number ClientCode JobDate Species PostCode
## Min. :596731 Min. : 23.0 7/9/2018 : 14 EQU:201 Min. :6069
## 1st Qu.:650121 1st Qu.: 160.0 1/9/2018 : 8 1st Qu.:6121
## Median :657652 Median : 692.0 21/9/2018 : 8 Median :6171
## Mean :655380 Mean : 631.7 15/11/2018: 7 Mean :6232
## 3rd Qu.:677176 3rd Qu.:1131.0 8/11/2018 : 6 3rd Qu.:6214
## Max. :688333 Max. :1240.0 1/11/2018 : 5 Max. :6931
## (Other) :153
## Specimens Prefix Culture
## Uterine swab-J :106 F : 0 Beta haem. Streptococcus:49
## Uterine swab-J, Smear Q x 1: 8 M1:201 Non haem E. coli :44
## Uterine lavage H x 1 : 6 U : 0 Enterococcus sp. :17
## premate swab J x 1 : 4 Non haem E. coli* : 5
## Premate Swab J x 1 : 4 Pseudomonas aeruginosa : 5
## Uterine Swab J x 1 : 4 Enterobacter sp. : 4
## (Other) : 69 (Other) :77
## Amikacin Amoxycillin Ampicillin Bactracin Carbenicillin Ceftazidime Ceftiofur
## :201 :201 : 1 :201 :201 :201 : 1
## R: 0 I: 0 R: 69 S: 0 R: 0 R: 0 R: 36
## S: 0 R: 0 S:108 S: 0 S: 0 S:138
## X: 0 S: 0 X: 23 X: 0 X: 0 X: 26
## X: 0
##
##
## Cefuroxime Cephalexin Chloramphenicol Ciprofloxacin Clavulox Clavulox_Clavulox
## :201 :201 :201 :200 :201 :201
## S: 0 R: 0 R: 0 I: 0 R: 0 S: 0
## S: 0 S: 0 R: 1 S: 0
## X: 0 X: 0 S: 0 X: 0
##
##
##
## Clindamycin Cloxacillin Doxcycline Doxycycline Doxyycline Enrofloxacin
## :201 :201 :201 :201 :201 : 1
## R: 0 R: 0 R: 0 I: 0 S: 0 I: 82
## S: 0 S: 0 S: 0 R: 0 R: 11
## X: 0 X: 0 S: 0 S:107
## X: 0
##
##
## Enrofloxaxin Erythromycin Fusidic_Acid Gentamicin Lincomycin Marbofloxacin
## :201 :201 :201 : 1 :201 :201
## R: 0 R: 0 R: 0 I: 0 R: 0 I: 0
## S: 0 S: 0 R:33 S: 0 R: 0
## X: 0 X: 0 S:93 S: 0
## X:74
##
##
## Minocycline Mupirocin Neomycin Norfloxacin Novobiocin Ofloxacin Penicillin
## :201 :201 : 1 :201 :201 :201 :201
## S: 0 R: 0 I: 0 R: 0 R: 0 R: 0 R: 0
## S: 0 R:66 S: 0 S: 0 S: 0 S: 0
## S:60 X: 0
## X:74
##
##
## Penicillin_G Pipercillin Polymyxin_B Rifampicin Soframycin Streptomycin
## : 1 :201 :201 :201 :201 Mode:logical
## R:46 S: 0 R: 0 R: 0 R: 0 NA's:201
## S:80 S: 0 S: 0 S: 0
## X:74 X: 0 X: 0 X: 0
##
##
##
## Sulphatrimethoprim Tetracycline Ticarcillin Timentin TestDate
## : 1 : 1 :201 :201 Mode:logical
## R: 51 I: 0 R: 0 I: 0 NA's:201
## S:122 R: 82 S: 0 R: 0
## X: 27 S:113 X: 0 S: 0
## X: 5 X: 0
##
##
## ULeucocytes UEpithelial URBC UCasts
## :200 :200 :200 :200
## 29 : 1 1 : 1 <1 : 0 None seen: 1
## <1 : 0 <1 : 0 >200: 0
## >200 : 0 18 : 0 1 : 1
## 135 : 0 2 : 0 116 : 0
## 2 : 0 36 : 0 3 : 0
## (Other): 0 (Other): 0
## UCrystals UBacteria
## :200 :201
## None seen : 1 1+ : 0
## 1+ Ca carbonate : 0 3+ : 0
## 3+ Ca carbonate, Occ Ca oxalate : 0 Few : 0
## 3+ calcium carbonate : 0 Occasional: 0
## 3+ Calcium carbonate, few calcium oxalate: 0
## (Other) : 0
## USpermatoza UFungal UDebris UGram USG
## Mode:logical :201 :201 Mode:logical Min. :1.005
## NA's:201 2+ Hyphae: 0 1+: 0 NA's:201 1st Qu.:1.005
## 3+: 0 Median :1.005
## Mean :1.005
## 3rd Qu.:1.005
## Max. :1.005
## NA's :200
## UPh UProtein UGlucose UKetones UBlood
## :200 :201 :201 :201 :201
## >=9.0: 0 1+ : 0 3+ : 0 1+ : 0 1+ : 0
## 6 : 1 2+ : 0 Negative: 0 Negative: 0 3+ : 0
## 8 : 0 3+ : 0 Trace : 0 Trace : 0 Negative: 0
## 8.5 : 0 Negative: 0
## 9 : 0
##
## UBilirubin UCultureNote M1Leucocytes M1RBC
## :201 :200 :201 :201
## 1+ : 0 TO FOLLOW : 1 1+ : 0 1+ : 0
## 3+ : 0 >10,000 CFU/mL : 0 2+ : 0 2+ : 0
## Negative: 0 >100,000 CFU/mL: 0 3+ : 0 3+ : 0
## 2,000 CFU/mL : 0 FEW : 0 FEW : 0
## 3,200 CFU/mL : 0 NONE SEEN: 0 NONE SEEN: 0
## (Other) : 0 (Other) : 0 (Other) : 0
## M1Epithelial M1Fungal FMicroForExcess
## :201 :201 Mode:logical
## 1+ : 0 2+ fungal hyphae : 0 NA's:201
## 2+ : 0 2+ Yeast cells and Fungal Hyphae: 0
## 3+ : 0 FEW * : 0
## FEW : 0 FEW FUNGAL HYPHAE : 0
## NONE SEEN: 0 FUNGAL HYPHAE 1+ : 0
## (Other) : 0 (Other) : 0
## FLeucocytes FRBC FCampylobacter FYeast
## :201 :201 :201 :201
## None seen: 0 None seen: 0 None seen: 0 None seen : 0
## Very occasional: 0
##
##
##
##
## FProtozoaCystsOva FTrypsin10 FTrypsin100 FOccultBlood
## :201 Mode:logical Mode:logical Mode:logical
## None seen: 0 NA's:201 NA's:201 NA's:201
##
##
##
##
##

Year2019 <- repro_only[grepl("2019",repro_only$JobDate),]
Year2019$JobDate <- as.factor(as.character(Year2019$JobDate))
Year2019$Specimens <- as.factor(as.character(Year2019$Specimens))
summary(Year2019$JobDate)

## 1/10/2019 1/3/2019 10/5/2019 11/10/2019 11/11/2019 11/12/2019 12/10/2019
## 2 1 1 2 1 1 1
## 12/12/2019 12/3/2019 13/11/2019 13/3/2019 13/6/2019 13/8/2019 14/5/2019
## 2 2 3 1 2 2 1
## 15/4/2019 16/1/2019 16/10/2019 16/8/2019 16/9/2019 17/5/2019 17/9/2019
## 1 1 1 1 2 1 3
## 18/10/2019 18/9/2019 19/11/2019 19/12/2019 2/1/2019 2/10/2019 2/11/2019
## 1 1 1 2 3 4 1
## 2/12/2019 2/9/2019 20/6/2019 21/10/2019 21/11/2019 21/12/2019 21/2/2019
## 1 3 2 3 4 2 1
## 21/9/2019 22/1/2019 22/10/2019 22/8/2019 23/12/2019 23/2/2019 24/1/2019
## 1 1 2 1 4 1 1
## 24/7/2019 24/9/2019 25/10/2019 26/10/2019 26/11/2019 26/8/2019 27/2/2019
## 1 1 3 1 4 1 3
## 28/10/2019 28/11/2019 28/3/2019 28/8/2019 28/9/2019 29/10/2019 29/11/2019
## 1 3 2 5 4 2 3
## 29/8/2019 3/1/2019 3/12/2019 3/9/2019 30/10/2019 30/11/2019 31/10/2019
## 1 2 2 2 3 1 1
## 31/7/2019 31/8/2019 4/1/2019 4/10/2019 4/12/2019 5/1/2019 5/10/2019
## 2 3 1 4 3 1 2
## 5/2/2019 5/3/2019 5/9/2019 6/11/2019 6/9/2019 7/11/2019 7/2/2019
## 1 3 2 5 6 4 3
## 7/8/2019 8/10/2019 8/11/2019 8/5/2019 9/1/2019 9/10/2019 9/11/2019
## 1 2 5 1 1 1 2
## 9/12/2019 9/9/2019
## 5 1

summary(Year2019$Specimens)

## 1 x H uterine lavage
## 1
## 1 x J premate
## 2
## 1 x premate J
## 4
## 1 x premate swab
## 2
## 50 ml tube uterine lavage
## 1
## Cervical swab-J
## 2
## Cervical Swab J x 1
## 2
## cliteral swab J x 1
## 1
## Clitoral Dry Swab K x 1
## 1
## Clitoral swab x 1
## 5
## Clitorial swab-K
## 2
## Histo H x 1 , Uterine Tissue H x 1
## 1
## Mare swab J x 1
## 1
## PM swab J x 1
## 3
## Premate swab - small pot x1
## 1
## Premate swab J
## 1
## premate swab J x 1
## 8
## Premate Swab J x 1
## 9
## premate swabJ
## 2
## Prematen Swab K x 1, Premate Swab J x 1
## 3
## uterine lavage
## 2
## Uterine lavage
## 1
## Uterine lavage-J, Agar media-H
## 2
## uterine lavage H
## 1
## uterine lavage H x 1
## 1
## Uterine lavage H x 1
## 6
## Uterine Lavage H x 1
## 9
## Uterine Lavage Hx1
## 3
## Uterine swab-J
## 71
## Uterine swab-J , Hair H x 1 , Uterine Lavage H x 1
## 2
## Uterine swab-K
## 2
## Uterine swab-L
## 2
## Uterine Swab J x 1
## 15
## Uterine swabs x3-J
## 2
## Uterus lesion swab-J
## 2
## uterus swab 1xJ
## 1
## Vag discharge H x 1
## 2
## Vaginal swab-J
## 1

summary(Year2019)

## ï..Job_Number ClientCode JobDate Species PostCode
## Min. :688970 Min. : 19.0 6/9/2019 : 6 EQU:177 Min. :6069
## 1st Qu.:739301 1st Qu.: 160.0 28/8/2019: 5 1st Qu.:6112
## Median :750397 Median : 308.0 6/11/2019: 5 Median :6163
## Mean :743190 Mean : 575.2 8/11/2019: 5 Mean :6182
## 3rd Qu.:758832 3rd Qu.:1131.0 9/12/2019: 5 3rd Qu.:6214
## Max. :769338 Max. :1240.0 2/10/2019: 4 Max. :6935
## (Other) :147
## Specimens Prefix Culture Amikacin
## Uterine swab-J :71 F : 0 Non haem E. coli :58 :177
## Uterine Swab J x 1 :15 M1:177 Beta haem. Streptococcus:40 R: 0
## Premate Swab J x 1 : 9 U : 0 Enterococcus sp. :16 S: 0
## Uterine Lavage H x 1: 9 Enterobacter sp.* : 5 X: 0
## premate swab J x 1 : 8 Enterobacter sp. : 3
## Uterine lavage H x 1: 6 Haem E.coli : 3
## (Other) :59 (Other) :52
## Amoxycillin Ampicillin Bactracin Carbenicillin Ceftazidime Ceftiofur
## :172 : 6 :177 :177 :177 : 1
## I: 0 R: 55 S: 0 R: 0 R: 0 R: 28
## R: 1 S:100 S: 0 S: 0 S:129
## S: 2 X: 16 X: 0 X: 0 X: 19
## X: 2
##
##
## Cefuroxime Cephalexin Chloramphenicol Ciprofloxacin Clavulox Clavulox_Clavulox
## :177 :177 :177 :177 :177 :177
## S: 0 R: 0 R: 0 I: 0 R: 0 S: 0
## S: 0 S: 0 R: 0 S: 0
## X: 0 X: 0 S: 0 X: 0
##
##
##
## Clindamycin Cloxacillin Doxcycline Doxycycline Doxyycline Enrofloxacin
## :177 :177 :177 :177 :177 : 6
## R: 0 R: 0 R: 0 I: 0 S: 0 I:59
## S: 0 S: 0 S: 0 R: 0 R:16
## X: 0 X: 0 S: 0 S:96
## X: 0
##
##
## Enrofloxaxin Erythromycin Fusidic_Acid Gentamicin Lincomycin Marbofloxacin
## :177 :172 :177 : 1 :177 :177
## R: 0 R: 0 R: 0 I: 0 R: 0 I: 0
## S: 1 S: 0 R:47 S: 0 R: 0
## X: 4 X: 0 S:68 S: 0
## X:61
##
##
## Minocycline Mupirocin Neomycin Norfloxacin Novobiocin Ofloxacin Penicillin
## :177 :177 : 1 :177 :177 :177 :171
## S: 0 R: 0 I: 0 R: 0 R: 0 R: 0 R: 1
## S: 0 R:76 S: 0 S: 0 S: 0 S: 0
## S:40 X: 5
## X:60
##
##
## Penicillin_G Pipercillin Polymyxin_B Rifampicin Soframycin Streptomycin
## : 7 :177 :177 :177 :177 Mode:logical
## R:33 S: 0 R: 0 R: 0 R: 0 NA's:177
## S:62 S: 0 S: 0 S: 0
## X:75 X: 0 X: 0 X: 0
##
##
##
## Sulphatrimethoprim Tetracycline Ticarcillin Timentin TestDate
## : 1 : 1 :177 :177 Mode:logical
## R: 49 I: 0 R: 0 I: 0 NA's:177
## S:108 R:78 S: 0 R: 0
## X: 19 S:93 X: 0 S: 0
## X: 5 X: 0
##
##
## ULeucocytes UEpithelial URBC UCasts
## :177 :177 :177 :177
## <1 : 0 <1 : 0 <1 : 0 None seen: 0
## >200 : 0 1 : 0 >200: 0
## 135 : 0 18 : 0 1 : 0
## 2 : 0 2 : 0 116 : 0
## 29 : 0 36 : 0 3 : 0
## (Other): 0 (Other): 0
## UCrystals UBacteria
## :177 :177
## 1+ Ca carbonate : 0 1+ : 0
## 3+ Ca carbonate, Occ Ca oxalate : 0 3+ : 0
## 3+ calcium carbonate : 0 Few : 0
## 3+ Calcium carbonate, few calcium oxalate: 0 Occasional: 0
## Ca carbonate 3+ : 0
## (Other) : 0
## USpermatoza UFungal UDebris UGram USG
## Mode:logical :177 :177 Mode:logical Min. : NA
## NA's:177 2+ Hyphae: 0 1+: 0 NA's:177 1st Qu.: NA
## 3+: 0 Median : NA
## Mean :NaN
## 3rd Qu.: NA
## Max. : NA
## NA's :177
## UPh UProtein UGlucose UKetones UBlood
## :177 :177 :177 :177 :177
## >=9.0: 0 1+ : 0 3+ : 0 1+ : 0 1+ : 0
## 6 : 0 2+ : 0 Negative: 0 Negative: 0 3+ : 0
## 8 : 0 3+ : 0 Trace : 0 Trace : 0 Negative: 0
## 8.5 : 0 Negative: 0
## 9 : 0
##
## UBilirubin UCultureNote M1Leucocytes M1RBC
## :177 :177 :176 :176
## 1+ : 0 >10,000 CFU/mL : 0 OCCASIONAL: 1 3+ : 1
## 3+ : 0 >100,000 CFU/mL: 0 1+ : 0 1+ : 0
## Negative: 0 2,000 CFU/mL : 0 2+ : 0 2+ : 0
## 3,200 CFU/mL : 0 3+ : 0 FEW : 0
## 4,000 CFU/mL : 0 FEW : 0 NONE SEEN: 0
## (Other) : 0 (Other) : 0 (Other) : 0
## M1Epithelial M1Fungal FMicroForExcess
## :176 :176 Mode:logical
## NONE SEEN: 1 NONE SEEN : 1 NA's:177
## 1+ : 0 2+ fungal hyphae : 0
## 2+ : 0 2+ Yeast cells and Fungal Hyphae: 0
## 3+ : 0 FEW * : 0
## FEW : 0 FEW FUNGAL HYPHAE : 0
## (Other) : 0 (Other) : 0
## FLeucocytes FRBC FCampylobacter FYeast
## :177 :177 :177 :177
## None seen: 0 None seen: 0 None seen: 0 None seen : 0
## Very occasional: 0
##
##
##
##
## FProtozoaCystsOva FTrypsin10 FTrypsin100 FOccultBlood
## :177 Mode:logical Mode:logical Mode:logical
## None seen: 0 NA's:177 NA's:177 NA's:177
##
##
##
##
##

Year2020 <- repro_only[grepl("2020",repro_only$JobDate),]
Year2020$JobDate <- as.factor(as.character(Year2020$JobDate))
Year2020$Specimens <- as.factor(as.character(Year2020$Specimens))
summary(Year2020$JobDate)

## 1/2/2020 10/1/2020 12/3/2020 13/1/2020 14/2/2020 14/5/2020 17/1/2020 18/1/2020
## 1 2 4 3 1 1 2 1
## 28/1/2020 3/2/2020 3/3/2020 30/1/2020 31/1/2020 5/5/2020 9/1/2020
## 1 1 1 2 3 2 1

summary(Year2020$Specimens)

## Blood D x 1 , Blood B x 1 , Blood F x 1 , Blood E x 1 , Uterine swab-J
## 1
## clitoral swab J x 1
## 3
## Premate Swab J x 1
## 1
## Uterine flush H x 1, Uterine swab-J
## 2
## Uterine swab-J
## 14
## Uterine swab-J, Smear Q x 2
## 2
## uterine swab-L
## 1
## Uterine swab-L
## 1
## Vaginal swab-J, Tissue x 1 H
## 1

summary(Year2020)

## ï..Job_Number ClientCode JobDate Species PostCode
## Min. :772270 Min. : 52.0 12/3/2020: 4 EQU:26 Min. :6084
## 1st Qu.:774085 1st Qu.: 101.0 13/1/2020: 3 1st Qu.:6121
## Median :777158 Median : 160.0 31/1/2020: 3 Median :6171
## Mean :780349 Mean : 346.4 10/1/2020: 2 Mean :6220
## 3rd Qu.:786564 3rd Qu.: 616.8 17/1/2020: 2 3rd Qu.:6231
## Max. :801232 Max. :1131.0 30/1/2020: 2 Max. :6566
## (Other) :10
## Specimens
## Uterine swab-J :14
## clitoral swab J x 1 : 3
## Uterine flush H x 1, Uterine swab-J : 2
## Uterine swab-J, Smear Q x 2 : 2
## Blood D x 1 , Blood B x 1 , Blood F x 1 , Blood E x 1 , Uterine swab-J: 1
## Premate Swab J x 1 : 1
## (Other) : 3
## Prefix Culture Amikacin Amoxycillin Ampicillin
## F : 0 Beta haem. Streptococcus :7 :25 :26 : 0
## M1:26 Non haem E. coli :6 R: 0 I: 0 R: 9
## U : 0 Enterobacter sp. :2 S: 1 R: 0 S:14
## Enterococcus sp. :2 X: 0 S: 0 X: 3
## Actinobacillus suis/equuli:1 X: 0
## Klebsiella pneumoniae :1
## (Other) :7
## Bactracin Carbenicillin Ceftazidime Ceftiofur Cefuroxime Cephalexin
## :26 :25 :25 : 0 :26 :26
## S: 0 R: 1 R: 1 R: 5 S: 0 R: 0
## S: 0 S: 0 S:19 S: 0
## X: 0 X: 0 X: 2 X: 0
##
##
##
## Chloramphenicol Ciprofloxacin Clavulox Clavulox_Clavulox Clindamycin
## :26 :25 :26 :26 :26
## R: 0 I: 1 R: 0 S: 0 R: 0
## S: 0 R: 0 S: 0 S: 0
## X: 0 S: 0 X: 0 X: 0
##
##
##
## Cloxacillin Doxcycline Doxycycline Doxyycline Enrofloxacin Enrofloxaxin
## :26 :26 :26 :26 : 0 :26
## R: 0 R: 0 I: 0 S: 0 I: 8 R: 0
## S: 0 S: 0 R: 0 R: 2
## X: 0 S: 0 S:16
## X: 0
##
##
## Erythromycin Fusidic_Acid Gentamicin Lincomycin Marbofloxacin Minocycline
## :26 :26 : 0 :26 :25 :26
## R: 0 R: 0 I: 0 R: 0 I: 0 S: 0
## S: 0 S: 0 R:11 S: 0 R: 0
## X: 0 X: 0 S: 5 S: 1
## X:10
##
##
## Mupirocin Neomycin Norfloxacin Novobiocin Ofloxacin Penicillin Penicillin_G
## :26 :6 :25 :26 :26 :26 : 0
## R: 0 I:0 R: 0 R: 0 R: 0 R: 0 R: 4
## S: 0 R:8 S: 1 S: 0 S: 0 S: 0 S:11
## S:5 X: 0 X:11
## X:7
##
##
## Pipercillin Polymyxin_B Rifampicin Soframycin Streptomycin
## :26 :26 :26 :26 Mode:logical
## S: 0 R: 0 R: 0 R: 0 NA's:26
## S: 0 S: 0 S: 0
## X: 0 X: 0 X: 0
##
##
##
## Sulphatrimethoprim Tetracycline Ticarcillin Timentin TestDate
## : 0 : 0 :26 :26 Mode:logical
## R:10 I: 0 R: 0 I: 0 NA's:26
## S:14 R:15 S: 0 R: 0
## X: 2 S:11 X: 0 S: 0
## X: 0 X: 0
##
##
## ULeucocytes UEpithelial URBC UCasts
## :26 :26 :26 :26
## <1 : 0 <1 : 0 <1 : 0 None seen: 0
## >200 : 0 1 : 0 >200: 0
## 135 : 0 18 : 0 1 : 0
## 2 : 0 2 : 0 116 : 0
## 29 : 0 36 : 0 3 : 0
## (Other): 0 (Other): 0
## UCrystals UBacteria USpermatoza
## :26 :26 Mode:logical
## 1+ Ca carbonate : 0 1+ : 0 NA's:26
## 3+ Ca carbonate, Occ Ca oxalate : 0 3+ : 0
## 3+ calcium carbonate : 0 Few : 0
## 3+ Calcium carbonate, few calcium oxalate: 0 Occasional: 0
## Ca carbonate 3+ : 0
## (Other) : 0
## UFungal UDebris UGram USG UPh UProtein
## :26 :26 Mode:logical Min. : NA :26 :26
## 2+ Hyphae: 0 1+: 0 NA's:26 1st Qu.: NA >=9.0: 0 1+ : 0
## 3+: 0 Median : NA 6 : 0 2+ : 0
## Mean :NaN 8 : 0 3+ : 0
## 3rd Qu.: NA 8.5 : 0 Negative: 0
## Max. : NA 9 : 0
## NA's :26
## UGlucose UKetones UBlood UBilirubin UCultureNote
## :26 :26 :26 :26 :26
## 3+ : 0 1+ : 0 1+ : 0 1+ : 0 >10,000 CFU/mL : 0
## Negative: 0 Negative: 0 3+ : 0 3+ : 0 >100,000 CFU/mL: 0
## Trace : 0 Trace : 0 Negative: 0 Negative: 0 2,000 CFU/mL : 0
## 3,200 CFU/mL : 0
## 4,000 CFU/mL : 0
## (Other) : 0
## M1Leucocytes M1RBC M1Epithelial
## :26 :26 :26
## 1+ : 0 1+ : 0 1+ : 0
## 2+ : 0 2+ : 0 2+ : 0
## 3+ : 0 3+ : 0 3+ : 0
## FEW : 0 FEW : 0 FEW : 0
## NONE SEEN: 0 NONE SEEN: 0 NONE SEEN: 0
## (Other) : 0 (Other) : 0 (Other) : 0
## M1Fungal FMicroForExcess FLeucocytes
## :26 Mode:logical :26
## 2+ fungal hyphae : 0 NA's:26 None seen: 0
## 2+ Yeast cells and Fungal Hyphae: 0
## FEW * : 0
## FEW FUNGAL HYPHAE : 0
## FUNGAL HYPHAE 1+ : 0
## (Other) : 0
## FRBC FCampylobacter FYeast FProtozoaCystsOva
## :26 :26 :26 :26
## None seen: 0 None seen: 0 None seen : 0 None seen: 0
## Very occasional: 0
##
##
##
##
## FTrypsin10 FTrypsin100 FOccultBlood
## Mode:logical Mode:logical Mode:logical
## NA's:26 NA's:26 NA's:26
##
##
##
##
##
